# Cascade Cyclization/Annulation of β-Enamino Diketones and *o*-Phenylenediamine: A Strategy to Access Pyrrole-fused 1,5-Benzodiazepines

**DOI:** 10.1021/acs.joc.4c02483

**Published:** 2024-11-13

**Authors:** Julia Poletto, Julia C. M. Willig, Jeniffer N. A. Camargo, Helio G. Bonacorso, Michael J. V. Silva, Fernanda A. Rosa

**Affiliations:** † Laboratory for Synthesis of Heterocycles (SINTHET), Chemistry Department, State University of Maringá - UEM, Maringá, Paraná 87020-900, Brazil; ‡ Núcleo de Química de Heterociclos (NUQUIMHE), Chemistry Department, Federal University of Santa Maria - UFSM, Santa Maria, Rio Grande do Sul 97105-900, Brazil; § Federal Technological University of Paraná - UTFPR, Toledo, Paraná 85902-490, Brazil

## Abstract

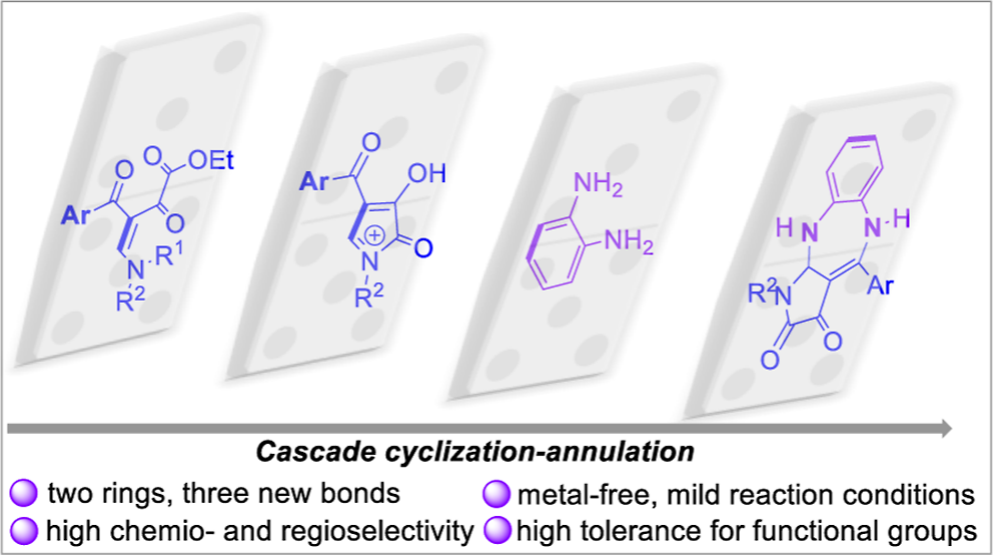

Herein, we introduce an unprecedented cascade reaction for the assembly of pyrrole-fused 1,5-benzodiazepine frameworks. These diverse privileged scaffolds were controllably constructed by intramolecular cyclization of β-enamino diketone, followed by annulation with *o*-phenylenediamine. The protocol features efficient one-pot cascade cyclization/annulation, performed under simple and mild reaction conditions. The products are obtained in a metal-free manner, in good to excellent yields (65–91%), and represent a fused heterocyclic scaffold that is not yet found in nature.

## Introduction

**Figure 1:**
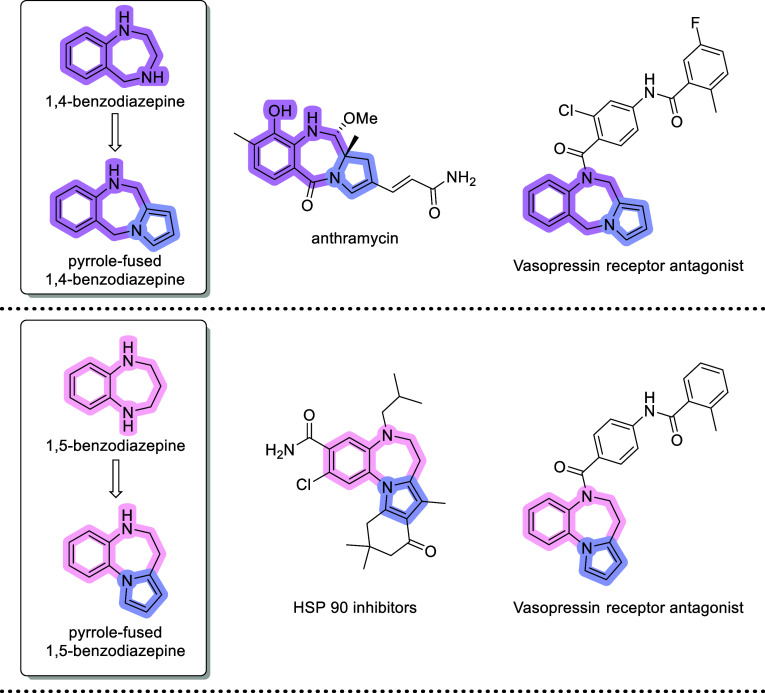
Selected bioactive compounds containing pyrrole-fused benzodiazepine scaffolds.

Pyrrole-fused benzodiazepines are a canonical category of *N*-heterocycles with a broad spectrum of biological properties.^1^ In particular, the pyrrole-fused 1,4-benzodiazepine (1,4-PBD) framework is present in a plethora of natural bioactive molecules. Since anthramycin was isolated in the 1960s, the chemistry and biology of synthetic 1,4-PBDs have been extensively studied.^2^ Although an innovative pyrrole-fused 1,5-benzodiazepine (1,5-PBD) scaffold is found in arginine vasopressin receptor antagonists and heat shock protein (HSP) 90 inhibitors (Figure 1), relatively few synthetic methods are available to access this fused scaffold.^3^

**Scheme 1:**
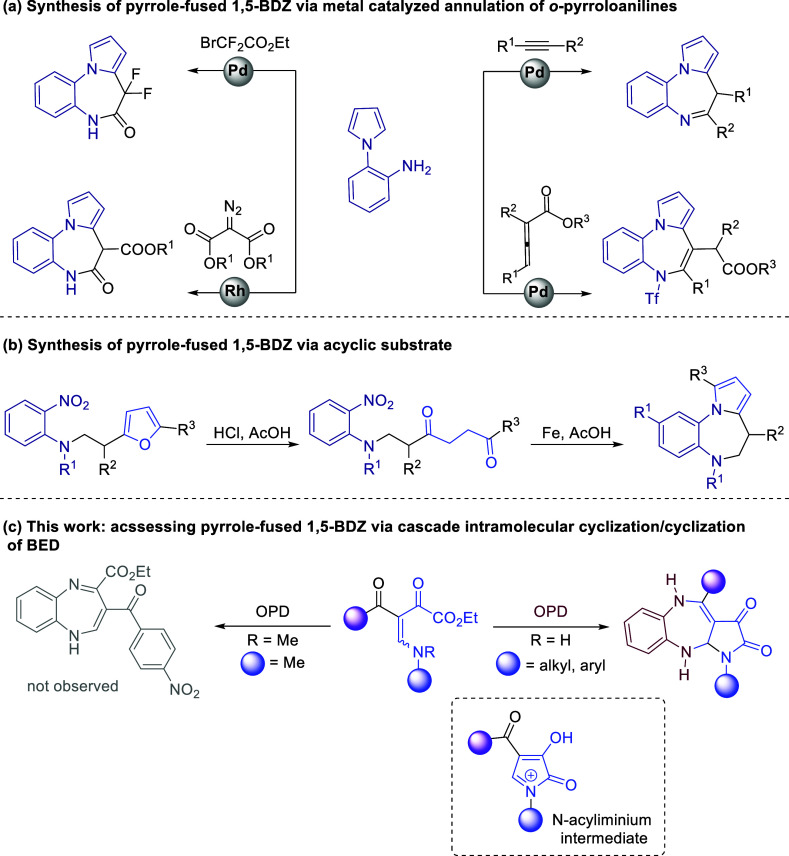
Literature Approaches to Pyrrole-fused 1,5-BDZ Derivatives

The conventional method for synthesizing 1,5-PDB involves the construction of a 1,5-diazepine core on a substrate that includes a preformed pyrrole ring. For instance, metal-catalyzed annulation of 2-(1*H*-pyrrol-1-yl)anilines^4−7^ (Scheme 1a). In addition, the Paal-Knorr condensation/cyclization sequence has been reported using an acyclic precursor containing amino and carbonyl functionalities. Because of the incompatibility of these groups, substituted furans as synthetic equivalents of 1,4-dicarbonyl compounds and the latent form of the amino group are required for these methods^8^ (Scheme 1b). However, very few options are available for the simultaneous construction of both rings in a single step.

Based on these analyses, the development of a more efficient and versatile method for the synthesis of a pyrrole-fused 1,5-benzodiazepine system, particularly one that can provide direct access to a benzodiazepine moiety fused with a pyrrole ring, is still extremely desirable. In this context, cascade reactions have emerged as powerful tools that often allow the synthesis of highly functionalized molecules from relatively simple substrates in a single step, without the separation and purification of intermediates.

β-enamino diketones (BEDs) are versatile and important polyfunctionalized building blocks that can act as C–C–C synthons in formal cyclization reactions with different reactive partners, leading to selective construction of structurally diverse five- and six-membered nitrogen heterocycles.^9−11^ However, the use of BED as a three-atom synthon to form benzodiazepine frameworks has been poorly explored. Following our continuous interest in the development of synthetic methodology involving this versatile polyfunctionalized precursor, we report herein our results on the reaction of BED and *o*-phenylenediamine (OPD) via cyclization reactions cascade (Scheme 1c).

## Results and Discussion

**Scheme 2:**
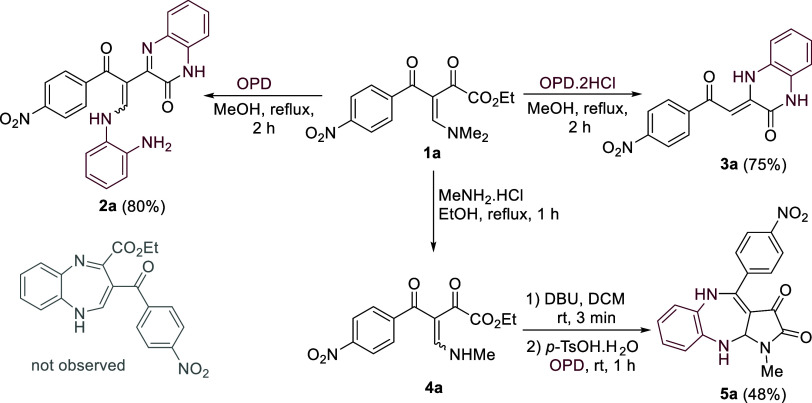
Proof of Concept

Our investigation began with an examination of the reaction between BED **1a** and OPD in methanol under reflux.^12^ The reaction was completed in 2 h; however, surprisingly, six-membered quinoxalinone **2a** was obtained in 80% yield. Interestingly, when diamine was used in the form of dihydrochloride, quinoxalinone **3a**(13) was obtained as the sole product in 75% yield. Notably, both tests resulted in the formation of a six-membered ring, which was formally obtained by the cyclocondensation at the α-oxo ester moiety of **1a** (Scheme 2). To prevent the reaction from occurring at the 1,2-dielectrophilic center of BED, we envisioned employing secondary BED **4a** to generate the 4-acyl-pyrrole-2,3-dione cyclic derivative.^14^ Thus, BED **4a** was subjected to intramolecular cyclization using DCM and DBU (1.2 equiv) at room temperature for 3 min. Surprisingly, the sequential treatment with *p*-TsOH.H_2_O (PTSA) (2.2 equiv) and OPD (1.2 equiv) for 1 h resulted in the formation of an unanticipated fused heterocyclic pyrrolo[5,4-*b*][1,5]benzodiazepine **5a** in 48% yield (Scheme 2).

Next, the optimal cascade reaction conditions were determined (Table 1). Among the solvents tested, MeOH was the most suitable for the multistep cascade cyclization in terms of the yield of **5a** (61%) (Table 1, entries 1–3). Therefore, we evaluated the effect of *p*-TsOH.H_2_O using methanol as the solvent. Variation in amount of *p*-TsOH.H_2_O revealed that a stoichiometric amount (1.2 equiv) improved the yield of **5a** (79%), although the reaction time was extended to 24 h (Table 1, entry 4). However, the reaction did not occur with a substoichiometric amount of PTSA (0.6 equiv) even after 72 h (Table 1, entry 5). Furthermore, when *p*-TsOH.H_2_O and OPD were employed in excess (2.2 equiv), the yield of **5a** dramatically improved to 91% in a short time period, 2 h (Table 1, entry 6).

**Table 1:**
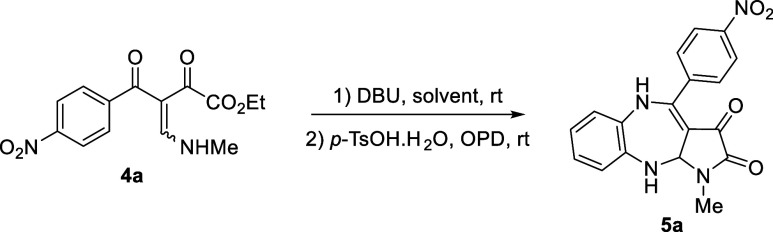
Optimization of Cascade Cyclization/Condensation/Cyclization Reaction Conditions^a^

entry	solvent	DBU equiv	PTSA equiv	OPD equiv	time (h) step1/step2	yield (%)^b^
1	MeCN	1.2	2.2	1.2	2/1	40
2	EtOH	1.2	2.2	1.2	2/2	54
3	MeOH	1.2	2.2	1.2	1/1	61
4	MeOH	1.2	1.2	1.2	1/24	79
5	MeOH	1.2	0.6	1.2	1/72	-c
6	MeOH	1.2	2.2	2.2	1/2	91
7	MeOH	1.2	1.2	2.2	1/24	50

a Reaction conditions: **4a** (1.0 mmol, 1 equiv), DBU, 15 mL of solvent, room temperature, time 1; after completion of the reaction PTSA, OPD, room temperature, time 2.

b Isolated yield.

c Full consumption of OPD was not observed.

**Scheme 3:**
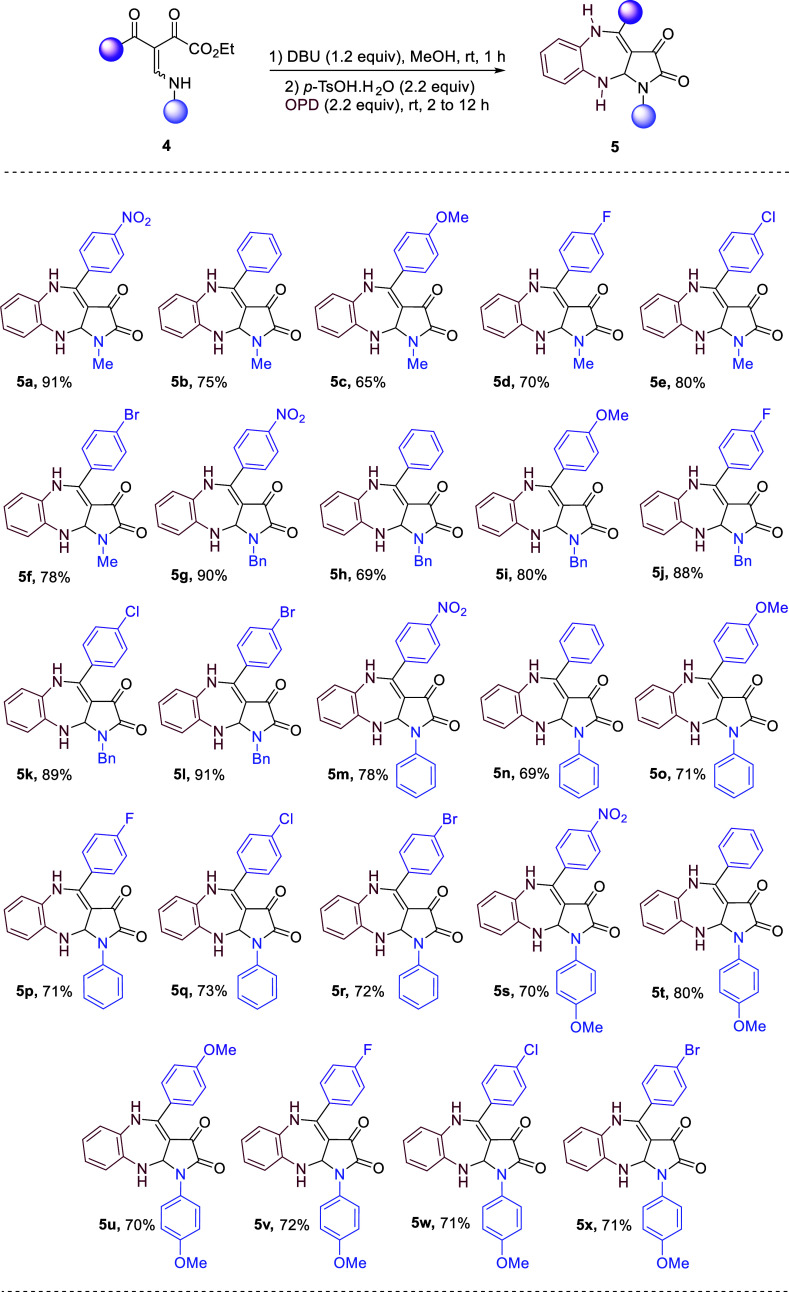
Exploration of Substrate Scope^,^ Reaction conditions: **4** (1.0 mmol), DBU (1.2 mmol, 1.2 equiv), 15 mL MeOH, rt, 1 h; After completion of reaction *p*-TsOH.H_2_O (2.2 mmol, 2.2 equiv), OPD (2.2 mmol, 2.2 equiv), rt, 2 to 12 h. Isolated yields.

With the optimal conditions in hand, we investigated the scope of cascade cyclization reactions with various enamino diketone substrates **4** (Scheme 3). Initially, we explored the generality of the reaction by changing the substituents at the *para*-position of the benzoyl moiety in **4**. The halogen, electron-withdrawing, donating, and neutral substituents were well tolerated and produced **5a**–**f** in good to excellent yields (65–91%). Additionally, we explored the variation in substituents at the secondary nitrogen atom of BED **4**. The *N*-benzyl group did not affect the multistep cascade cyclization process and successfully afforded the respective products **5g**–**l** in yields of 69% to 91%. Expanding to *N*-aryl groups, substrates bearing phenyl and *p*-OMe phenyl similarly led to the corresponding products **5m**–**x** in 69–80% of yields. It is noteworthy that our cascade intramolecular cyclization/annulation strategy was crucial to the outcome of the reaction in terms of product selectivity.

**Scheme 4:**
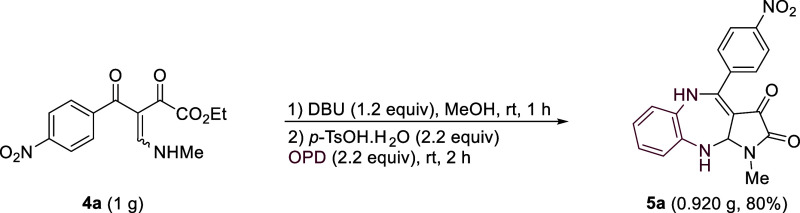
Gram-Scale Synthesis

After assessing the substrate scope, a scale-up of **4a** was conducted under standard conditions. This reaction performes effectively on a gram scale, affording product **5a** in 80% yield (Scheme 4).

**Scheme 5:**
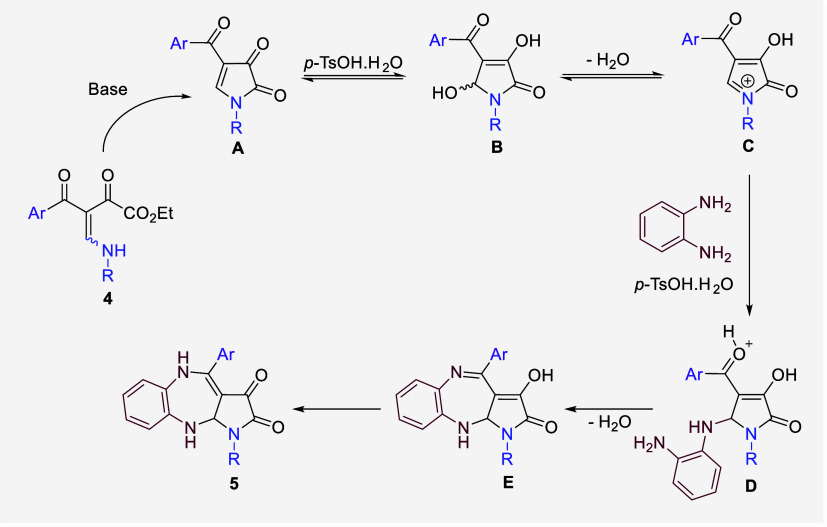
Plausible Mechanism

Based on these observations and literature reports,^15^ we suggest a reasonable mechanism for the formation of pyrrole-fused 1,5-benzodiazepine scaffold **5**. First, the DBU base is thought to promote intramolecular cyclization of secondary BED **4**, yielding 4-acyl-1*H*-pyrrole-2,3-dione cyclic intermediate **A**. In the presence the acid and water, *N*-acyliminium species **C** is generated, which then undergoes nucleophilic attack by OPD. The acid-activated intermediate **D** subsequently undergoes 7-*exo*-trig cyclization to form intermediate **E**. This is followed by an enol-imine to keto-enamine tautomerization, ultimately leading to the formation of pyrrolo[5,4-*b*][1,5]benzodiazepine **5** (Scheme 5). It is noteworthy that *p*-TsOH.H_2_O significantly accelerates the cyclization reaction to form intermediate **E**, although a substantial quantity (2.2 equiv) is required to achieve this effect. The requirement for excess acid is consistent with prior literature reports.^16^

## Conclusion

In summary, we have developed a versatile and efficient approach for accessing structurally complex pyrrolo[5,4-*b*][1,5]benzodiazepines through cascade cyclization/condensation/cyclization reactions under simple reaction conditions. With interesting selectivity, the reaction afforded excellent chemo- and regioselective syntheses of pyrrole-fused 1,5-benzodiazepine derivatives from a polyfunctionalized precursor and *o*-phenylenediamine. This strategy provides a direct protocol for the expansion of the chemical space of small molecules, which may be of biological interest.

## Experimental Section

### General Information

Reagents were used as obtained from commercial suppliers without further purification. Solvents were dried and purified according to recommended procedures. The reactions were monitored by thin-layer chromatography using Merck TLC silica gel plates and visualized with UV light. All melting points were measured with the MQAPF-307 Microquímica apparatus using benzoic acid as the internal standard. ^1^H NMR and ^13^C{^1^H} NMR experiments were run on Bruker Avance III HD apparatus operating at ^1^H 300.06 MHz and ^13^C 75.46 MHz or Bruker Avance III HD apparatus operating at ^1^H 500.13 MHz and ^13^C 125.77 MHz and all structural assignments were made with additional information from gHSQC and gHMBC experiments. Chemical shifts are reported in ppm using DMSO-*d*_6_. ESI(+)–MS and tandem ESI(+)–MS/MS were acquired using a hybrid high-resolution and high accuracy microTof (Q-TOF) mass spectrometer (Bruker). For ESI(+)–MS, the energy for the collision-induced dissociations (CDI) was optimized for each component. For data acquisition and processing, the Q-TOF-control data analysis software (Bruker Scientific) was used.

### Preparation of Starting Materials

The synthesis of the required starting materials **1**(17) and **4**(10,14,18) followed the previous literature procedures.

### General Synthetic Procedure

#### Synthesis of 3-(1-((2-Aminophenyl)amino)-3-(4-nitrophenyl)3-oxoprop-1-en-2-yl)quinoxaline-2(1*H*)-one (**2a**)

To a solution of β-enamino diketone **1a** (1.0 mmol, 1.0 equiv, 0.320 g) in methanol (15 mL), was added *o*-phenylenediamine (2.2 mmol, 2.2 equiv, 0.238 g) and the mixture was stirred under reflux in an oil bath for 2 h (monitored by TLC). Then, the mixture was cooled to 0 °C and the solid was filtered, washed with cold methanol (10 mL) and dried under vacuum to provide the product **2a**.

#### 3-(1-((2-Aminophenyl)amino)-3-(4-nitrophenyl)-3-oxoprop-1-en-2-yl)quinoxalin-2(1*H*)-one (**2a**)

Orange solid; yield: 80%, 0.342 g; mp: 250.1–250.6 °C; mixture of *Z* and *E* - 30:70. ^**1**^**H NMR** (DMSO-*d*_6_, 300.06 MHz): *Z* isomer: δ 12.15 (s, 1H), 11.63 (d, *J* = 14.0 Hz, 1H), 8.40 (d, *J* = 13.2 Hz, 1H), 8.16 (d, *J* = 8.9 Hz, 2H), 7.64 (d, *J* = 8.9 Hz, 2H), 7.57 (dd, *J* = 8.0, 1.4 Hz, 1H), 7.46–6.70 (m, 7H), 5.11 (s, 2H). *E* isomer: δ 12.20 (s, 1H), 11.59 (d, *J* = 13.6 Hz, 1H), 8.23 (d, *J* = 8.9 Hz, 2H), 8.04 (d, *J* = 13.1 Hz, 1H), 7.85 (d, *J* = 8.9 Hz, 2H), 7.81 (dd, *J* = 8.1, 1.5 Hz, 1H), 7.46–6.70 (m, 7H), 5.07 (s, 2H). ^**13**^**C{**^**1**^**H} NMR** (DMSO-*d*_6_, 125.77 MHz): *Z* and *E* isomers: δ 191.2, 190.8, 156.6, 155.7, 154.6, 154.0, 150.9, 148.5, 147.9, 147.8, 146.7, 145.5, 139.6, 139.2, 132.3, 131.8, 131.6, 130, 128.7, 128.5, 128.0, 127.9, 127.5, 127.3, 126.0, 125.2, 123.5, 123.4, 118.6, 117.4, 117.1, 115.1, 115.0, 107.4, 106.7, 89.5; **HRMS** (ESI) *m*/*z*: [M + Na]^+^ calcd for C_23_H_17_N_5_O_4_Na^+^, 450.1178; found, 450.1183.

#### Synthesis of 3-(2-(4-Nitrophenyl)-2-oxoethylidene)-3,4-dihydroquinoxalin-2(1*H*)-one (**3a**)

To a solution of β-enamino diketone **1a** (1.0 mmol, 1.0 equiv, 0.320 g) in methanol (15 mL), *o*-phenylenediamine dihydrochloride (1.2 mmol, 1.2 equiv, 0.217 g) was added and the reaction was refluxed in an oil bath for 2 h (monitored by TLC). Then, the mixture was cooled to 0 °C and the solid was filtered, washed with cold methanol (10 mL) and dried under vacuum to provide **3a** in 75% yield (0.232 g). Compound **3a** has been previously reported.^13^

#### General Procedure for the Synthesis of Pyrrole[5,4-*b*][1,5]benzodiazepines (**5a**–***x***)

To a solution of β-enamino diketone **4** (1.0 mmol, 1.0 equiv) 4**a** (Ar = 4-NO_2_C_6_H_4_, R = Me): 0.306 g, **4b** (Ar = Ph, R = Me): 0.261 g, **4c** (Ar = 4-OMeC_6_H_4_, R = Me): 0.291 g, **4d** (Ar = 4-FC_6_H_4_, R = Me): 0.279 g, **4e** (Ar = 4-ClC_6_H_4_, R = Me): 0.295 g, **4f** (Ar = 4-BrC_6_H_4_, R = Me): 0.339 g, **4g** (Ar = 4-NO_2_C_6_H_4_, R = Bn): 0.382 g, **4h** (Ar = Ph, R = Bn): 0.337 g, **4i** (Ar = 4-OMeC_6_H_4_, R = Bn): 0.367 g, **4j** (Ar = 4-FC_6_H_4_, R = Bn): 0.355 g, **4k** (Ar = 4-ClC_6_H_4_, R = Bn): 0.371 g, **4l** (Ar = 4-BrC_6_H_4_, R = Bn): 0.415 g, **4m** (Ar = 4-NO_2_C_6_H_4_, R = Ph): 0.368 g, **4n** (Ar = Ph, R = Ph): 0.323 g, **4o** (Ar = 4-OMeC_6_H_4_, R = Ph): 0.353 g, **4p** (Ar = 4-FC_6_H_4_, R = Ph): 0.341 g, **4q** (Ar = 4-ClC_6_H_4_, R = Ph): 0.357 g, **4r** (Ar = 4-BrC_6_H_4_, R = Ph): 0.401 g, **4s** (Ar = 4-NO_2_C_6_H_4_, R = 4-OMeC_6_H_4_): 0.398 g, **4t** (Ar = 4-Ph, R = 4-OMeC_6_H_4_): 0.353 g, **4u** (Ar = 4-OMeC_6_H_4_, R = 4-OMeC_6_H_4_): 0.383 g, **4v** (Ar = 4-FC_6_H_4_, R = 4-OMeC_6_H_4_): 0.371 g, **4w** (Ar = 4-ClC_6_H_4_, R = 4-OMeC_6_H_4_): 0.387 g, **4x** (Ar = 4-BrC_6_H_4_, R = 4-OMeC_6_H_4_): 0.431 g, in methanol (15 mL), was added DBU (1.2 mmol, 1.2 equiv, 0.183 g) and the mixture was stirred at room temperature for 1 h (monitored by TLC). Then, *p*-TsOH.H_2_O (2.2 mmol, 2.2 equiv, 0.418 g) and *o*-phenylenediamine (2.2 mmol, 2.2 equiv, 0.238 g) were added and the mixture was stirred at room temperature for more 2 (Me) to 12 h (Bn and aryl). The solid was filtered, washed with cold methanol (10 mL), and dried under vacuum to afford the desired product (**5a**–**x**).

#### 1-Methyl-4-(4-nitrophenyl)-1,5,10,10a-tetrahydropyrrolo[5,4-*b*][1,5]benzodiazepine-2,3-dione (**5a**)

Orange solid; yield: 91%, 0.318 g; mp: 275.0–278.2 °C (decomposition); ^**1**^**H NMR** (DMSO-*d*_6_, 300.06 MHz, δ): 9.94 (s, 1H), 8.32 (d, *J* = 8.8 Hz, 2H), 7.73 (d, *J* = 8.8 Hz, 2H), 7.27 (dd, *J* = 8.2, 1.5 Hz, 1H), 7.23 (dd, *J* = 8.2, 1.5 Hz, 1H), 7.02 (dd, *J* = 8.2, 7.2, 1.5 Hz, 1H), 6.87 (dd, *J* = 8.5, 7.1, 1.5 Hz, 1H), 6.22 (s, 1H), 5.27 (s, 1H), 3.13 (s, 3H). ^**13**^**C{**^**1**^**H} NMR** (DMSO-*d*_6_, 75.45 MHz, δ): 177.0, 164.0, 151.1, 148.4, 141.3, 136.5, 131.1, 129.8, 125.3, 123.2, 122.5, 122.0, 121.5, 107.5, 69.3, 28.1; **HRMS** (ESI) *m*/*z*: [M + H]^+^ calcd for C_18_H_15_N_4_O_4_^+^, 351.1088; found, 351.1091.

#### 1-Methyl-4-phenyl-1,5,10,10a-tetrahydropyrrolo[5,4-*b*][1,5]benzodiazepine-2,3-dione (**5b**)

Yellow solid; yield: 75%, 0.229 g; mp: 273.2–275.8 °C (decomposition); ^**1**^**H NMR** (DMSO-*d*_6_, 300.06 MHz, δ): 9.69 (s, 1H), 7.52–7.44 (m, 5H), 7.35 (dd, *J* = 8.1, 1.5 Hz, 1H), 7.19 (dd, *J* = 8.0, 1.5 Hz, 1H), 6.98 (dd, *J* = 8.1, 7.2, 1.4 Hz, 1H), 6.83 (dd, *J* = 8.5, 7.1, 1.5 Hz, 1H), 6.17 (s, 1H), 5.27 (s, 1H), 3.12 (s, 3H). ^**13**^**C{**^**1**^**H} NMR** (DMSO-*d*_6_, 75.45 MHz, δ): 176.5, 164.2, 153.8, 136.3, 134.6, 130.2, 129.7, 129.4, 128.0, 124.8, 122.5, 121.5, 121.1, 107.2, 69.3, 28.0. **HRMS** (ESI) *m*/*z*: [M + H]^+^ calcd for C_18_H_16_N_3_O_2_^+^, 306.1237; found, 306.1248.

#### 1-Methyl-4-(4-methoxyphenyl)-1,5,10,10a-tetrahydropyrrolo[5,4-*b*][1,5]benzodiazepine-2,3-dione (**5c**)

Orange solid; yield: 65%, 0.218 g; mp: 265.7–266.8 °C (decomposition); ^**1**^**H NMR** (DMSO-*d*_6_, 300.06 MHz, δ): 9.57 (s, 1H), 7.41 (d, *J* = 8.8 Hz, 2H), 7.36 (dd, *J* = 8.1, 1.5 Hz, 1H), 7.16 (dd, *J* = 8.1, 1.5 Hz, 1H), 7.01 (d, *J* = 8.8 Hz, 2H), 6.96 (dd, *J* = 8.1, 7.2, 1.4 Hz, 1H), 6.81 (dd, *J* = 8.5, 7.1, 1.5 Hz, 1H), 6.15 (s, 1H), 5.26 (s, 1H), 3.84 (s, 3H), 3.12 (s, 3H). ^**13**^**C{**^**1**^**H} NMR** (DMSO-*d*_6_, 75.45 MHz, δ): 176.3, 164.4, 161.2, 153.9, 136.1, 131.4, 129.5, 126.4, 124.8, 122.5, 121.3, 120.9, 113.3, 107.1, 69.2, 55.4, 28.0. **HRMS** (ESI) *m*/*z*: [M + H]^+^ calcd for C_19_H_18_N_3_O_3_^+^, 336.1343; found, 336.1340.

#### 1-Methyl-4-(4-fluorophenyl)-1,5,10,10a-tetrahydropyrrolo[5,4-*b*][1,5]benzodiazepine-2,3-dione (**5d**)

Orange solid; yield: 70%, 0.226 g; mp: 297.4–299.0 °C (decomposition); ^**1**^**H NMR** (DMSO-*d*_6_, 300.06 MHz, δ): 9.70 (s, 1H), 7.51–7.49 (m, 2H), 7.33–7.30 (m, 2H), 7.28 (d, *J* = 8.9, 1H), 7.19 (dd, *J* = 8.1, 1.5 Hz, 1H), 6.98 (dd, *J* = 8.1, 7.2, 1.5 Hz, 1H), 6.84 (dd, *J* = 8.5, 7.2, 1.5 Hz, 1H), 6.17 (s, 1H), 5.25 (s, 1H), 3.12 (s, 3H). ^**13**^**C{**^**1**^**H} NMR** (DMSO-*d*_6_, 75.45 MHz, δ): 176.6, 164.2, 163.3 (d, *J* = 245 Hz), 152.6, 136.3, 132.0 (d, *J* = 9.0 Hz), 130.8 (d, *J* = 3.0 Hz), 129.6, 124.9, 122.5, 121.5, 121.1, 114.9 (d, *J* = 21.9 Hz), 107.3, 69.2, 28.0. **HRMS** (ESI) *m*/*z*: [M + H]^+^ calcd for C_18_H_15_FN_3_O_2_^+^, 324.1143; found, 324.1152.

#### 1-Methyl-4-(4-chlorophenyl)-1,5,10,10a-tetrahydropyrrolo[5,4-*b*][1,5]benzodiazepine-2,3-dione (**5e**)

Red solid; yield: 80%, 0.271 g; mp: 276.9–279.0 °C (decomposition); ^**1**^**H NMR** (DMSO-*d*_6_, 300.06 MHz, δ): 9.74 (s, 1H), 7.54 (d, *J* = 8.6 Hz, 2H), 7.47 (d, *J* = 8.6 Hz, 2H), 7.31 (dd, *J* = 7.3, 1.5 Hz, 1H), 7.20 (dd, *J* = 8.1, 1.5 Hz, 1H), 6.99 (dd, *J* = 8.1, 7.2, 1.5 Hz, 1H), 6.85 (dd, *J* = 8.4, 7.1, 1.4 Hz, 1H), 6.18 (s, 1H), 5.25 (s, 1H), 3.12 (s, 3H). ^**13**^**C{**^**1**^**H} NMR** (DMSO-*d*_6_, 75.45 MHz, δ): 176.7, 164.1, 152.3, 136.3, 134.9, 133.4, 131.4, 129.6, 128.0, 125.0, 122.5, 121.6, 121.2, 107.3, 69.2, 28.0. **HRMS** (ESI) *m*/*z*: [M + H]^+^ calcd for C_18_H_15_ClN_3_O_2_^+^, 340.0847; found, 340.0842.

#### 1-Methyl-4-(4-bromophenyl)-1,5,10,10a-tetrahydropyrrolo[5,4-*b*][1,5]benzodiazepine-2,3-dione (**5f**)

Red solid; yield: 78%, 0.299 g; mp: 276.5–279.9 °C (decomposition); ^**1**^**H NMR** (DMSO-*d*_6_, 300.06 MHz, δ): 9.74 (s, 1H), 7.68 (d, *J* = 8.4 Hz, 2H), 7.40 (d, *J* = 8.4 Hz, 2H), 7.30 (dd, *J* = 8.1, 1.4 Hz, 1H), 7.20 (dd, *J* = 8.1, 1.5 Hz, 1H), 6.99 (dd, *J* = 8.4, 7.1, 1.5 Hz, 1H), 6.84 (dd, *J* = 8.4, 7.1, 1.4 Hz, 1H), 6.18 (s, 1H), 5.24 (s, 1H), 3.12 (s, 3H). ^**13**^**C{**^**1**^**H} NMR** (DMSO-*d*_6_, 75.45 MHz, δ): 176.7, 164.1, 152.4, 136.3, 133.8, 131.6, 131.0, 129.6, 125.0, 123.7, 122.5, 121.6, 121.2, 107.3, 69.2, 28.0. **HRMS** (ESI) *m*/*z*: [M + H]^+^ calcd for C_18_H_15_BrN_3_O_2_^+^, 384.0342; found, 384.0350.

#### 1-Benzyl-4-(4-nitrophenyl)-1,5,10,10a-tetrahydropyrrolo[5,4-*b*][1,5]benzodiazepine-2,3-dione (**5g**)

Red solid; yield: 90%, 0.383 g; mp: 281.9–283.4 °C (decomposition); ^**1**^**H NMR** (DMSO-*d*_6_, 300.06 MHz, δ): 9.99 (s, 1H), 8.30 (d, *J* = 8.8 Hz, 2H), 7.75 (d, *J* = 8.8 Hz, 2H), 7.39–7.29 (m, 5H), 7.25–7.21 (m, 2H), 7.02 (dd, *J* = 8.3, 7.2, 1.4 Hz, 1H), 6.87 (dd, *J* = 8.5, 7.2, 1.4 Hz, 1H), 6.23 (s, 1H), 5.16 (d, *J* = 14.9 Hz, 1H), 4.92 (s, 1H), 4.70 (d, *J* = 14.9 Hz, 1H). ^**13**^**C{**^**1**^**H} NMR** (DMSO-*d*_6_, 75.45 MHz, δ): 176.5, 163.5, 151.6, 148.3, 141.2, 136.3, 135.6, 131.0, 130.4, 128.9, 128.5, 127.8, 125.4, 123.0, 122.6, 122.2, 121.9, 107.3, 66.4, 42.8. **HRMS** (ESI) *m*/*z*: [M + H]^+^ calcd for C_24_H_19_N_4_O_4_^+^, 427.1401; found, 427.1405.

#### 1-Benzyl-4-phenyl-1,5,10,10a-tetrahydropyrrolo[5,4-*b*][1,5]benzodiazepine-2,3-dione (**5h**)

Orange solid; yield: 69%, 0.263 g; mp: 277.0–279.5 °C (decomposition); ^**1**^**H NMR** (DMSO-*d*_6_, 300.06 MHz, δ): 9.74 (s, 1H), 7.56–7.44 (m, 5H), 7.38 (dt, *J* = 6.9, 1.5, 1.5 Hz, 1H), 7.34–7.29 (m, 5H), 7.18 (dd, *J* = 8.0 Hz, 1.5 Hz, 1H), 6.98 (dd, *J* = 8.0, 7.2, 1.4 Hz, 1H), 6.83 (dd, *J* = 8.0, 7.2, 1.4 Hz, 1H), 6.17 (s, 1H), 5.15 (d, *J* = 14.9 Hz, 1H), 4.92 (s, 1H), 4.68 (d, *J* = 14.9 Hz, 1H). ^**13**^**C{**^**1**^**H} NMR** (DMSO-*d*_6_, 75.45 MHz, δ): 176.1, 163.9, 154.3, 136.2, 135.7, 134.5, 130.3, 130.2, 129.4, 128.9, 128.4, 127.9, 127.8, 125.1, 122.6, 121.7, 121.6, 107.0, 66.4, 42.8. **HRMS** (ESI) *m*/*z*: [M + H]^+^ calcd for C_24_H_20_N_3_O_2_^+^, 382.1550; found, 382.1535.

#### 1-Benzyl-4-(4-methoxyphenyl)-1,5,10,10a-tetrahydropyrrolo[5,4-*b*][1,5]benzodiazepine-2,3-dione (**5i**)

Orange solid; yield: 80%, 0.329 g; mp: 271.6–274.0 °C (decomposition); ^**1**^**H NMR** (DMSO-*d*_6_, 300.06 MHz, δ): 9.62 (s, 1H), 7.41 (d, *J* = 8.7 Hz, 2H), 7.36–7.29 (m, 6H), 7.15 (dd, *J* = 8,0, 1.5 Hz, 1H), 7.00 (d, *J* = 8.7 Hz, 2H), 6.97–6.94 (m, 1H), 6.81 (dd, *J* = 7.6, 7.0, 1.5 Hz, 1H), 6.15 (s, 1H), 5.14 (d, *J* = 15.0 Hz, 1H), 4.92 (s, 1H), 4.66 (d, *J* = 15.0 Hz, 1H), 3.83 (s, 3H). ^**13**^**C{**^**1**^**H} NMR** (DMSO-*d*_6_, 75.45 MHz, δ): 175.9, 164.0, 161.2, 154.4, 136.0, 135.8, 131.4, 130.0, 128.9, 128.4, 127.8, 126.3, 124.9, 122.4, 121.5, 121.3, 113.2, 106.9, 66.3, 55.4, 42.8. **HRMS** (ESI) *m*/*z*: [M + H]^+^ calcd for C_25_H_22_N_3_O_3_^+^, 412.1656; found, 412.1640.

#### 1-Benzyl-4-(4-fluorophenyl)-1,5,10,10a-tetrahydropyrrolo[5,4-*b*][1,5]benzodiazepine-2,3-dione (**5j**)

Red solid; yield: 88%, 0.351 g; mp: 305.1–308.2 °C (decomposition); ^**1**^**H NMR** (DMSO-*d*_6_, 500.13 MHz, δ): 9.76 (s, 1H), 7.53–7.50 (m, 2H), 7.37–7.27 (m, 8H), 7.19 (dd, *J* = 8.2, 1.5 Hz, 1H), 6.99 (dd, *J* = 8.3, 7.2, 1.4 Hz, 1H), 6.84 (dd, *J* = 8.5, 7.2, 1.45 Hz, 1H), 6.17 (s, 1H), 5.15 (d, *J* = 14.9 Hz, 1H), 4.92 (s, 1H), 4.68 (d, *J* = 14.9 Hz, 1H). ^**13**^**C{**^**1**^**H} NMR** (DMSO-*d*_6_, 125.77 MHz, δ): 176.0, 163.6, 163.3 (d, *J* = 245.0 Hz), 153.0, 136.0, 135.5, 131.8 (d, *J* = 8.7 Hz), 130.6 (d, *J* = 3.1 Hz), 130.0, 128.7, 128.2, 127.6, 125.0, 122.4, 121.6, 121.4, 114.7 (d, *J* = 21.8 Hz), 106.8, 66.1, 42.5. **HRMS** (ESI) *m*/*z*: [M + H]^+^ calcd for C_24_H_19_FN_3_O_2_^+^, 400.1456; found, 400.1458.

#### 1-Benzyl-4-(4-chlorophenyl)-1,5,10,10a-tetrahydropyrrolo[5,4-*b*][1,5]benzodiazepine-2,3-dione (**5k**)

Red solid; yield: 89%, 0.369 g; mp: 308.2–309.8 °C (decomposition); ^**1**^**H NMR** (DMSO-*d*_6_, 500.13 MHz, δ): 9.79 (s, 1H), 7.52 (d, *J* = 8.6 Hz, 2H), 7.47 (d, *J* = 8.6 Hz, 2H), 7.36–7.32 (m, 5H), 7.27 (dd, *J* = 8.2, 1.5 Hz, 1H), 7.19 (dd, *J* = 8.1, 1.5 Hz, 1H), 6.99 (dd, *J* = 8.2, 7.3, 1.4 Hz, 1H), 6.84 (dd, *J* = 8.4, 7.2, 1.5 Hz, 1H), 6.18 (s, 1H), 5.14 (d, *J* = 14.9 Hz, 1H), 4.91 (s, 1H), 4.68 (d, *J* = 14.9 Hz, 1H). ^**13**^**C{**^**1**^**H} NMR** (DMSO-*d*_6_, 125.77 MHz, δ): 176.2, 163.7, 152.9, 136.1, 135.7, 134.9, 133.3, 131.4, 130.2, 128.9, 128.4, 128.0, 127.8, 125.2, 122.5, 121.8, 121.7, 107.1, 66.4, 42.8. **HRMS** (ESI) *m*/*z*: [M + H]^+^ calcd for C_24_H_19_ClN_3_O_2_^+^, 416.1160; found, 416.1154.

#### 1-Benzyl-4-(4-bromophenyl)-1,5,10,10a-tetrahydropyrrolo[5,4-*b*][1,5]benzodiazepine-2,3-dione (**5l**)

Orange solid; yield: 91%, 0.418 g; mp: 302.6–304.7 °C (decomposition); ^**1**^**H NMR** (DMSO-*d*_6_, 300.06 MHz, δ): 9.79 (s, 1H), 7.66 (d, *J* = 8.5 Hz, 2H), 7.40 (d, *J* = 8.5 Hz, 2H), 7.34–7.32 (m, 5H), 7.27 (dd, *J* = 8.2, 1.5 Hz, 1H), 7.19 (dd, *J* = 8.1, 1.5 Hz, 1H), 6.99 (dd, *J* = 8.2, 7.3, 1.4 Hz, 1H), 6.84 (dd, *J* = 8.2, 7.2, 1.3 Hz, 1H), 6.18 (s, 1H), 5.15 (d, *J* = 14.9 Hz, 1H), 4.90 (s, 1H), 4.68 (d, *J* = 14.9 Hz, 1H). ^**13**^**C{**^**1**^**H} NMR** (DMSO-*d*_6_, 75.45 MHz, δ): 176.0, 163.5, 152.6, 135.9, 135.4, 133.4, 131.3, 130.6, 130.0, 128.6, 128.1, 127.5, 124.9, 123.4, 122.3, 121.6, 121.4, 106.8, 66.1, 42.5. **HRMS** (ESI) *m*/*z*: [M + H]^+^ calcd for C_24_H_19_BrN_3_O_2_^+^, 460.0655; found, 460.0657.

#### 1-Phenyl-4-(4-nitrophenyl)-1,5,10,10a-tetrahydropyrrolo[5,4-*b*][1,5]benzodiazepine-2,3-dione (**5m**)

Orange solid; yield: 78%, 0.321 g; mp: 250.3–253.6 °C (decomposition); ^**1**^**H NMR** (DMSO-*d*_6_, 300.06 MHz, δ): 10.18 (s, 1H), 8.36 (d, *J* = 8.8 Hz, 2H), 7.81 (d, *J* = 8.8 Hz, 2H), 7.72 (d, *J* = 7.6 Hz, 2H), 7.55–7.52 (m, 2H), 7.34 (d, *J* = 7.6, 1.4 Hz, 2H), 7.06 (dd, *J* = 8.0, 2.0 Hz, 1H), 7.00 (dd, *J* = 7.9, 1.04 Hz, 1H), 6.93 (dd, *J* = 8.6, 6.8, 1.9 Hz, 1H), 6.12 (s, 1H), 5.87 (s, 1H). ^**13**^**C{**^**1**^**H} NMR** (DMSO-*d*_6_, 75.45 MHz, δ): 175.5, 162.5, 151.9, 148.9, 141.12, 136.4, 135.7, 131.1, 130.7, 129.1, 125.9, 125.6, 123.2, 122.7, 122.1, 121.3, 107.3, 67.1. **HRMS** (ESI) *m*/*z*: [M + H]^+^ calcd for C_23_H_17_N_4_O_4_^+^, 413.1244; found, 413.1236.

#### 1-Phenyl-4-phenyl-1,5,10,10a-tetrahydropyrrolo[5,4-*b*][1,5]benzodiazepine-2,3-dione (**5n**)

Orange solid; yield: 69%, 0.253 g; mp: 280.0–283.4 °C (decomposition); ^**1**^**H NMR** (DMSO-*d*_6_, 300.06 MHz, δ): 9.94 (s, 1H), 7.74 (dd, *J* = 8.7, 1.2 Hz, 2H), 7.58–7.54 (m, 2H), 7.52–7.49 (m, 5H), 7.41 (dd, *J* = 8.0, 1.4 Hz, 1H), 7.34–7.29 (m, 1H), 7.02 (dd, *J* = 8.0, 2.0 Hz, 1H), 6.96 (dd, *J* = 7.9, 1.4 Hz, 1H), 6.88 (dd, *J* = 7.9, 6.7, 2.0 Hz, 1H), 6.13 (s, 1H), 5.82 (s, 1H). ^**13**^**C{**^**1**^**H} NMR** (DMSO-*d*_6_, 75.45 MHz, δ): 175.0, 162.8, 154.8, 136.3, 135.8, 134.4, 130.5, 130.4, 129.5, 129.0, 128.0, 125.7, 125.2, 122.7, 122.1, 121.6, 121.1, 107.0, 67.0. **HRMS** (ESI) *m*/*z*: [M + H]^+^ calcd for C_23_H_18_N_3_O_2_^+^, 368.1394; found, 368.1404.

#### 1-Phenyl-4-(4-methoxyphenyl)-1,5,10,10a-tetrahydropyrrolo[5,4-*b*][1,5]benzodiazepine-2,3-dione (**5o**)

Orange solid; yield: 71%, 0.282 g; mp: 264.4–266.5 °C (decomposition); ^**1**^**H NMR** (DMSO-*d*_6_, 300.06 MHz, δ): 9.82 (s, 1H), 7.75 (d, *J* = 7.5 Hz, 2H), 7.54–7.46 (m, 4H), 7.42 (d, *J* = 7.5 Hz, 1H), 7.31 (t, *J* = 7.4 Hz, 1H), 7.04 (d, *J* = 8.7 Hz, 2H), 6.99–6.92 (m, 2H), 6.86 (dd, *J* = 8.5, 6.4, 2.2 Hz, 1H), 6.11 (s, 1H), 5.78 (s, 1H), 3.85 (s, 3H). ^**13**^**C{**^**1**^**H} NMR** (DMSO-*d*_6_, 75.45 MHz, δ): 174.9, 163.2, 161.5, 155.0, 136.1, 136.0, 131.6, 130.3, 129.2, 126.3, 125.9, 125.2, 122.8, 121.8, 121.5, 121.2, 113.5, 107.3, 67.1, 55.5. **HRMS** (ESI) *m*/*z*: [M + H]^+^ calcd for C_24_H_20_N_3_O_3_^+^, 398.1499; found, 398.1486.

#### 1-Phenyl-4-(4-fluorophenyl)-1,5,10,10a-tetrahydropyrrolo[5,4-*b*][1,5]benzodiazepine-2,3-dione (**5p**)

Orange solid; yield: 71%, 0.273 g; mp: 296.7–300.1 °C (decomposition); ^**1**^**H NMR** (DMSO-*d*_6_, 300.06 MHz, δ): 9.96 (s, 1H), 7.74 (dd, *J* = 8.8, 1.2 Hz, 2H), 7.61–7.56 (m, 2H), 7.54–7.49 (m, 2H), 7.40–7.29 (m, 4H), 7.03–6.94 (m, 2H), 6.89 (dd, *J* = 8.6, 6.8, 2.0 Hz, 1H), 6.12 (s, 1H), 5.83 (s, 1H). ^**13**^**C{**^**1**^**H} NMR** (DMSO-*d*_6_, 75.45 MHz, δ): 175.1, 163.5 (d, *J* = 245.3 Hz), 162.8, 154.6, 153.7, 136.2, 135.8, 132.1 (d, *J* = 9.2 Hz), 130.7 (d, *J* = 3.0 Hz), 130.5, 129.1, 125.8, 125.3, 122.7, 122.2, 121.7, 121.2, 115.0 (d, *J* = 22.0 Hz), 107.1, 67.0. **HRMS** (ESI) *m*/*z*: [M + H]^+^ calcd for C_23_H_17_FN_3_O_2_^+^, 386.1299; found, 386.1299.

#### 1-Phenyl-4-(4-chlorophenyl)-1,5,10,10a-tetrahydropyrrolo[5,4-*b*][1,5]benzodiazepine-2,3-dione (**5q**)

Orange solid; yield: 73%, 0.293 g; mp: 296.7–299.6 °C (decomposition); ^**1**^**H NMR** (DMSO-*d*_6_, 300.06 MHz, δ): 9.99 (s, 1H), 7.73 (d, *J* = 7.6 Hz, 2H), 7.56 (d, *J* = 4.3 Hz, 4H), 7.51 (d, *J* = 8.5 Hz, 2H), 7.37 (dd, *J* = 8.0, 1.4 Hz, 1H), 7.34–7.29 (m, 1H), 7.02 (dd, *J* = 8.1, 2.2 Hz, 1H), 6.97 (dd, *J* = 7.9, 1.4 Hz, 1H), 6.90 (dd, *J* = 8.5, 6.8, 1.9 Hz, 1H), 6.11 (s, 1H), 5.83 (s, 1H). ^**13**^**C{**^**1**^**H} NMR** (DMSO-*d*_6_, 75.45 MHz, δ): 175.2, 162.7, 153.3, 136.3, 135.1, 133.2, 131.5, 130.5, 129.1, 128.1, 125.8, 125.8, 125.3, 122.6, 122.3, 121.8, 121.2, 107.1, 67.0. **HRMS** (ESI) *m*/*z*: [M + H]^+^ calcd for C_23_H_17_ClN_3_O_2_^+^, 402.1004; found, 402.1002.

#### 1-Phenyl-4-(4-bromophenyl)-1,5,10,10a-tetrahydropyrrolo[5,4-*b*][1,5]benzodiazepine-2,3-dione (**5r**)

Orange solid; yield: 72%, 0.320 g; mp: 290.1–292.5 °C (decomposition); ^**1**^**H NMR** (300.06 MHz, δ): 9.99 (s, 1H), 7.73–7.70 (m, 4H), 7.53 (d, *J* = 7.8 Hz, 2H), 7.47 (d, *J* = 8.5 Hz, 2H), 7.36 (dd, *J* = 8.0, 1.4 Hz, 1H), 7.30 (d, *J* = 9.1 Hz, 1H), 7.01–6.94 (m, 2H), 6.88 (dd, *J* = 8.6, 6.7, 1.9 Hz, 1H), 6.10 (s, 1H), 5.83 (*s*, 1H). ^**13**^**C{**^**1**^**H} NMR** (DMSO-*d*_6_, 75.45 MHz, δ): 175.2, 162.7, 153.4, 136.3, 135.8, 133.6, 131.7, 131.1, 130.6, 129.1, 125.8, 125.3, 123.9, 122.6, 122.3, 121.8, 121.1, 107.1, 67.0. **HRMS** (ESI) *m*/*z*: [M + H]^+^ calcd for C_23_H_17_BrN_3_O_2_^+^, 446.0499; found, 446.0494.

#### 1-(4-Methoxyphenyl)-4-(4-nitrophenyl)-1,5,10,10a-tetrahydropyrrolo[5,4-*b*][1,5]benzodiazepine-2,3-dione (**5s**)

Yellow solid; yield: 70%, 0.309 g; mp: 273.4–276.7 °C (decomposition); ^**1**^**H NMR** (DMSO-*d*_6_, 300.06 MHz, δ): 10.13 (s, 1H), 8.35 (d, *J* = 8.8 Hz, 2H), 7.80 (d, *J* = 7.2 Hz, 2H), 7.62 (d, *J* = 9.1 Hz, 2H), 7.32 (dd, *J* = 8.0, 1.5 Hz, 1H), 7.09 (d, *J* = 9.2 Hz, 2H), 7.04 (d, *J* = 1.7 Hz, 1H), 6.99 (dd, *J* = 7.9, 7.4, 1.5 Hz, 1H), 6.91 (dd, *J* = 8.5, 7.0, 1.8 Hz, 1H), 6.06 (s, 1H), 5.69 (s, 1H), 3.81 (s, 3H). ^**13**^**C{**^**1**^**H} NMR** (DMSO-*d*_6_, 75.45 MHz, δ): 175.9, 162.5, 157.3, 151.2, 148.53, 141.1, 136.3, 131.1, 130.5, 128.4, 125.5, 123.3, 122.6, 122.6, 121.6, 114.3, 107.0, 67.3, 55.4. **HRMS** (ESI) *m*/*z*: [M + H]^+^ calcd for C_24_H_19_N_4_O_5_^+^, 443.1350; found, 443.1338.

#### 1-(4-Methoxyphenyl)-4-phenyl-1,5,10,10a-tetrahydropyrrolo[5,4-*b*][1,5]benzodiazepine-2,3-dione (**5t**)

Yellow solid; yield: 80%, 0.318 g; mp: 262.9.0–265.6 °C (decomposition); ^**1**^**H NMR** (DMSO-*d*_6_, 300.06 MHz, δ): 9.89 (s, 1H), 7.63 (d, *J* = 9.1 Hz, 2H), 7.56–7.50 (m, 5H), 7.40 (dd, *J* = 8.0, 1.5 Hz, 1H), 7.08 (d, *J* = 9.1 Hz, 2H), 7.01 (dd, *J* = 8.0, 1.8 Hz, 1H), 6.95 (dd, *J* = 8.0, 7.4, 1.5 Hz, 1H), 6.87 (dd, *J* = 8.6, 6.9, 1.8 Hz, 1H), 6.06 (s, 1H), 5.67 (s, 1H), 3.81 (s, 3H). ^**13**^**C{**^**1**^**H} NMR** (DMSO-*d*_6_, 75.45 MHz, δ): 175.4, 162.6, 157.2, 154.5, 136.2, 134.5, 130.4, 130.3, 129.5, 128.6, 128.1, 125.1, 123.2, 122.6, 122.0, 121.6, 114.3, 106.9, 67.4, 55.4. **HRMS** (ESI) *m*/*z*: [M + H]^+^ calcd for C_24_H_20_N_3_O_3_^+^, 398.1499; found, 398.1491.

#### 1-(4-Methoxyphenyl)-4-(4-methoxyphenyl)-1,5,10,10a-tetrahydropyrrolo[5,4-*b*][1,5]benzodiazepine-2,3-dione (**5u**)

Yellow solid; yield: 70%, 0.299 g; mp: 285.6–287.9 °C (decomposition); ^**1**^**H NMR** (DMSO-*d*_6_, 300.06 MHz, δ): 9.77 (s, 1H), 7.66 (d, *J* = 9.1 Hz, 2H), 7.49 (d, *J* = 8.8 Hz, 2H), 7.41 (dd, *J* = 8.0, 1.5 Hz, 1H), 7.10–7.04 (*m*, 4H), 7.01–6.90 (m, 2H), 6.85 (dd, *J* = 8.0, 6.9, 1.8 Hz, 1H), 6.06 (s, 1H), 5.67 (s, 1H), 3.87 (s, 3H), 3.81 (s, 3H). ^**13**^**C{**^**1**^**H} NMR** (DMSO-*d*_6_, 75.45 MHz, δ): 175.1, 162.7, 161.3, 157.2, 154.5, 136.0, 131.5, 130.1, 128.6, 126.3, 124.9, 123.0, 122.6, 121.7, 121.3, 114.3, 113.3, 106.8, 67.2, 55.4, 55.3. **HRMS** (ESI) *m*/*z*: [M + H]^+^ calcd for C_25_H_22_N_3_O_4_^+^, 428.1605; found, 428.1598.

#### 1-(4-Methoxyphenyl)-4-(4-fluorophenyl)-1,5,10,10a-tetrahydropyrrolo[5,4-*b*][1,5]benzodiazepine-2,3-dione (**5v**)

Yellow solid; yield: 72%, 0.299 g; mp: 278.0–281.7 °C (decomposition); ^**1**^**H NMR** (DMSO-*d*_6_, 300.06 MHz, δ): 9.91 (s, 1H), 7.64 (d, *J* = 9.1 Hz, 2H), 7.91–756 (m, 2H), 7.38 (dd, *J* = 7.0, 1.7 Hz, 1H), 7.33 (d, *J* = 4.7 Hz, 2H), 7.09 (dd, *J* = 8.0, 1.8 Hz, 2H), 7.02 (dd, *J* = 8.0, 1.8 Hz, 1H), 6.96 (dd, *J* = 8.0, 6.9, 1.5 Hz, 1H), 6.88 (dd, *J* = 7.9, 6.9, 1.8 Hz, 1H), 6.06 (s, 1H), 5.69 (s, 1H), 3.82 (s, 3H). ^**13**^**C{**^**1**^**H} NMR** (DMSO-*d*_6_, 75.45 MHz, δ): 175.5, 163.5 (d, *J* = 245.0 Hz), 162.5, 157.2, 153.3, 136.2, 132.1 (d, *J* = 9.0 Hz) 130.7 (d, *J* = 3.0 Hz), 130.2, 128.5, 125.2, 123.1, 122.6, 122.0, 121.6, 115.0 (d, *J* = 21.8 Hz), 114.3, 107.0, 67.3, 55.4. **HRMS** (ESI) *m*/*z*: [M + H]^+^ calcd for C_24_H_19_FN_3_O_3_^+^, 416.1405; found, 416.1412.

#### 1-(4-Methoxyphenyl)-4-(4-chlorophenyl)-1,5,10,10a-tetrahydropyrrolo[5,4-*b*][1,5]benzodiazepine-2,3-dione (**5w**)

Yellow solid; yield: 71%, 0.306 g; mp: 300.0–302.9 °C (decomposition); ^**1**^**H NMR** (DMSO-*d*_6_, 300.06 MHz, δ): 9.94 (s, 1H), 7.63 (d, *J* = 9.1 Hz, 2H), 7.60–7.52 (m, 4H), 7.36 (dd, *J* = 8.0, 1.5 Hz, 1H), 7.09 (d, *J* = 9.1 Hz, 2H), 7.02 (dd, *J* = 8.0, 1.8 Hz, 1H), 6.97 (dd, *J* = 8.0, 7.5, 1.8 Hz, 1H), 6.89 (dd, *J* = 8.0, 7.5, 1.5 Hz, 1H), 6.04 (s, 1H), 5.69 (s, 1H), 3.81 (s, 3H). ^**13**^**C{**^**1**^**H} NMR** (DMSO-*d*_6_, 75.45 MHz, δ): 175.6, 162.5, 159.3, 157.3, 153.0 (2C), 136.2, 135.1, 133.3, 131.5, 130.3, 128.5, 128.1, 125.2, 123.2, 121.7, 121.4, 114.3, 107.0, 67.3, 55.4. **HRMS** (ESI) *m*/*z*: [M + H]^+^ calcd for C_24_H_19_ClN_3_O_3_^+^, 432.1109; found, 432.1095.

#### 1-(4-Methoxyphenyl)-4-(4-bromophenyl)-1,5,10,10a-tetrahydropyrrolo[5,4-*b*][1,5]benzodiazepine-2,3-dione (**5x**)

Orange solid; yield: 71%, 0.337 g; mp: 297.6–300.1 °C (decomposition); ^**1**^**H NMR** (DMSO-*d*_6_, 300.06 MHz, δ): 9.93 (s, 1H), 7.71 (d, *J* = 9.2 Hz, 2H), 7.62 (d, *J* = 4.8 Hz, 2H), 7.47 (d, *J* = 6.6 Hz, 2H), 7.35 (dd, *J* = 8.0, 1.5 Hz, 1H), 7.08 (d, *J* = 9.2 Hz, 2H), 7.02 (dd, *J* = 7.9, 1.8 Hz, 1H), 6.96 (dd, *J* = 8.0, 6.9, 1.5 Hz, 1H), 6.88 (dd, *J* = 8.6, 7.0, 1.8 Hz, 1H), 6.04 (s, 1H), 5.68 (s, 1H), 3.81 (s, 3H). ^**13**^**C{**^**1**^**H} NMR** (DMSO-*d*_6_, 75.45 MHz, δ): 175.6, 162.4, 157.3, 153.0, 136.2, 133.7, 131.0, 130.3, 128.5, 125.2, 123.9, 123.2, 122.6, 121.7, 121.4, 114.3, 107.0, 67.3, 55.4. **HRMS** (ESI) *m*/*z*: [M + H]^+^ calcd for C_24_H_19_BrN_3_O_3_^+^, 476.0604; found, 476.0580.

## Data Availability

The data underlying this study are available in the published article and its Supporting Information.

## Supporting Information Available

Experimental details, characterization of new compounds, and NMR spectra (PDF)
