# Supplementary material for: Cascade Cyclization/Annulation of β-Enamino Diketones and o-Phenylenediamine: A Strategy to Access Pyrrole-fused 1,5-Benzodiazepines
Source: J Org Chem. 2024 Nov 13;89(23):17720–8. doi: 10.1021/acs.joc.4c02483 (PMC11629378; doi:10.1021/acs.joc.4c02483)
Supplement: Supplementary file 1 — jo4c02483_si_001.pdf [file jo4c02483_si_001.pdf]

SUPPORTING INFORMATION

Cascade Cyclization/Annulation of β-Enamino Diketones and *o*-Phenylenediamine: A Strategy to Access Pyrrole-fused 1,5-Benzodiazepines

Julia Poletto,[†] Julia C. M. Willig,[†] Jeniffer N. A. Camargo,[†] Helio G. Bonacorso,[‡] Michael J. V. Silva,[§] and Fernanda A. Rosa *[,†]

[†] Laboratory for Synthesis of Heterocycles (SINTHET), Chemistry Department, State University of Maringá - UEM, Maringá, Paraná 87020-900, Brazil

[‡] Núcleo de Química de Heterociclos (NUQUIMHE), Chemistry Department, Federal University of Santa Maria - UFSM, Santa Maria, Rio Grande do Sul 97105-900, Brazil

[§] Federal Technological University of Paraná - UTFPR, Toledo, Paraná 85902-490, Brazil

*Email: farosa@uem.br

1. General Information……………………………………………..…………..……...………S2

2. Experimental Procedure

2.1. Procedure for the synthesis of β-enamino diketone **1a**………………………………………S2

2.2. Reaction of β-enamino diketone **1a** and *o*-phenylenediamine: Synthesis of **2a** ……………S2

2.3. Reaction of β-enamino diketone **1a** and *o*-phenylenediamine dihydrochloride: Synthesis of **3a**……………………………...………………………………………………………………S3

2.4 General procedure for the synthesis of β-enamino diketones **4**……………………………….S3

2.5 Reaction of β-enamino diketone **4** and *o*-phenylenediamine: General procedure for synthesis of **5a-x**………………………………………………………………………………………S3

3. Characterization data for the products **2a**, **3a**, and **5a-x**……………………………….….......S4

4. Copies of ^{1}H and ^{13}C{^{1}H} NMR spectra of **2a**, **3a**, and **5a-x**………………………..……….S11

5. References....…………………………………………………………………...…...…....S64

1. General Information

Reagents were used as obtained from commercial suppliers without further purification. Solvents were dried and purified according to recommended procedures.[1] The reactions were monitored by thin-layer chromatography using Merck TLC silica gel plates and visualized with UV light. All melting points were measured with the MQAPF-307 Microquímica apparatus using benzoic acid as the internal standard. ^{1}H NMR and ^{13}C{^{1}H} NMR experiments were run on Bruker Avance III HD apparatus operating at ^{1}H 300.06 MHz and ^{13}C 75.46 MHz or Bruker Avance III HD apparatus operating at ^{1}H 500.13 MHz and ^{13}C 125.77 MHz and all structural assignments were made with additional information from gHSQC and gHMBC experiments. Chemical shifts are reported in ppm using DMSO-d_6. ESI(+)-MS and tandem ESI(+)-MS/MS were acquired using a hybrid high-resolution and high accuracy microTof (Q-TOF) mass spectrometer (Bruker). For ESI(+)-MS, the energy for the collision-induced dissociations (CDI) was optimized for each component. For data acquisition and processing, the Q-TOF-control data analysis software (Bruker Scientific) was used.

2. Experimental Procedure

2.1 Procedure for the Synthesis of β-Enamino Diketone 1a

The β-enamino diketone **1a** was prepared following the literature method reported.[2] To a solution stirred solution of ethyl oxalyl chloride (2.0 mmol, 1.0 equiv, 0.273 g) in dry CH_2Cl_2 (8 mL), under nitrogen atmosphere, at 0 °C, a solution of β-enamino ketone (2.0 mmol, 1.0 equiv, 0.440 g) and pyridine (2.0 mmol, 1.0 equiv, 0.158 g) in dry CH_2Cl_2 (6 mL) was added dropwise over a period of 1 h. Then, the mixture was heated at 40 °C in an oil bath and stirred for another 15 h. After this, the organic layer was washed with a solution of H_2O–HCl (10:1; 1 × 20 mL), washed with H_2O (3 × 20 mL), and dried under vacuum to provide the product **1a** in 91% yield.

2.2 Reaction of β-Enamino Diketone 1a and *o*-Phenylenediamine: Synthesis of 2a

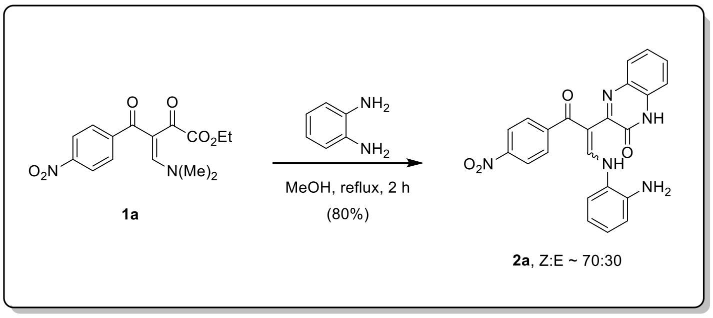

To a solution of β-enamino diketone **1a** (1.0 mmol, 1.0 equiv, 0.320 g) in methanol (15 mL), *o*-phenylenediamine (2.2 mmol, 2.2 equiv, 0.238 g) was added and the reaction was refluxed in an oil bath for 2 h (monitored by TLC). Then, the mixture was cooled to 0 °C and the solid was filtered, washed with cold methanol (10 mL) and dried under vacuum to provide product **2a**.

2.3 Reaction of β-Enamino Diketone 1a and *o*-Phenylenediamine Dihydrochloride: Synthesis of 3a

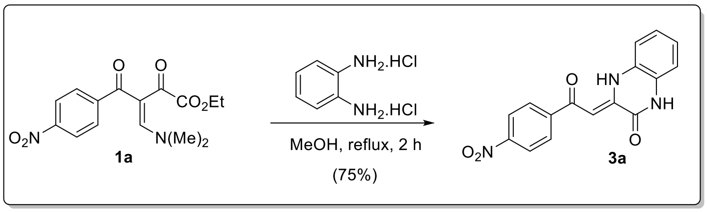

To a solution of β-enamino diketone **1a** (1.0 mmol, 1.0 equiv, 0.320 g) in methanol (15 mL), *o*-phenylenediamine dihydrochloride (1.2 mmol, 1.2 equiv, 0.217 g) was added and the reaction was refluxed in an oil bath for 2 h (monitored by TLC). Then, the mixture was cooled to 0 °C and the solid was filtered, washed with cold methanol (10 mL) and dried under vacuum to provide the product **3a**. The product **3a** is known in literature.[3]

2.4 General Procedure for the Synthesis of β-Enamino Diketones 4

β-enamino diketones **4** were prepared as per the method reported by us.[4-6] A mixture of compound **1** (1.0 mmol, 1.0 equiv) and corresponding amine hydrochloride salt (1.5 mmol, 1.5 equiv, $MeNH_2$.HCl: 0.101 g; $BnNH_2$.HCl: 0.215 g; $PhNH_2$.HCl: 0.194 g; 4-OMe$C_6H_4NH_2$.HCl: 0.239 g;) in ethanol (5 mL) was heated under reflux in an oil bath and stirred for 1 h. Then, the mixture was cooled to 0 °C and the solid was filtered, washed with cold ethanol (20 mL), and dried under vacuum. β-enamino diketones **4** were obtained according to this procedure in 70-90% yields.

2.5 Reaction of *β*-Enamino Diketone 4 and *o*-Phenylenediamine: General Procedure for Synthesis of 5a-x

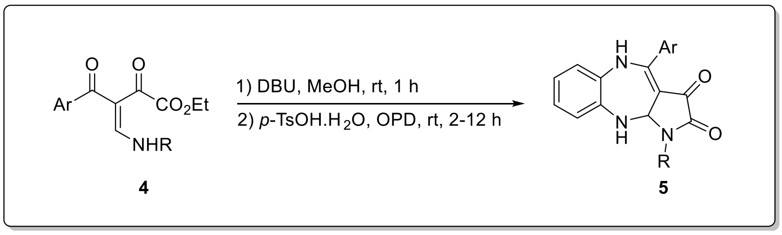

To a solution of β−enamino diketone **4** (1.0 mmol, 1.0 equiv) **4a** (Ar = 4−$NO_2C_6H_4$, R = Me): 0.306 g, **4b** (Ar = Ph, R = Me): 0.261 g, **4c** (Ar = 4−OMeC_6H_4, R = Me): 0.291 g, **4d** (Ar = 4−FC_6H_4, R = Me): 0.279 g, **4e** (Ar = 4−Cl C_6H_4, R = Me): 0.295 g, **4f** (Ar = 4−Br C_6H_4, R = Me): 0.339 g, **4g** (Ar = 4−$NO_2C_6H_4$, R = Bn): 0.382 g, **4h** (Ar = Ph, R = Bn): 0.337 g, **4i** (Ar = 4−OMeC_6H_4, R = Bn): 0.367 g, **4j** (Ar = 4−FC_6H_4, R = Bn): **4k** (Ar = 4−ClC_6H_4, R = Bn): 0.371 g, **4l** (Ar = 4−BrC_6H_4, R = Bn): 0.415 g, **4m** (Ar = 4−$NO_2C_6H_4$, R = Ph): 0.368 g, **4n** (Ar = Ph, R = Ph): 0.323 g, **4o** (Ar = 4−OMeC_6H_4, R = Ph): 0.353 g, 4p (Ar = 4−FC_6H_4, R = Ph): 0.341 g, **4q** (Ar = 4−Cl C_6H_4, R = Ph): 0.357 g, **4r** (Ar = 4−BrC_6H_4, R = Ph): 0.401 g, **4s** (Ar = 4−$NO_2C_6H_4$, R = 4−OMeC_6H_4): 0.398 g, **4t** (Ar = 4−Ph, R = 4−OMeC_6H_4): 0.353 g, **4u** (Ar = 4−OMeC_6H_4, R = 4−OMeC_6H_4): 0.383 g, **4v** (Ar = 4−FC_6H_4, R = 4−OMeC_6H_4): 0.371 g, **4w** (Ar = 4−ClC_6H_4, R = 4−OMeC_6H_4): 0.387 g, **4x** (Ar = 4−BrC_6H_4, R = 4−OMeC_6H_4): 0.431 g, in methanol (15 mL), DBU was added (1.2 mmol, 1.2 equiv, 0.183 g) and the mixture was stirred for 1 h at room temperature (monitored by TLC). After this time, *p*-TsOH.H_2O (2.2 mmol, 2.2 equiv, 0.418 g) and *o*-phenylenediamine (2.2 mmol,

2.2 equiv, 0.238 g) were successively added and the reaction was stirred at room temperature until completion (R = Me: 2 h; R = Bn and aryl: 12 h). The solid was filtered, washed with cold methanol (10 mL) and dried under vacuum to afford the respective products **5a-x**.

3. Characterization Data for the Products 2a, 3a, and 5a-x

3-(1-((2-Aminophenyl)amino)-3-(4-nitrophenyl)-3-oxoprop-1-en-2-yl)quinoxalin-2(1*H*)-one (2a):

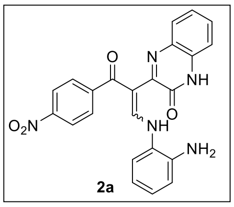

Orange solid; yield: 80%, 0.342 g; mp: 250.1-250.6 °C; mixture of *Z* and *E* - 30:70. **^{1}H NMR** (DMSO-d_6, 300.06 MHz): ***Z*** isomer: δ 12.15 (s, 1H), 11.63 (d, *J* = 14.0 Hz, 1H), 8.40 (d, *J* = 13.2 Hz, 1H), 8.16 (d, *J* = 8.9 Hz, 2H), 7.64 (d, *J* = 8.9 Hz, 2H), 7.57 (dd, *J* = 8.0, 1.4 Hz, 1H), 7.46-6.70 (m, 7H), 5.11 (s, 2H). ***E*** isomer: δ 12.20 (s, 1H), 11.59 (d, *J* = 13.6 Hz, 1H), 8.23 (d, *J* = 8.9 Hz, 2H), 8.04 (d, *J* = 13.1 Hz, 1H), 7.85 (d, *J* = 8.9 Hz, 2H), 7.81 (dd, *J* = 8.1, 1.5 Hz, 1H), 7.46-6.70 (m, 7H), 5.07 (s, 2H). **^{13}C{^{1}H} NMR** (DMSO-d_6, 125.77 MHz): ***Z*** and ***E*** isomers: δ 191.2, 190.8, 156.6, 155.7, 154.6, 154.0, 150.9, 148.5, 147.9, 147.8, 146.7, 145.5, 139.6, 139.2, 132.3, 131.8, 131.6, 130, 128.7, 128.5, 128.0, 127.9, 127.5, 127.3, 126.0, 125.2, 123.5, 123.4, 118.6, 117.4, 117.1, 115.1, 115.0, 107.4, 106.7, 89.5.
HRMS (ESI) m/z: $[M + Na]^+$ calcd for $C_{23}H_{17}N_5O_4Na^+$, 450.1178; found, 450.1183.

3-(2-(4-Nitrophenyl)-2-oxoethylidene)-3,4-dihydroquinoxalin-2(1*H*)-one (3a):

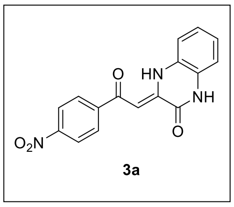

Orange solid; yield: 75%, 0.232 g; mp: 311-314 °C.
^{1}H NMR (DMSO-d_6, 300.06 MHz): δ 13.79 (*s*, 1H), 12.16 (*s*, 1H), 8.33 (*d*, *J* = 8.9 Hz, 2H), 8.20 (*d*, *J* = 8.9 Hz, 2H), 7.59-7.56 (*m*, 1H), 7.22-7.13 (*m*, 3H), 6.85 (*s*, 1H). **^{13}C{^{1}H} NMR (75.45 MHz, DMSO-*d₆*)**: δ 185.8, 155.5, 149.2, 146.8, 143.8, 128.4, 127.2, 124.9, 124.1, 117.2, 115.6, 89.6.

1-Methyl-4-(4-nitrophenyl)-1,5,10,10a-tetrahydropyrrolo[5,4-b][1,5]benzodiazepine-2,3-dione (5a):

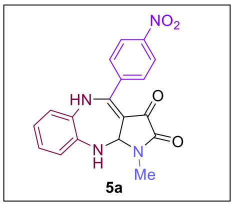

orange solid (0.318 g, 91% yield); mp: 275.0-278.2 °C (decomposition).
^{1}H NMR (300.06 MHz, DMSO-d_6): δ 9.94 (*s*, 1H), 8.32 (d, *J* = 8.8 Hz, 2H), 7.73 (d, *J* = 8.8 Hz, 2H), 7.27 (dd, *J* = 8.2, 1.5 Hz, 1H), 7.23 (dd, *J* = 8.2, 1.5 Hz, 1H), 7.02 (dd, J = 8.2, 7.2, 1.5 Hz, 1H), 6.87 (dd, J = 8.5, 7.1, 1.5 Hz, 1H), 6.22 (s, 1H), 5.27 (s, 1H), 3.13 (s, 3H). **^{13}C{^{1}H} NMR (75.45 MHz, DMSO-d_6)**: δ 177.0, 164.0, 151.1, 148.4, 141.3, 136.5, 131.1, 129.8, 125.3, 123.2, 122.5, 122.0, 121.5, 107.5, 69.3, 28.1.
HRMS (ESI) m/z: $[M + H]^+$ calcd for $C_{18}H_{15}N_4O_4^+$, 351.1088; found, 351.1091.

1-Methyl-4-phenyl-1,5,10,10a-tetrahydropyrrolo[5,4-b][1,5]benzodiazepine-2,3-dione (5b):

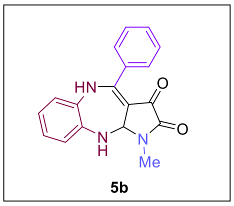

yellow solid (0.229 g, 75% yield); mp: 273.2-275.8 °C (decomposition).
^{1}H NMR (300.06 MHz, DMSO-d_6): δ 9.69 (s, 1H), 7.52-7.44 (m, 5H), 7.35 (dd, *J* = 8.1, 1.5 Hz, 1H), 7.19 (dd, *J* = 8.0, 1.5 Hz, 1H), 6.98 (dd, *J* = 8.1, 7.2, 1.4 Hz, 1H), 6.83 (dd, *J* = 8.5, 7.1, 1.5 Hz, 1H), 6.17 (s, 1H), 5.27 (s, 1H), 3.12 (s, 3H).
^{13}C{^{1}H} NMR (75.45 MHz, DMSO-d_6): δ 176.5, 164.2, 153.8, 136.3, 134.6, 130.2, 129.7, 129.4, 128.0, 124.8, 122.5, 121.5, 121.1, 107.2, 69.3, 28.0.

HRMS (ESI) m/z: [M + H]$^+$ calcd for $C_{18}H_{16}N_3O_2^+$, 306.1237; found, 306.1248.

1-Methyl-4-(4-methoxyphenyl)-1,5,10,10a-tetrahydropyrrolo[5,4-b][1,5]benzodiazepine-2,3-dione

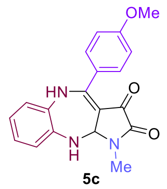

5c

(5c): orange solid (0.218 g, 65% yield); mp: 265.7-266.8 °C (decomposition).
^{1}H NMR (300.06 MHz, DMSO-d_6): δ 9.57 (s, 1H), 7.41 (d, J = 8.8 Hz, 2H), 7.36 (dd, J = 8.1, 1.5 Hz, 1H), 7.16 (dd, J = 8.1, 1.5 Hz, 1H), 7.01 (d, J = 8.8 Hz, 2H), 6.96 (dd, J = 8.1, 7.2, 1.4 Hz, 1H), 6.81 (dd, J = 8.5, 7.1, 1.5 Hz, 1H), 6.15 (s, 1H), 5.26 (s, 1H), 3.84 (s, 3H), 3.12 (s, 3H).
^{13}C{^{1}H} NMR (75.45 MHz, DMSO-d_6): δ 176.3, 164.4, 161.2, 153.9, 136.1, 131.4, 129.5, 126.4, 124.8, 122.5, 121.3, 120.9, 113.3, 107.1, 69.2, 55.4, 28.0.
HRMS (ESI) m/z: [M + H]$^+$ calcd for $C_{19}H_{18}N_3O_3^+$, 336.1343; found, 336.1340.

1-Methyl-4-(4-fluorophenyl)-1,5,10,10a-tetrahydropyrrolo[5,4-b][1,5]benzodiazepine-2,3-dione

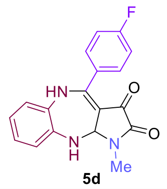

5d

(5d): orange solid (0.226 g, 70% yield); mp: 297.4-299.0 °C (decomposition).
^{1}H NMR (300.06 MHz, DMSO-d_6): δ 9.70 (s, 1H), 7.51-7.49 (m, 2H), 7.33-7.30 (m, 2H), 7.28 (d, J = 8.9, 1H), 7.19 (dd, J = 8.1, 1.5 Hz, 1H), 6.98 (dd, J = 8.1, 7.2, 1.5 Hz, 1H), 6.84 (dd, J = 8.5, 7.2, 1.5 Hz, 1H), 6.17 (s, 1H), 5.25 (s, 1H), 3.12 (s, 3H).
^{13}C{^{1}H} NMR (75.45 MHz, DMSO-d_6): δ 176.6, 164.2, 163.3 (d, J = 245 Hz), 152.6, 136.3, 132.0 (d, J = 9.0 Hz), 130.8 (d, J = 3.0 Hz), 129.6, 124.9, 122.5, 121.5, 121.1, 114.9 (d, J = 21.9 Hz), 107.3, 69.2, 28.0.
HRMS (ESI) m/z: [M + H]$^+$ calcd for $C_{18}H_{15}FN_3O_2^+$, 324.1143; found, 324.1152.

1-Methyl-4-(4-chlorophenyl)-1,5,10,10a-tetrahydropyrrolo[5,4-b][1,5]benzodiazepine-2,3-dione

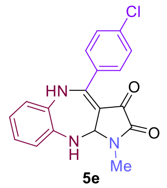

5e

(5e): red solid (0.271 g, 80% yield); mp: 276.9-279.0 °C (decomposition).
^{1}H NMR (300.06 MHz, DMSO-d_6): δ 9.74 (s, 1H), 7.54 (d, J = 8.6 Hz, 2H), 7.47 (d, J = 8.6 Hz, 2H), 7.31 (dd, J = 7.3, 1.5 Hz, 1H), 7.20 (dd, J = 8.1, 1.5 Hz, 1H), 6.99 (dd, J = 8.1, 7.2, 1.5 Hz, 1H), 6.85 (dd, J = 8.4, 7.1, 1.4 Hz, 1H), 6.18 (s, 1H), 5.25 (s, 1H), 3.12 (s, 3H).
^{13}C{^{1}H} NMR (75.45 MHz, DMSO-d_6): δ 176.7, 164.1, 152.3, 136.3, 134.9, 133.4, 131.4, 129.6, 128.0, 125.0, 122.5, 121.6, 121.2 , 107.3, 69.2, 28.0.
HRMS (ESI) m/z: [M + H]$^+$ calcd for $C_{18}H_{15}ClN_3O_2^+$, 340.0847; found, 340.0842.

1-Methyl-4-(4-bromophenyl)-1,5,10,10a-tetrahydropyrrolo[5,4-b][1,5]benzodiazepine-2,3-dione

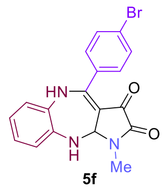

5f

(5f): red solid (0.299 g, 78% yield); mp: 276.5-279.9 °C (decomposition).
^{1}H NMR (300.06 MHz, DMSO-d_6): δ 9.74 (s, 1H), 7.68 (d, J = 8,4 Hz, 2H), 7.40 (d, J = 8,4 Hz, 2H), 7.30 (dd, J = 8.1, 1.4 Hz, 1H), 7.20 (dd, J = 8.1, 1.5 Hz, 1H), 6.99 (dd, J = 8.4, 7.1, 1.5 Hz, 1H), 6.84 (dd, J = 8.4, 7.1, 1.4 Hz, 1H), 6.18 (s, 1H), 5.24 (s, 1H), 3.12 (s, 3H).
^{13}C{^{1}H} NMR (75.45 MHz, DMSO-d_6): δ 176.7, 164.1, 152.4, 136.3, 133.8, 131.6, 131.0, 129.6, 125.0, 123.7, 122.5, 121.6, 121.2, 107.3, 69.2, 28.0.
HRMS (ESI) m/z: [M + H]$^+$ calcd for $C_{18}H_{15}BrN_3O_2^+$, 384.0342; found, 384.0350.

1-Benzyl-4-(4-nitrophenyl)-1,5,10,10a-tetrahydropyrrolo[5,4-b][1,5]benzodiazepine-2,3-dione (5g): red solid (0.383 g, 90% yield); mp: 281.9-283.4 °C (decomposition).

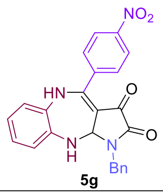

5g

^{1}H NMR (300.06 MHz, DMSO-*d*$_6$): δ 9.99 (s, 1H), 8.30 (d, *J* = 8.8 Hz, 2H), 7.75 (d, *J* = 8.8 Hz, 2H), 7.39-7.29 (m, 5H), 7.25-7.21 (m, 2H), 7.02 (dd, *J* = 8.3, 7.2, 1.4 Hz, 1H), 6.87 (dd, *J* = 8.5, 7.2, 1.4 Hz, 1H), 6.23 (s, 1H), 5.16 (d, *J* =14.9 Hz, 1H), 4.92 (s, 1H), 4.70 (d, *J* = 14.9 Hz, 1H).

^{13}C{^{1}H} NMR (75.45 MHz, DMSO-*d*$_6$): δ 176.5, 163.5, 151.6, 148.3, 141.2, 136.3, 135.6, 131.0, 130.4, 128.9, 128.5, 127.8, 125.4, 123.0, 122.6, 122.2, 121.9, 107.3, 66.4, 42.8.

HRMS (ESI) m/z: [M + H]$^+$ calcd for $C_{24}H_{19}N_4O_4^+$, 427.1401; found, 427.1405.

1-Benzyl-4-phenyl-1,5,10,10a-tetrahydropyrrolo[5,4-b][1,5]benzodiazepine-2,3-dione (5h): orange solid (0.263 g, 69% yield); mp: 277.0-279.5 °C (decomposition).

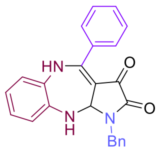

5h

^{1}H NMR (300.06 MHz, DMSO-*d*$_6$): δ 9.74 (s, 1H), 7.56-7.44 (m, 5H), 7.38 (dt, *J* = 6.9, 1.5, 1.5 Hz, 1H), 7.34-7.29 (m, 5H), 7.18 (dd, *J* = 8.0 Hz, 1.5 Hz, 1H), 6.98 (dd, *J* = 8.0, 7.2, 1.4 Hz, 1H,), 6.83 (dd, *J* = 8.0, 7.2, 1.4 Hz, 1H), 6.17 (s, 1H), 5.15 (d, *J* = 14.9 Hz, 1H), 4.92 (s, 1H), 4.68 (d, *J* = 14.9 Hz, 1H).

^{13}C{^{1}H} NMR (75.45 MHz, DMSO-*d*$_6$): δ 176.1, 163.9, 154.3, 136.2, 135.7, 134.5, 130.3, 130.2, 129.4, 128.9, 128.4, 127.9, 127.8, 125.1, 122.6, 121.7, 121.6, 107.0, 66.4, 42.8.

HRMS (ESI) m/z: [M + H]$^+$ calcd for $C_{24}H_{20}N_3O_2^+$, 382.1550; found, 382.1535.

1-Benzyl-4-(4-methoxyphenyl)-1,5,10,10a-tetrahydropyrrolo[5,4-b][1,5]benzodiazepine-2,3-dione (5i): orange solid (0.329 g, 80% yield); mp: 271.6-274.0 °C (decomposition).

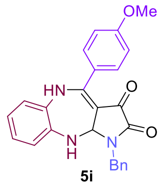

5i

^{1}H NMR (300.06 MHz, DMSO-*d*$_6$): δ 9.62 (s, 1H), 7.41 (d, *J* = 8.7 Hz, 2H), 7.36-7.29 (m, 6H), 7.15 (dd, *J* = 8,0, 1.5 Hz, 1H), 7.00 (d, *J* = 8.7 Hz, 2H), 6.97-6.94 (m, 1H), 6.81 (dd, *J* = 7.6, 7.0, 1.5 Hz, 1H), 6.15 (s, 1H), 5.14 (d, *J* = 15.0 Hz, 1H), 4.92 (s, 1H), 4.66 (d, *J* = 15.0 Hz, 1H), 3.83 (s, 3H).

^{13}C{^{1}H} NMR (75.45 MHz DMSO-*d*$_6$): δ 175.9, 164.0, 161.2, 154.4, 136.0, 135.8, 131.4, 130.0, 128.9, 128.4, 127.8, 126.3, 124.9, 122.4, 121.5, 121.3, 113.2, 106.9, 66.3, 55.4, 42.8.

HRMS (ESI) m/z: [M + H]$^+$ calcd for $C_{25}H_{22}N_3O_3^+$, 412.1656; found, 412.1640.

1-Benzyl-4-(4-fluorophenyl)-1,5,10,10a-tetrahydropyrrolo[5,4-b][1,5]benzodiazepine-2,3-dione (5j): red solid (0.351 g, 88% yield); mp: 305.1-308.2 °C (decomposition).

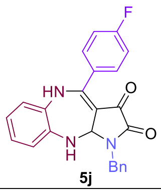

5j

^{1}H NMR (500.13 MHz, DMSO-*d*$_6$): δ 9.76 (s, 1H), 7.53-7.50 (m, 2H), 7.37-7.27 (m, 8H), 7.19 (dd, *J* = 8.2, 1.5 Hz, 1H), 6.99 (dd, *J* = 8.3, 7.2, 1.4 Hz, 1H), 6.84 (dd, *J* = 8.5, 7.2, 1.45 Hz, 1H), 6.17 (s, 1H), 5.15 (d, *J* = 14.9 Hz, 1H), 4.92 (s, 1H), 4.68 (d, *J* =14.9 Hz, 1H).

^{13}C{^{1}H} NMR (125.77 MHz, DMSO-*d*$_6$): δ 176.0, 163.6, 163.3 (*d*, *J* = 245.0 Hz), 153.0, 136.0, 135.5, 131.8 (*d*, *J* = 8.7 Hz), 130.6 (*d*, *J* = 3.1 Hz), 130.0, 128.7, 128.2, 127.6, 125.0, 122.4, 121.6, 121.4, 114.7 (*d*, *J* = 21.8 Hz), 106.8, 66.1, 42.5.

HRMS (ESI) m/z: [M + H]$^+$ calcd for $C_{24}H_{19}FN_3O_2^+$, 400.1456; found, 400.1458.

1-Benzyl-4-(4-chlorophenyl)-1,5,10,10a-tetrahydropyrrolo[5,4-b][1,5]benzodiazepine-2,3-dione (5k):

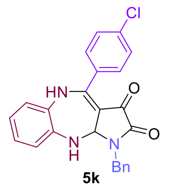

5k

red solid (0.369 g, 89% yield); mp: 308.2-309.8 °C (decomposition).
^{1}H NMR (500.13 MHz, DMSO-d_6): δ 9.79 (s, 1H), 7.52 (d, J = 8.6 Hz, 2H), 7.47 (d, J = 8.6 Hz, 2H), 7.36-7.32 (m, 5H), 7.27 (dd, J = 8.2, 1.5 Hz, 1H), 7.19 (dd, J = 8.1, 1.5 Hz, 1H), 6.99 (dd, J = 8.2, 7.3, 1.4 Hz, 1H), 6.84 (dd, J = 8.4, 7.2, 1.5 Hz, 1H), 6.18 (s, 1H), 5.14 (d, J =14.9 Hz, 1H), 4.91 (*s*, 1H), 4.68 (*d*, J = 14.9 Hz, 1H). **^{13}C{^{1}H} NMR (125.77 MHz, DMSO-d_6)**: δ 176.2, 163.7, 152.9, 136.1, 135.7, 134.9, 133.3, 131.4, 130.2, 128.9, 128.4, 128.0, 127.8, 125.2, 122.5, 121.8, 121.7, 107.1, 66.4, 42.8.

HRMS (ESI) m/z: [M + H]$^+$ calcd for $C_{24}H_{19}ClN_3O_2^+$, 416.1160; found, 416.1154.

1-Benzyl-4-(4-bromophenyl)-1,5,10,10a-tetrahydropyrrolo[5,4-b][1,5]benzodiazepine-2,3-dione (5l):

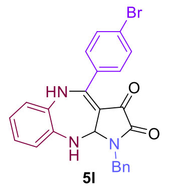

5l

orange solid (0.418 g, 91% yield); mp: 302.6-304.7 °C (decomposition).
^{1}H NMR (300.06 MHz, DMSO-d_6): δ 9.79 (s, 1H), 7.66 (d, J = 8.5 Hz, 2H), 7.40 (d, J = 8.5 Hz, 2H), 7.34-7.32 (m, 5H), 7.27 (dd, J = 8.2, 1.5 Hz, 1H), 7.19 (dd, J = 8.1, 1.5 Hz, 1H), 6.99 (dd, J = 8.2, 7.3, 1.4 Hz, 1H), 6.84 (dd, J = 8.2, 7.2, 1.3 Hz, 1H), 6.18 (s, 1H), 5.15 (d, J =14.9 Hz, 1H), 4.90 (s, 1H), 4.68 (d, J =14.9 Hz, 1H). **^{13}C{^{1}H} NMR (75.45 MHz, DMSO-d_6)**: δ 176.0, 163.5, 152.6, 135.9, 135.4, 133.4, 131.3, 130.6, 130.0, 128.6, 128.1, 127.5, 124.9, 123.4, 122.3, 121.6, 121.4, 106.8, 66.1, 42.5.

HRMS (ESI) m/z: [M + H]$^+$ calcd for $C_{24}H_{19}BrN_3O_2^+$, 460.0655; found, 460.0657.

1-Phenyl-4-(4-nitrophenyl)-1,5,10,10a-tetrahydropyrrolo[5,4-b][1,5]benzodiazepine-2,3-dione (5m):

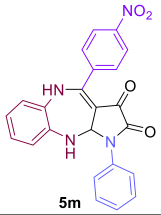

5m

orange solid (0.321 g, 78% yield); mp: 250.3-253.6 °C (decomposition).
^{1}H NMR (300.06 MHz, DMSO-d_6): δ 10.18 (s, 1H), 8.36 (d, J = 8.8 Hz, 2H), 7.81 (d, J = 8.8 Hz, 2H), 7.72 (d, J = 7.6 Hz, 2H), 7.55-7.52 (m, 2H), 7.34 (d, J = 7.6, 1.4 Hz, 2H), 7.06 (dd, J = 8.0, 2.0 Hz, 1H), 7.00 (dd, J = 7.9, 1.4 Hz, 1H), 6.93 (dd, J = 8.6, 6.8, 1.9 Hz, 1H), 6.12 (s, 1H), 5.87 (s, 1H).

^{13}C{^{1}H} NMR (75.45 MHz, DMSO-d_6): δ 175.5, 162.5, 151.9, 148.9, 141.12, 136.4, 135.7, 131.1, 130.7, 129.1, 125.9, 125.6, 123.2, 122.7, 122.1, 121.3, 107.3, 67.1.

HRMS (ESI) m/z: [M + H]$^+$ calcd for $C_{23}H_{17}N_4O_4^+$, 413.1244; found, 413.1236.

1-Phenyl-4-phenyl-1,5,10,10a-tetrahydropyrrolo[5,4-b][1,5]benzodiazepine-2,3-dione (5n): orange

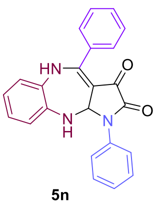

5n

solid (0.253 g, 69% yield); mp: 280.0-283.4 °C (decomposition).
^{1}H NMR (300.06 MHz, DMSO-d_6): δ 9.94 (s, 1H), 7.74 (dd, J = 8.7, 1.2 Hz, 2H), 7.58-7.54 (m, 2H), 7.52-7.49 (m, 5H), 7.41 (dd, J = 8.0, 1.4 Hz, 1H), 7.34-7.29 (m, 1H), 7.02 (dd, J = 8.0, 2.0 Hz, 1H), 6.96 (dd, J = 7.9, 1.4 Hz, 1H), 6.88 (dd, J = 7.9, 6.7, 2.0 Hz, 1H), 6.13 (s, 1H), 5.82 (s, 1H).

^{13}C{^{1}H} NMR (75.45 MHz, DMSO-d_6): δ 175.0, 162.8, 154.8, 136.3, 135.8, 134.4, 130.5, 130.4, 129.5, 129.0, 128.0, 125.7, 125.2, 122.7, 122.1, 121.6, 121.1, 107.0, 67.0.

HRMS (ESI) m/z: [M + H]$^+$ calcd for $C_{23}H_{18}N_3O_2^+$, 368.1394; found, 368.1404.

1-Phenyl-4-(4-methoxyphenyl)-1,5,10,10a-tetrahydropyrrolo[5,4-b][1,5]benzodiazepine-2,3-dione

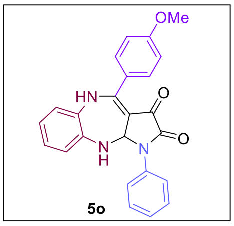

5o

(5o): orange solid (0.282 g, 71% yield); mp: 264.4-266.5 °C (decomposition).

^{1}H NMR (300.06 MHz, DMSO-*d*$_6$): δ 9.82 (s, 1H), 7.75 (d, *J* = 7.5 Hz, 2H), 7.54-7.46 (m, 4H), 7.42 (d, *J* = 7.5 Hz, 1H), 7.31 (t, *J* = 7.4 Hz, 1H), 7.04 (d, *J* = 8.7 Hz, 2H), 6.99-6.92 (m, 2H), 6.86 (dd, *J* = 8.5, 6.4, 2.2 Hz, 1H), 6.11 (s, 1H), 5.78 (s, 1H), 3.85 (s, 3H).

^{13}C{^{1}H} NMR (75.45 MHz, DMSO-*d*$_6$): δ 174.9, 163.2, 161.5, 155.0, 136.1, 136.0, 131.6, 130.3, 129.2, 126.3, 125.9, 125.2, 122.8, 121.8, 121.5, 121.2, 113.5, 107.3, 67.1, 55.5.

HRMS (ESI) m/z: [M + H]$^+$ calcd for $C_{24}H_{20}N_3O_3^+$, 398.1499; found, 398.1486.

1-Phenyl-4-(4-fluorophenyl)-1,5,10,10a-tetrahydropyrrolo[5,4-b][1,5]benzodiazepine-2,3-dione (5p):

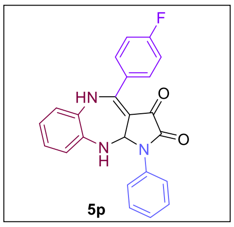

5p

orange solid (0.273 g, 71% yield); mp: 296.7-300.1 °C (decomposition).

^{1}H NMR (300.06 MHz, DMSO-*d*$_6$): δ 9.96 (s, 1H), 7.74 (dd, *J* = 8.8, 1.2 Hz, 2H), 7.61-7.56 (m, 2H), 7.54-7.49 (m, 2H), 7.40-7.29 (m, 4H), 7.03-6.94 (m, 2H), 6.89 (dd, *J* = 8.6, 6.8, 2.0 Hz, 1H), 6.12 (s, 1H), 5.83 (s, 1H).

^{13}C{^{1}H} NMR (75.45 MHz, DMSO-*d*$_6$): δ 175.1, 163.5 (*d*, *J* = 245.3 Hz), 162.8, 154.6, 153.7, 136.2, 135.8, 132.1 (*d*, *J* = 9.2 Hz), 130.7 (*d*, *J* = 3.0 Hz), 130.5, 129.1, 125.8, 125.3, 122.7, 122.2, 121.7, 121.2, 115.0 (*d*, *J* = 22.0 Hz), 107.1, 67.0.

HRMS (ESI) m/z: [M + H]$^+$ calcd for $C_{23}H_{17}FN_3O_2^+$, 386.1299; found, 386.1299.

1-Phenyl-4-(4-chlorophenyl)-1,5,10,10a-tetrahydropyrrolo[5,4-b][1,5]benzodiazepine-2,3-dione

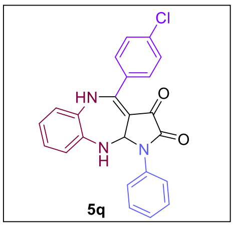

5q

(5q): orange solid (0.293 g, 73% yield); mp: 296.7-299.6 °C (decomposition).

^{1}H NMR (300.06 MHz, DMSO-*d*$_6$): δ 9.99 (s, 1H), 7.73 (d, *J* = 7.6 Hz, 2H), 7.56 (d, *J* = 4.3 Hz, 4H), 7.51 (d, *J* = 8.5 Hz, 2H), 7.37 (dd, *J* = 8.0, 1.4 Hz, 1H), 7.34-7.29 (m, 1H), 7.02 (dd, *J* = 8.1, 2.2 Hz, 1H), 6.97 (dd, *J* = 7.9, 1.4 Hz, 1H), 6.90 (dd, *J* = 8.5, 6.8, 1.9 Hz, 1H), 6.11 (s, 1H), 5.83 (s, 1H).

^{13}C{^{1}H} NMR (75.45 MHz, DMSO-*d*$_6$): δ 175.2, 162.7, 153.3, 136.3, 135.1, 133.2, 131.5, 130.5, 129.1, 128.1, 125.8, 125.8, 125.3, 122.6, 122.3, 121.8, 121.2, 107.1, 67.0.

HRMS (ESI) m/z: [M + H]$^+$ calcd for $C_{23}H_{17}ClN_3O_2^+$, 402.1004; found, 402.1002.

1-Phenyl-4-(4-bromophenyl)-1,5,10,10a-tetrahydropyrrolo[5,4-b][1,5]benzodiazepine-2,3-dione

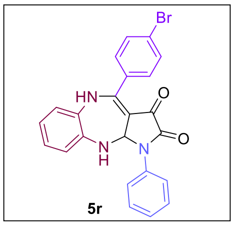

5r

(5r): orange solid (0.320 g, 72% yield); mp: 290.1-292.5 °C (decomposition).

^{1}H NMR (300.06 MHz, DMSO-*d*$_6$): δ 9.99 (s, 1H), 7.73-7.70 (m, 4H), 7.53 (d, *J* = 7.8 Hz, 2H), 7.47 (d, *J* = 8.5 Hz, 2H), 7.36 (dd, *J* = 8.0, 1.4 Hz, 1H), 7.30 (d, *J* = 9.1 Hz, 1H), 7.01-6.94 (m, 2H), 6.88 (dd, *J* = 8.6, 6.7, 1.9 Hz, 1H), 6.10 (s, 1H), 5.83 (s, 1H).

^{13}C{^{1}H} NMR (75.45 MHz, DMSO-*d*$_6$): δ 175.2, 162.7, 153.4, 136.3, 135.8, 133.6, 131.7, 131.1, 130.6, 129.1, 125.8, 125.3, 123.9, 122.6, 122.3, 121.8, 121.1, 107.1, 67.0.

HRMS (ESI) m/z: [M + H]$^+$ calcd for $C_{23}H_{17}BrN_3O_2^+$, 446.0499; found, 446.0494.

1-(4-Methoxyphenyl)-4-(4-nitrophenyl)-1,5,10,10a-tetrahydropyrrolo[5,4-b][1,5]benzodiazepine-2,3-dione (5s): yellow solid (0.309 g, 70% yield); mp: 273.4-276.7 °C (decomposition).

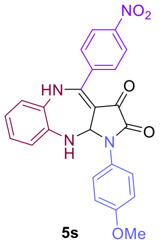

5s

^{1}H NMR (300.06 MHz, DMSO-*d6*): δ 10.13 (s, 1H), 8.35 (d, *J* = 8.8 Hz, 2H), 7.80 (d, *J* = 7.2 Hz, 2H), 7.62 (d, *J* = 9.1 Hz, 2H), 7.32 (dd, *J* = 8.0, 1.5 Hz, 1H), 7.09 (d, *J* = 9.2 Hz, 2H), 7.04 (d, *J* = 1.7 Hz, 1H), 6.99 (dd, *J* = 7.9, 7.4, 1.5 Hz, 1H), 6.91 (dd, *J* = 8.5, 7.0, 1.8 Hz, 1H), 6.06 (s, 1H), 5.69 (s, 1H), 3.81 (s, 3H).

^{13}C{^{1}H} NMR (75.45 MHz, DMSO-*d6*): δ 175.9, 162.5, 157.3, 151.2, 148.53, 141.1, 136.3, 131.1, 130.5, 128.4, 125.5, 123.3, 122.6, 122.6, 121.6, 114.3, 107.0, 67.3, 55.4.

HRMS (ESI) m/z: $[M + H]^+$ calcd for $C_{24}H_{19}N_4O_5^+$, 443.1350; found, 443.1338.

1-(4-Methoxyphenyl)-4-phenyl-1,5,10,10a-tetrahydropyrrolo[5,4-b][1,5]benzodiazepine-2,3-dione (5t): yellow solid (0.318 g, 80% yield); mp: 262.9.0-265.6 °C (decomposition).

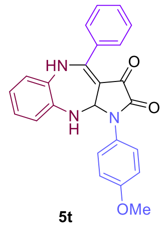

5t

^{1}H NMR (300.06 MHz, DMSO-*d6*): δ 9.89 (s, 1H), 7.63 (d, *J* = 9.1 Hz, 2H), 7.56-7.50 (m, 5H), 7.40 (dd, *J* = 8.0, 1.5 Hz, 1H), 7.08 (d, *J* = 9.1 Hz, 2H), 7.01 (dd, *J* = 8.0, 1.8 Hz, 1H), 6.95 (dd, *J* = 8.0, 7.4, 1.5 Hz, 1H), 6.87 (dd, *J* = 8.6, 6.9, 1.8 Hz, 1H), 6.06 (s, 1H), 5.67 (s, 1H), 3.81 (s, 3H).

^{13}C{^{1}H} NMR (75.45 MHz, DMSO-*d6*): δ 175.4, 162.6, 157.2, 154.5, 136.2, 134.5, 130.4, 130.3, 129.5, 128.6, 128.1, 125.1, 123.2, 122.6, 122.0, 121.6, 114.3, 106.9, 67.4, 55.4.

HRMS (ESI) m/z: $[M + H]^+$ calcd for $C_{24}H_{20}N_3O_3^+$, 398.1499; found, 398.1491.

1-(4-Methoxyphenyl)-4-(4-methoxyphenyl)-1,5,10,10a-tetrahydropyrrolo[5,4-b][1,5]benzodiazepine-2,3-dione (5u): yellow solid (0.299 g, 70% yield); mp: 285.6-287.9 °C (decomposition).

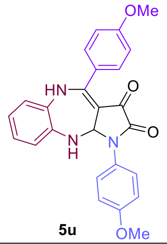

5u

^{1}H NMR (300.06 MHz, DMSO-*d6*): δ 9.77 (s, 1H), 7.66 (d, *J* = 9.1 Hz, 2H), 7.49 (d, *J* = 8.8 Hz, 2H), 7.41 (dd, *J* = 8.0, 1.5 Hz, 1H), 7.10-7.04 (m, 4H), 7.01-6.90 (m, 2H), 6.85 (dd, *J* = 8.0, 6.9, 1.8 Hz, 1H), 6.06 (s, 1H), 5.67 (s, 1H), 3.87 (s, 3H), 3.81 (s, 3H).

^{13}C{^{1}H} NMR (75.45 MHz, DMSO-*d6*): δ 175.1, 162.7, 161.3, 157.2, 154.5, 136.0, 131.5, 130.1, 128.6, 126.3, 124.9, 123.0, 122.6, 121.7, 121.3, 114.3, 113.3, 106.8, 67.2, 55.4, 55.3.

HRMS (ESI) m/z: $[M + H]^+$ calcd for $C_{25}H_{22}N_3O_4^+$, 428.1605; found, 428.1598.

1-(4-Methoxyphenyl)-4-(4-fluorophenyl)-1,5,10,10a-tetrahydropyrrolo[5,4-b][1,5]benzodiazepine-2,3-dione (5v): yellow solid (0.299 g, 72% yield); mp: 278.0-281.7 °C (decomposition).

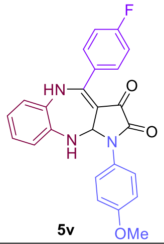

5v

^{1}H NMR (300.06 MHz, DMSO-*d6*): δ 9.91 (s, 1H), 7.64 (d, *J* = 9.1 Hz, 2H), 7.91-756 (m, 2H), 7.38 (dd, *J* = 7.0, 1.7 Hz, 1H), 7.33 (d, *J* = 4.7 Hz, 2H), 7.09 (dd, *J* = 8.0, 1.8 Hz, 2H), 7.02 (dd, *J* = 8.0, 1.8 Hz, 1H), 6.96 (dd, *J* = 8.0, 6.9, 1.5

Hz, 1H), 6.88 (dd, J = 7.9, 6.9, 1.8 Hz, 1H), 6.06 (s, 1H), 5.69 (s, 1H), 3.82 (s, 3H).

^{13}C{^{1}H} NMR (75.45 MHz, DMSO-*d*$_6$): δ 175.5, 163.5 (*d*, J = 245.0 Hz), 162.5, 157.2, 153.3, 136.2, 132.1 (*d*, J = 9.0 Hz) 130.7 (*d*, J = 3.0 Hz), 130.2, 128.5, 125.2, 123.1, 122.6, 122.0, 121.6, 115.0 (*d*, J = 21.8 Hz), 114.3, 107.0, 67.3, 55.4.

HRMS (ESI) m/z: [M + H]$^+$ calcd for $C_{24}H_{19}FN_3O_3^+$, 416.1405; found, 416.1412.

1-(4-Methoxyphenyl)-4-(4-chlorophenyl)-1,5,10,10a-tetrahydropyrrolo[5,4-b][1,5]benzodiazepine-2,3-dione (5w): yellow solid (0.306 g, 71% yield); mp: 300.0-302.9 °C (decomposition).

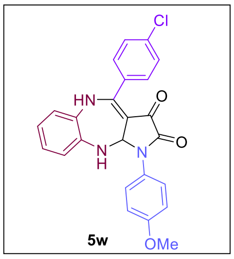

^{1}H NMR (300.06 MHz, DMSO-*d*$_6$): δ 9.94 (s, 1H), 7.63 (d, J = 9.1 Hz, 2H), 7.60-7.52 (m, 4H), 7.36 (dd, J = 8.0, 1.5 Hz, 1H), 7.09 (d, J = 9.1 Hz, 2H), 7.02 (dd, J = 8.0, 1.8 Hz, 1H), 6.97 (dd, J = 8.0, 7.5, 1.8 Hz, 1H), 6.89 (dd, J = 8.0, 7.5, 1.5 Hz, 1H), 6.04 (s, 1H), 5.69 (s, 1H), 3.81 (s, 3H).

^{13}C{^{1}H} NMR (75.45 MHz, DMSO-*d*$_6$): δ 175.6, 162.5, 159.3, 157.3, 153.0 (2C), 136.2, 135.1, 133.3, 131.5, 130.3, 128.5, 128.1, 125.2, 123.2, 121.7, 121.4, 114.3, 107.0, 67.3, 55.4.

HRMS (ESI) m/z: [M + H]$^+$ calcd for $C_{24}H_{19}ClN_3O_3^+$, 432.1109; found, 432.1095.

1-(4-Methoxyphenyl)-4-(4-bromophenyl)-1,5,10,10a-tetrahydropyrrolo[5,4-b][1,5]benzodiazepine-2,3-dione (5x): orange solid (0.337 g, 71% yield); mp: 297.6-300.1 °C (decomposition).

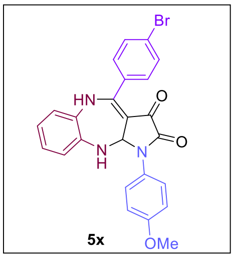

^{1}H NMR (300.06 MHz, DMSO-*d*$_6$): δ 9.93 (s, 1H), 7.71 (d, J = 9.2 Hz, 2H), 7.62 (d, J = 4.8 Hz, 2H), 7.47 (d, J = 6.6 Hz, 2H), 7.35 (dd, J = 8.0, 1.5 Hz, 1H), 7.08 (d, J = 9.2 Hz, 2H), 7.02 (dd, J = 7.9, 1.8 Hz, 1H), 6.96 (dd, J = 8.0, 6.9, 1.5 Hz, 1H), 6.88 (dd, J = 8.6, 7.0, 1.8 Hz, 1H), 6.04 (s, 1H), 5.68 (s, 1H), 3.81 (s, 3H).

^{13}C{^{1}H} NMR (75.45 MHz, DMSO-*d*$_6$): δ 175.6, 162.4, 157.3, 153.0, 136.2, 133.7, 131.0, 130.3, 128.5, 125.2, 123.9, 123.2, 122.6, 121.7, 121.4, 114.3, 107.0, 67.3, 55.4.

HRMS (ESI) m/z: [M + H]$^+$ calcd for $C_{24}H_{19}BrN_3O_3^+$, 476.0604; found, 476.0580.

4. Copies of ^{1}H and ^{13}C{^{1}H} NMR spectra of 2a, 3a, and 5a-x

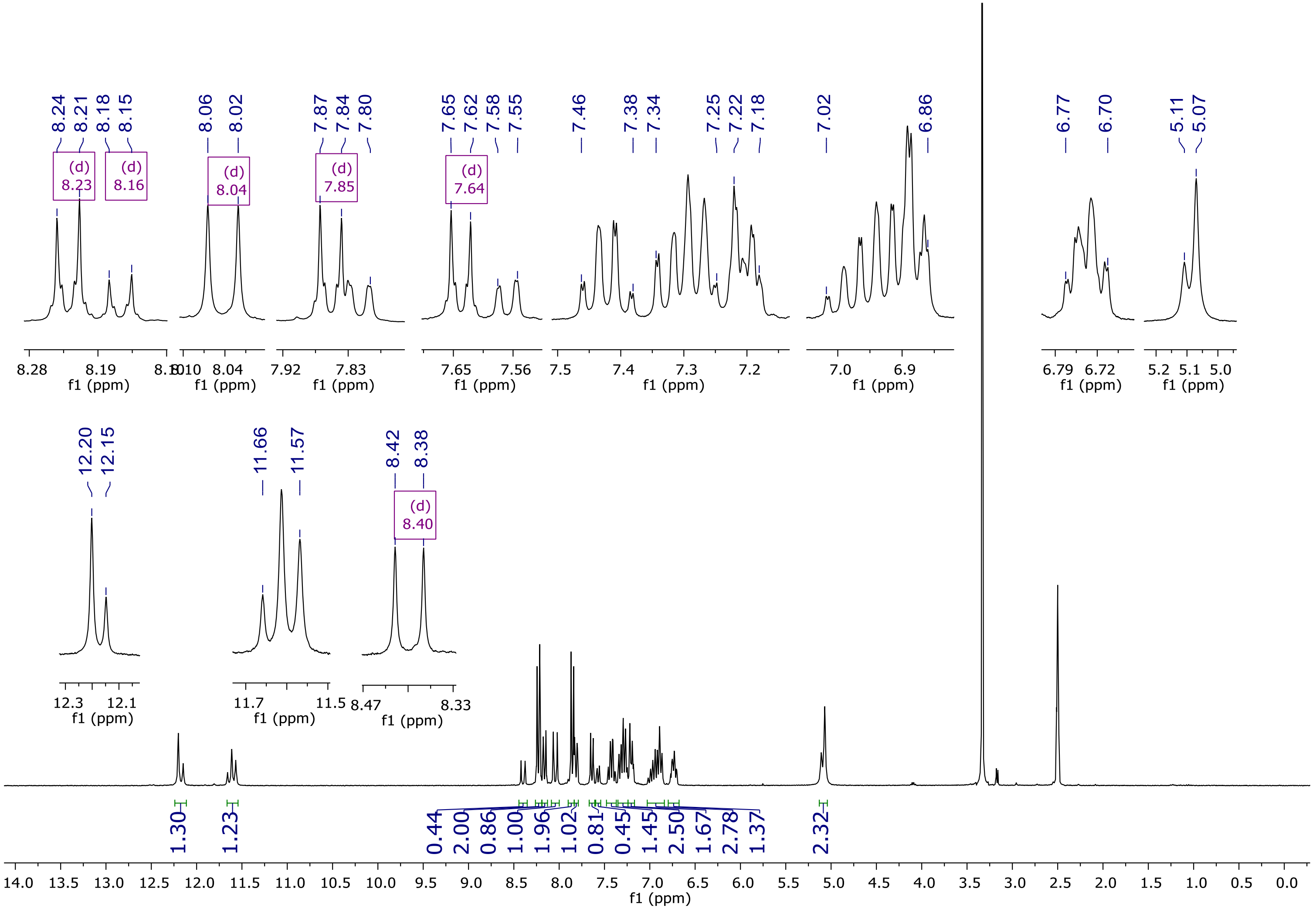

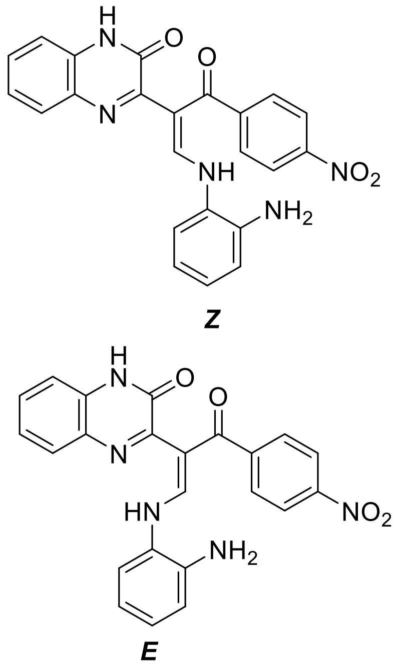

2a (***Z*:*E*** ~ 30:70)

Figure S1. ^{1}H NMR spectrum of *Z* and *E* isomers mixture of **2a** (DMSO-d_6, 300.06 MHz)

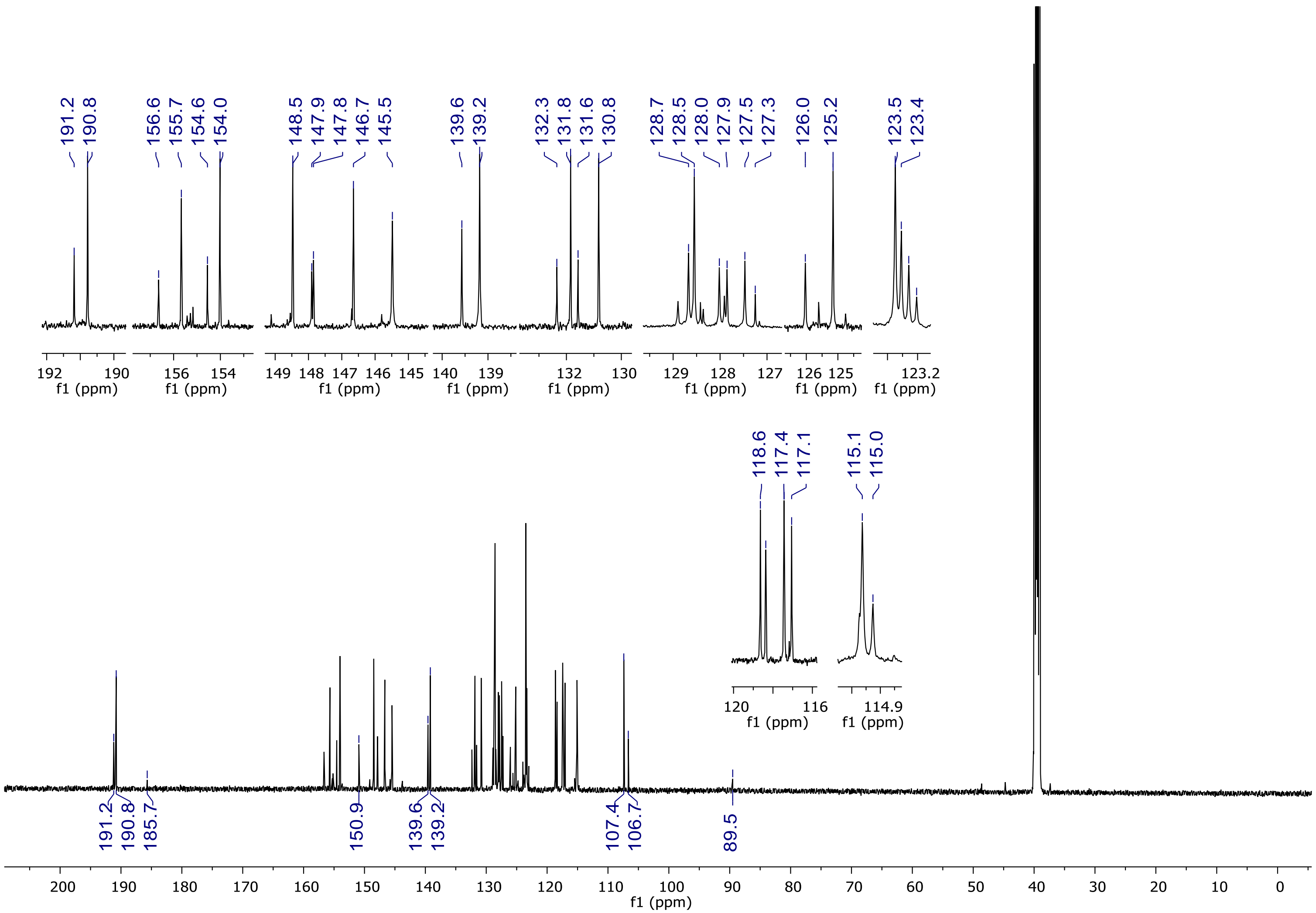

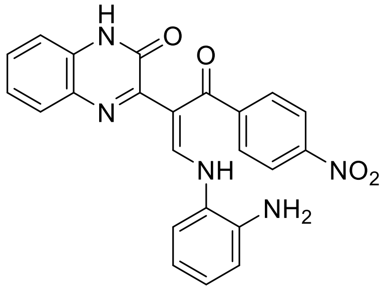

Z

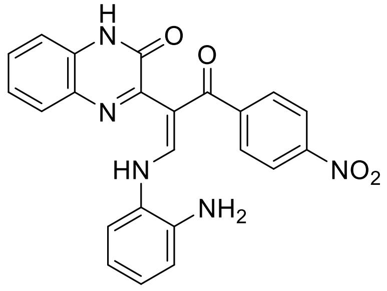

E

2a (***Z*:*E*** ~ 30:70)

Figure S2. $^{13}C\{^{1}H\}$ NMR spectrum of *Z* and *E* isomers mixture of **2a** (DMSO-d_6, 125.77 MHz)

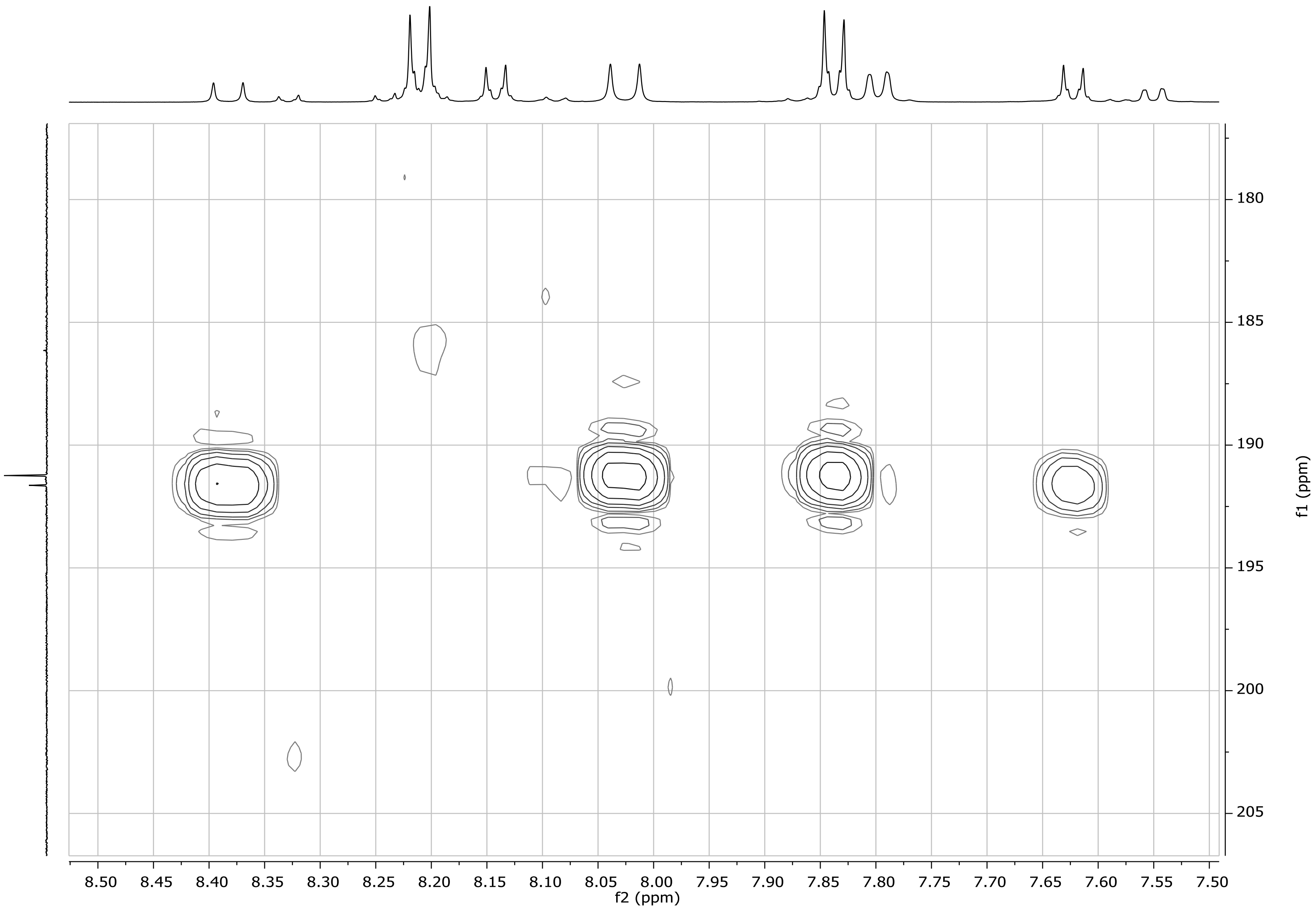

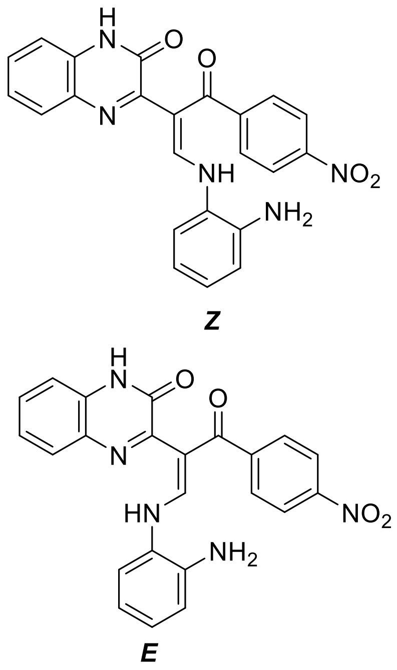

2a (***Z*:*E*** ~ 30:70)

Figure S3. Expansion HMBC spectrum of *Z* and *E* isomers mixture of **2a** (DMSO-d_6, 125.77 MHz)

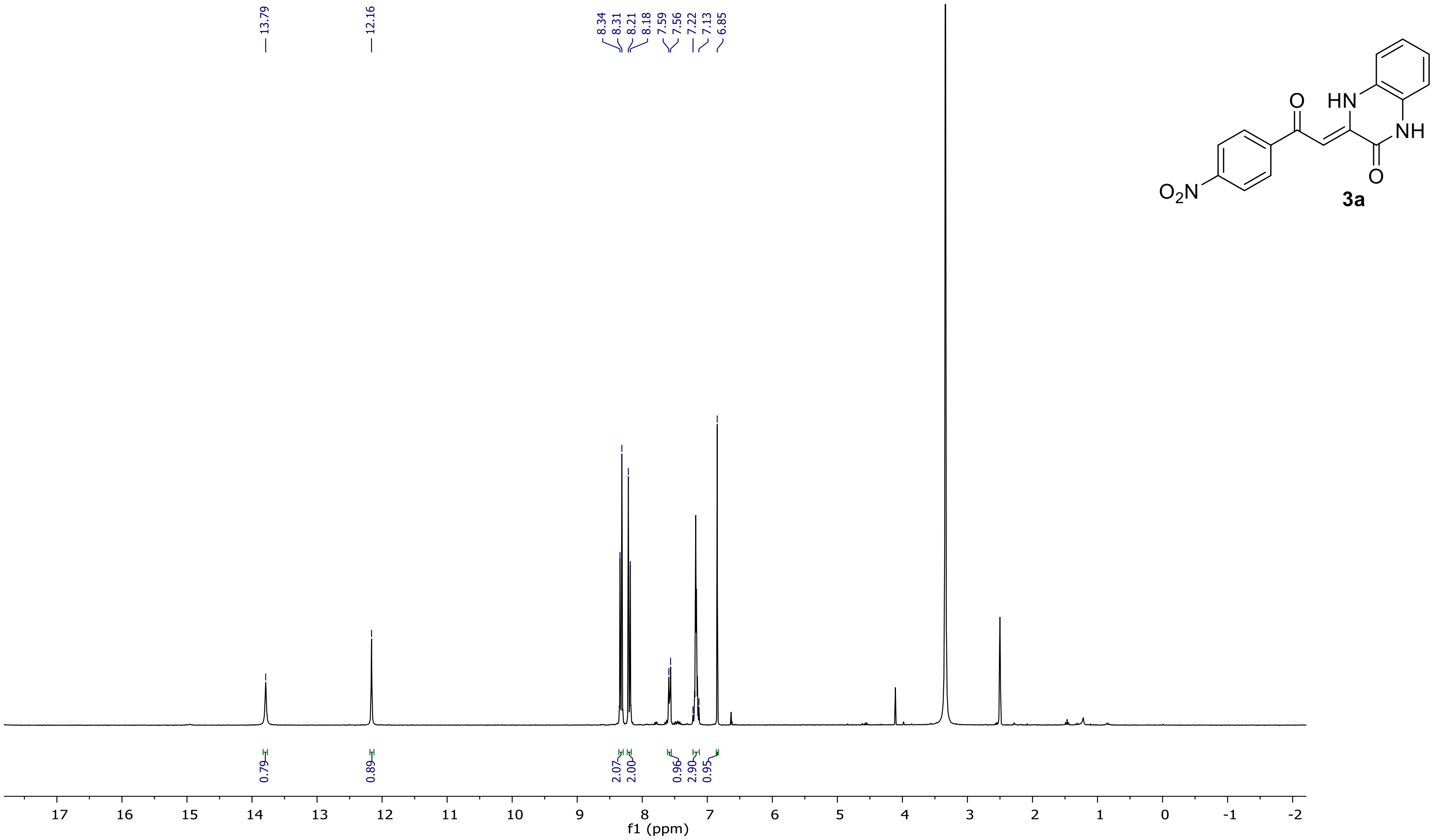

Figure S4. ^{1}H NMR spectrum of **3a** (DMSO-d_6, 300.06 MHz)

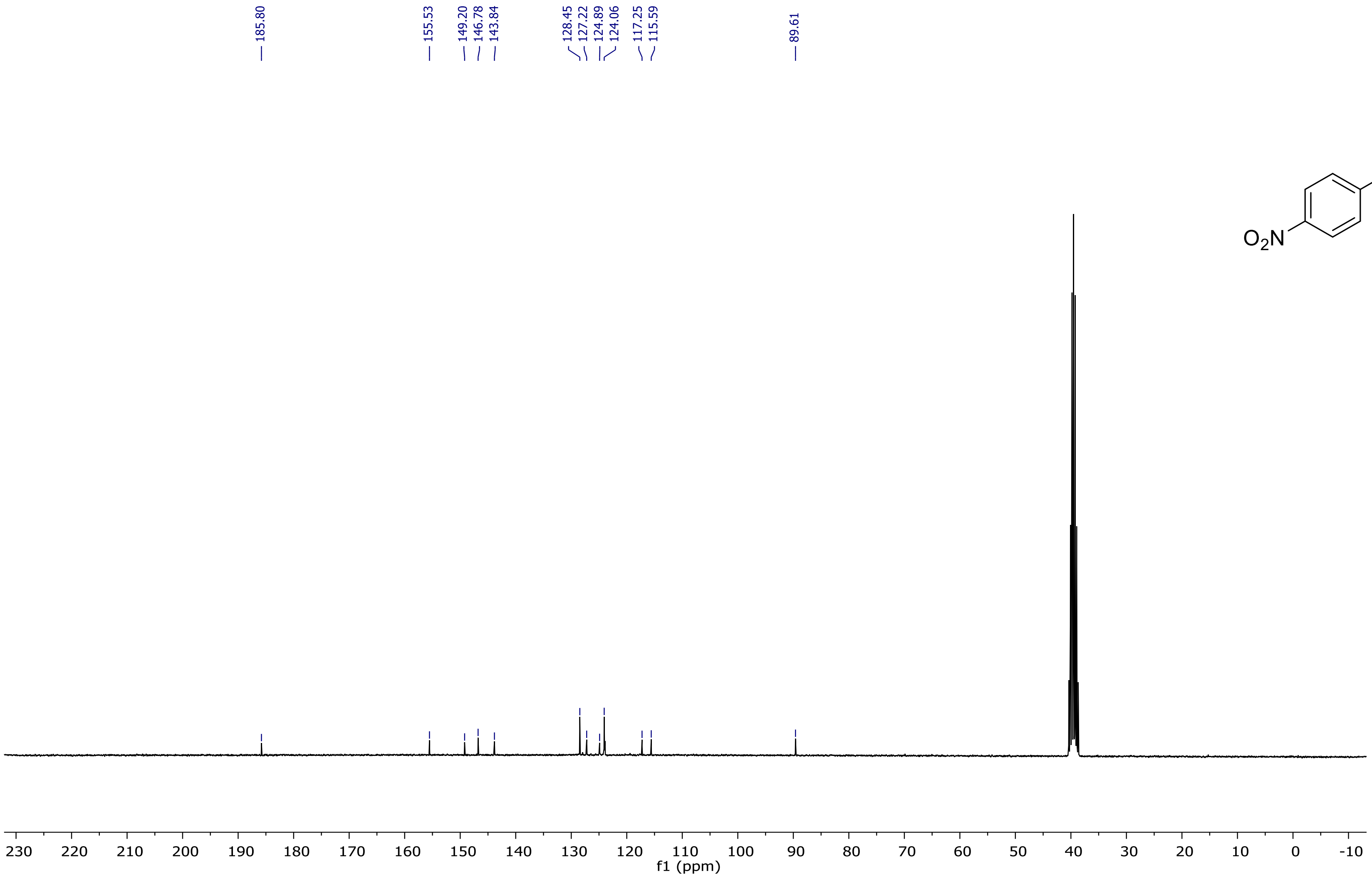

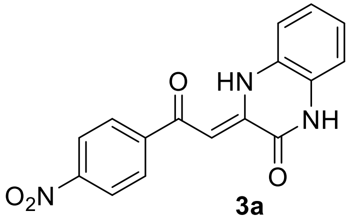

3a

Figure S5. $^{13}C\{^{1}H\}$ NMR spectrum of **3a** (DMSO-d_6, 125.77 MHz)

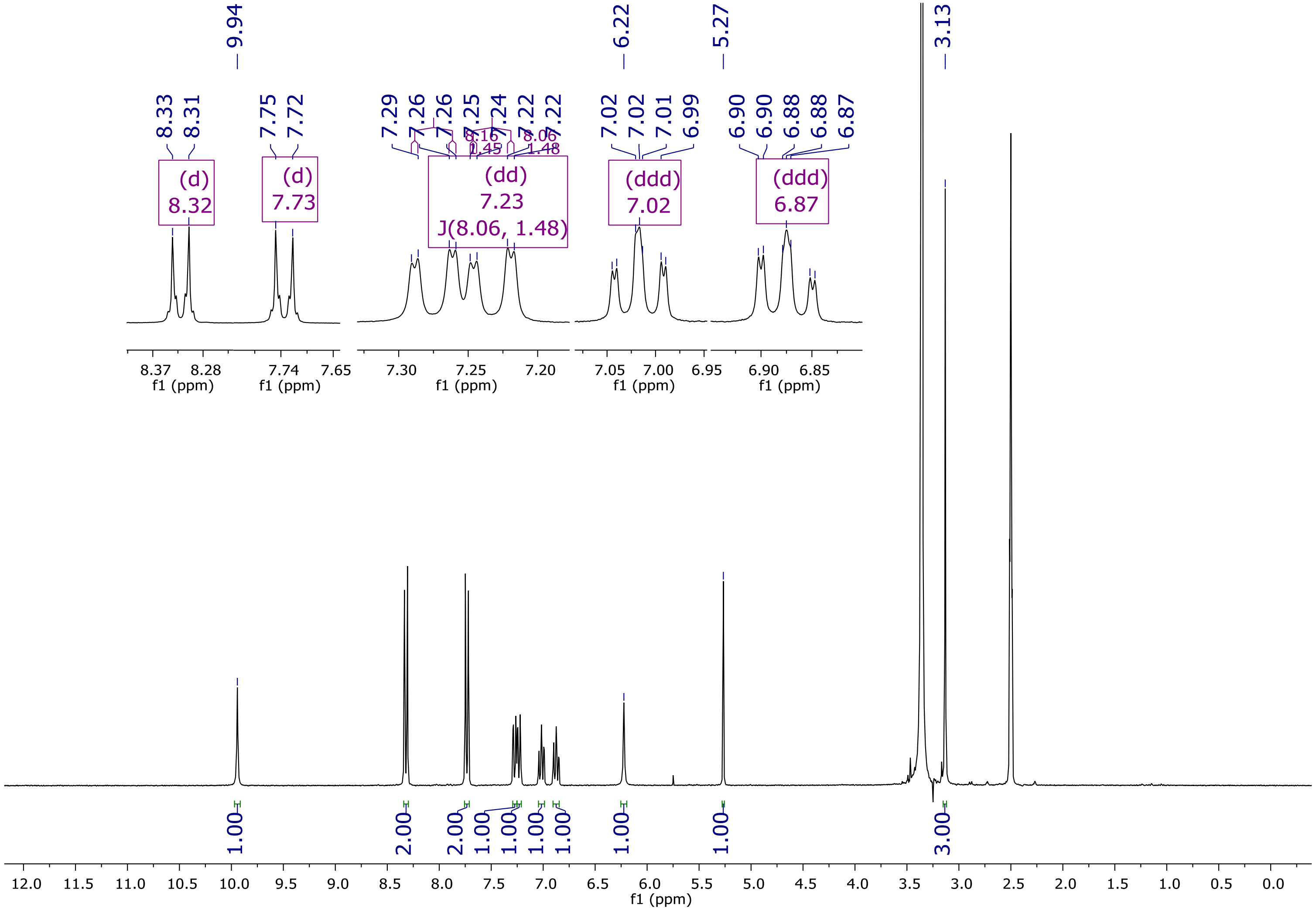

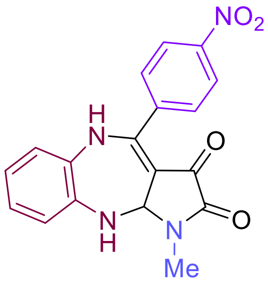

Figure S6. ^{1}H NMR spectrum of **5a** (DMSO-d_6, 300.06 MHz)

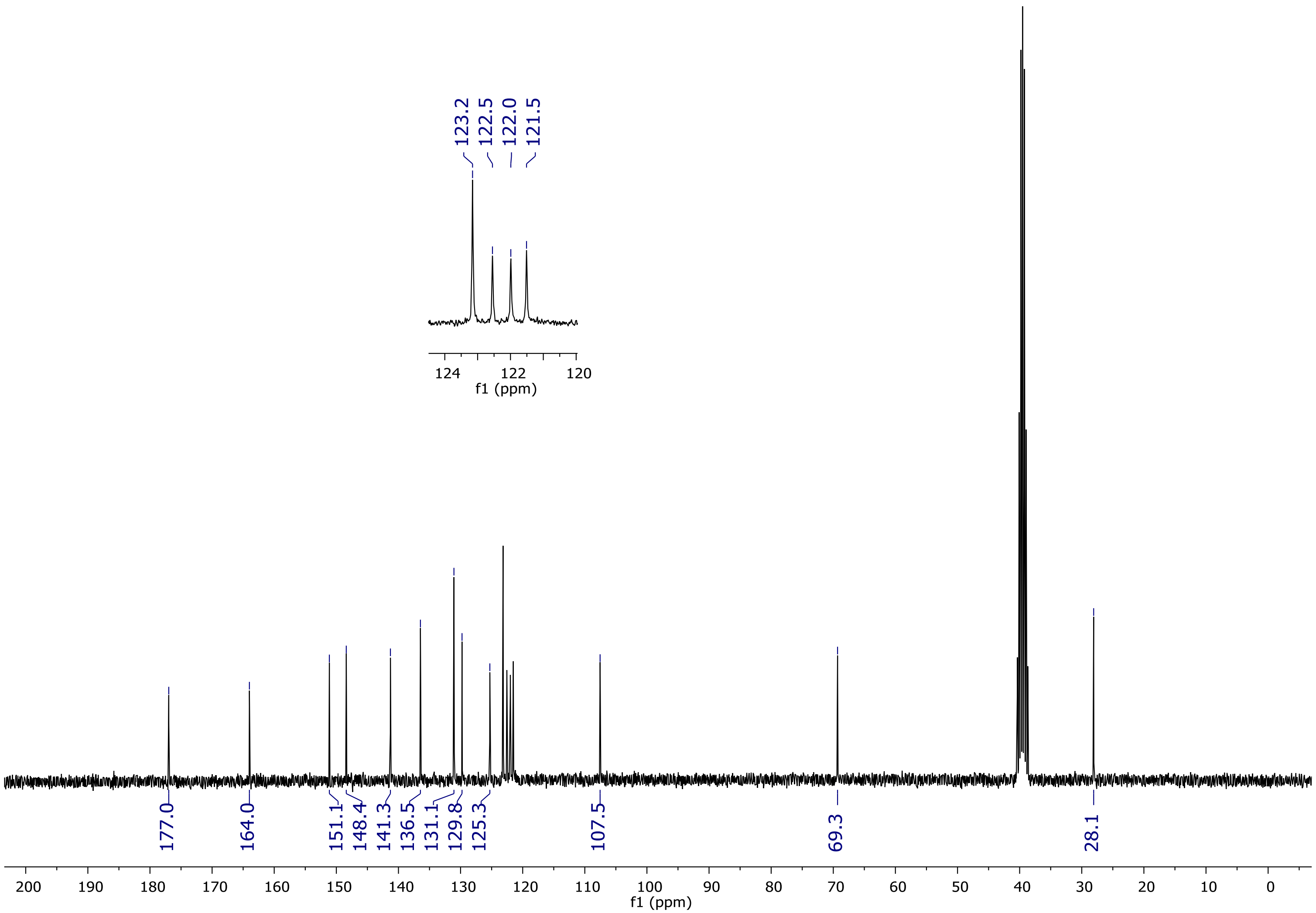

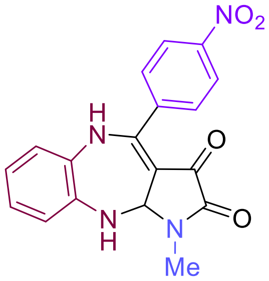

5a

Figure S7. $^{13}C\{^{1}H\}$ NMR spectrum of **5a** (DMSO-d_6, 75.46 MHz)

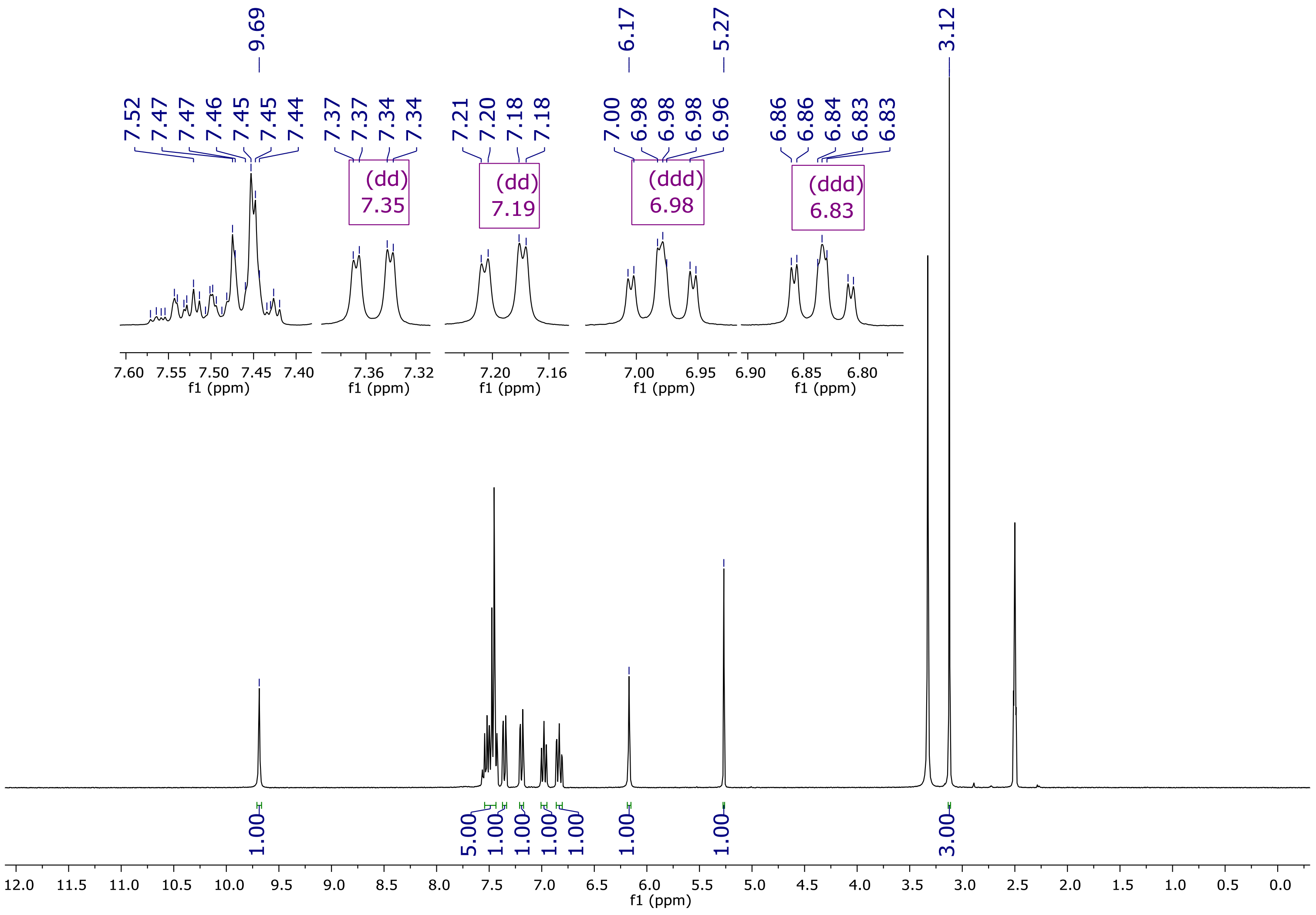

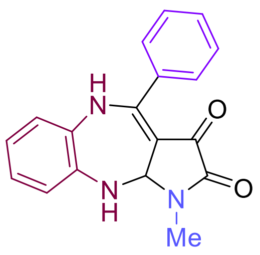
5b

Figure S8. ^{1}H NMR spectrum of **5b** (DMSO-d_6, 300.06 MHz)

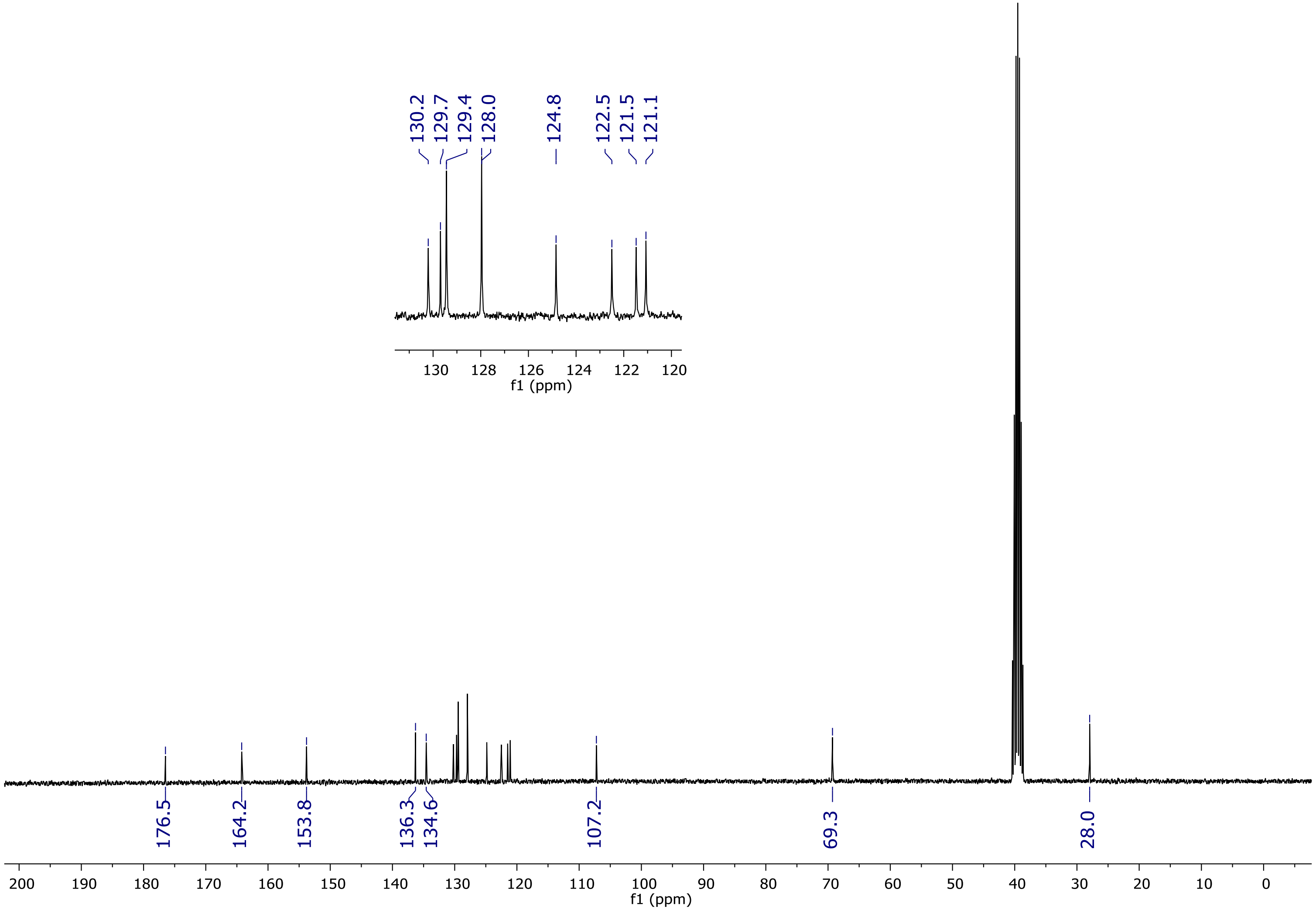

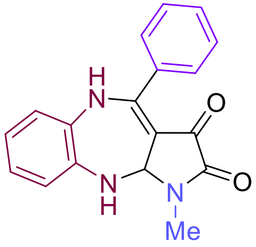

5b

Figure S9. $^{13}C\{^{1}H\}$ NMR spectrum of **5b** (DMSO-d_6, 75.46 MHz)

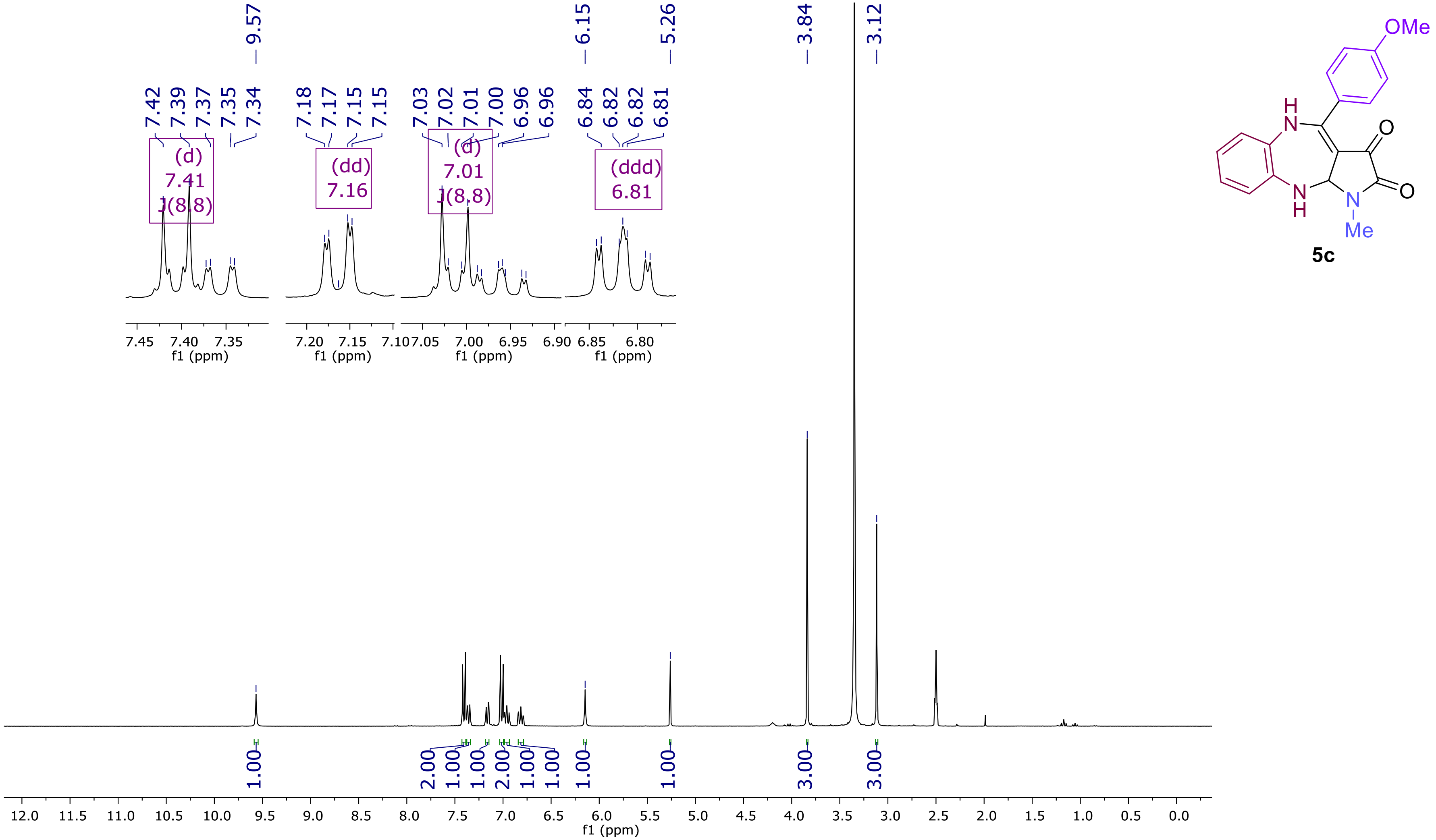

Figure S10. ^{1}H NMR spectrum of **5c** (DMSO-d_6, 300.06 MHz)

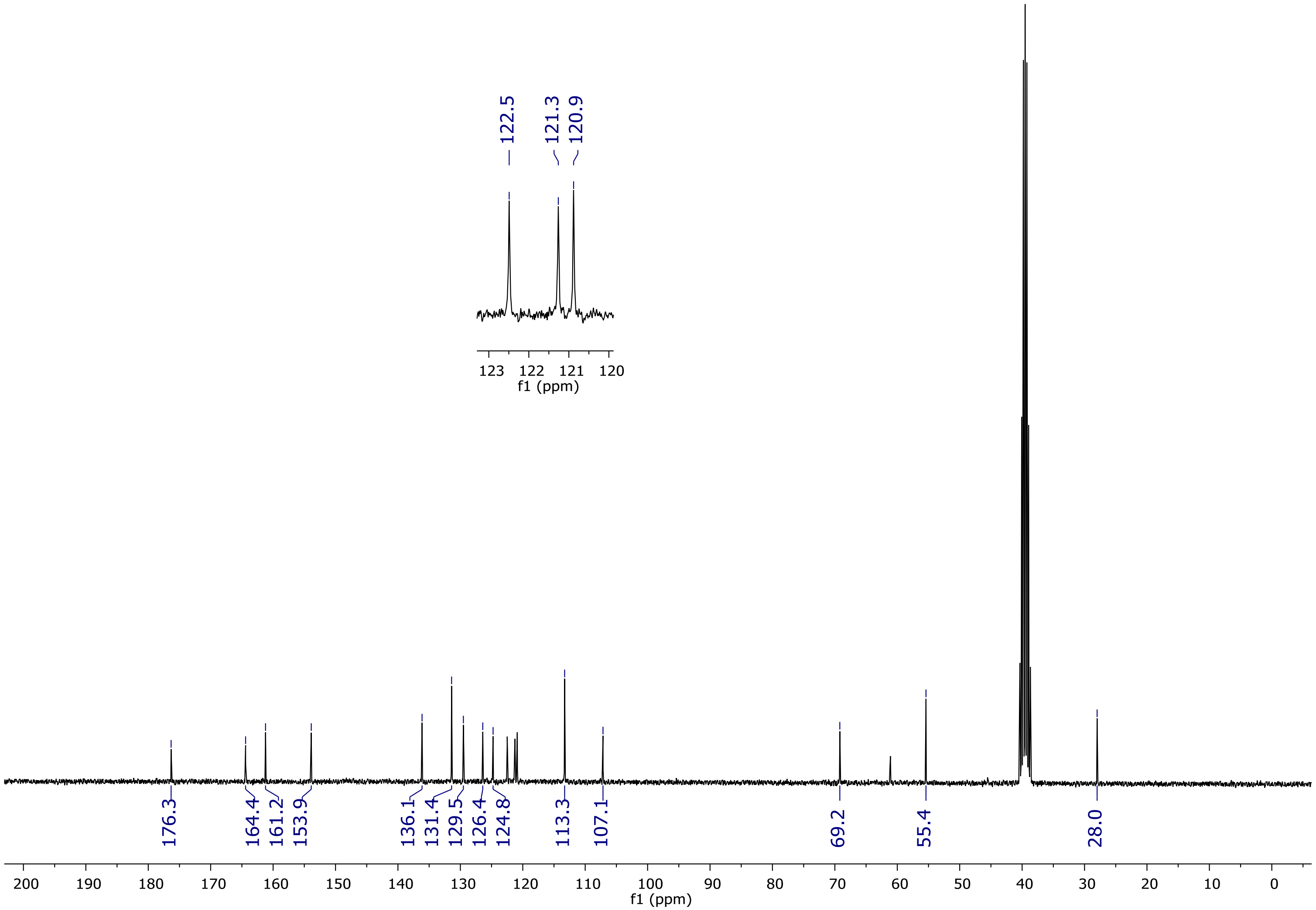

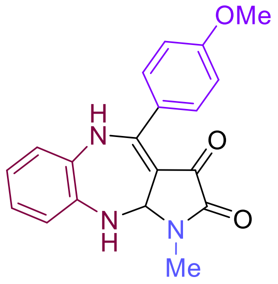

5c

Figure S11. $^{13}C\{^1H\}$ NMR spectrum of **5c** (DMSO-d_6, 75.46 MHz)

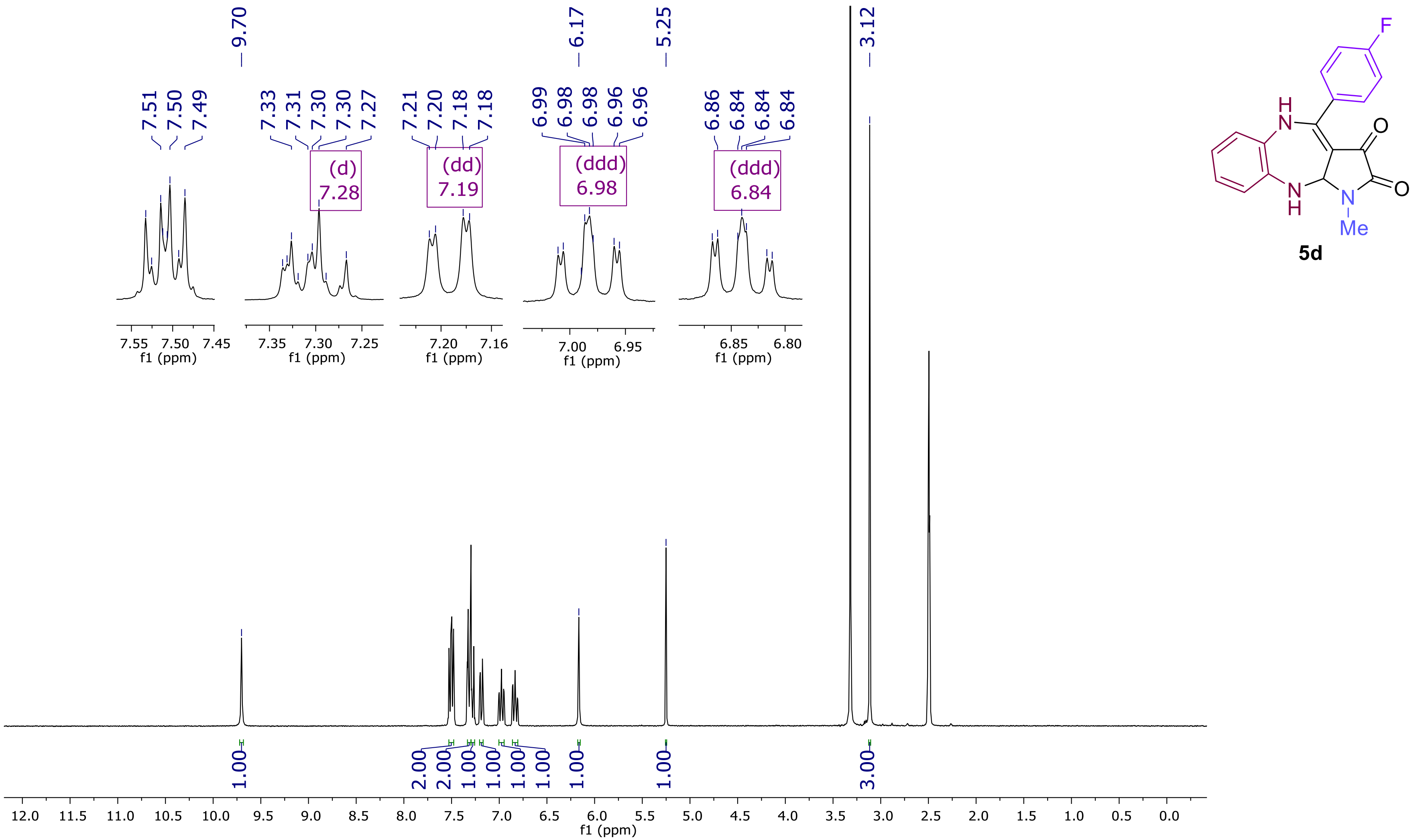

Figure S12. ^{1}H NMR spectrum of **5d** (DMSO-d_6, 300.06 MHz)

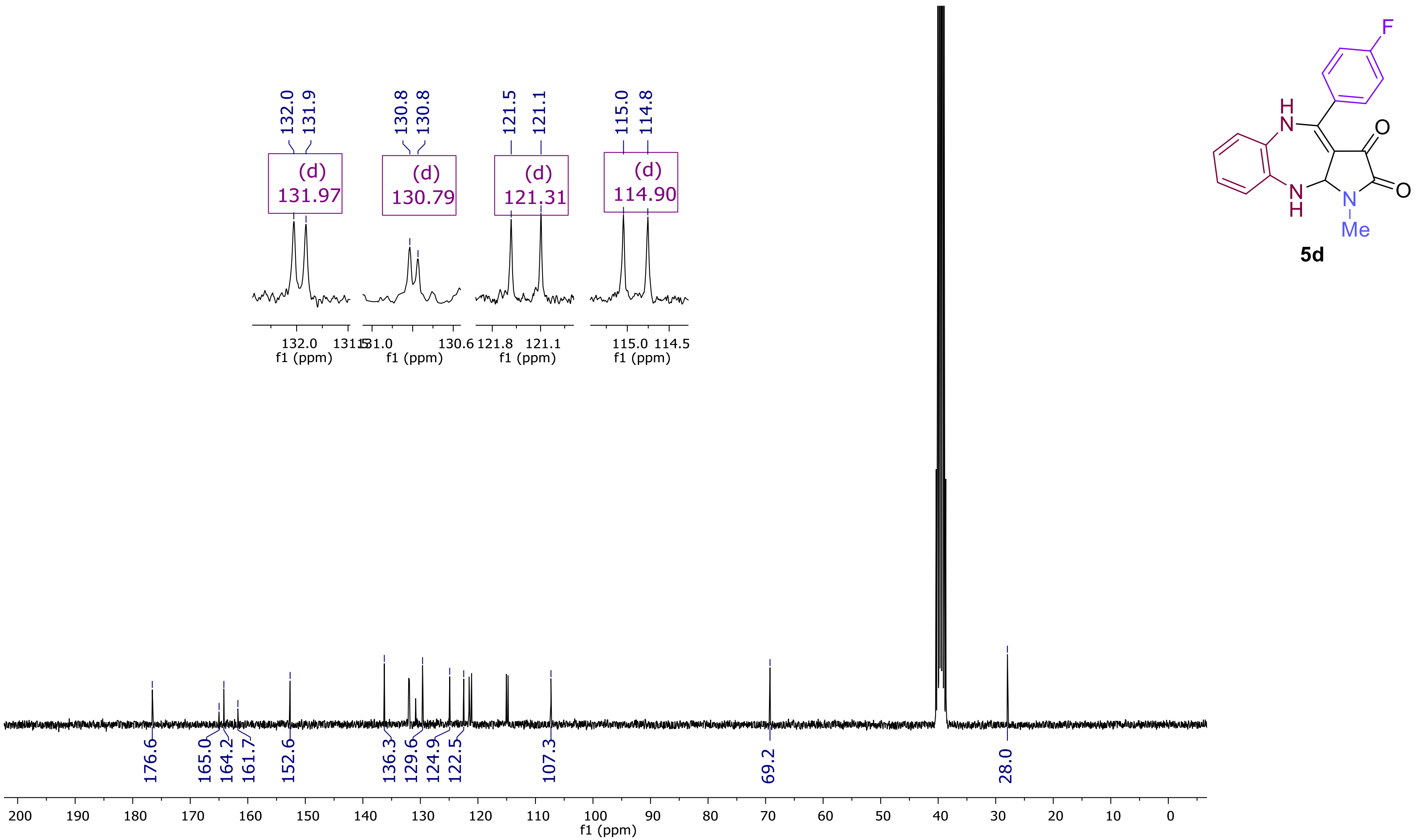

Figure S13. $^{13}C\{^{1}H\}$ NMR spectrum of **5d** (DMSO-d_6, 75.46 MHz)

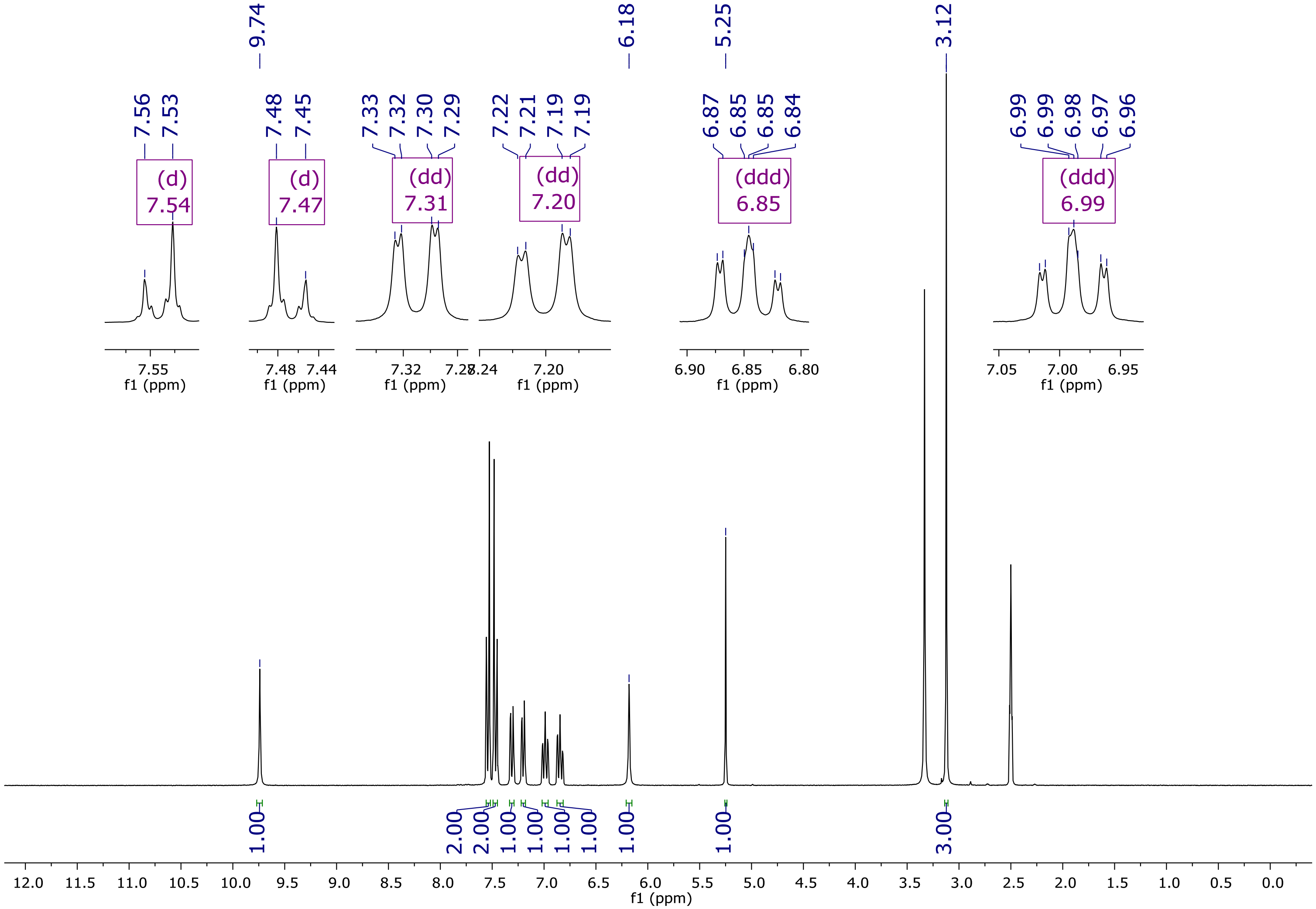

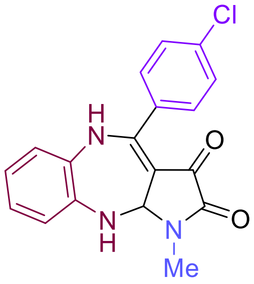

5e

Figure S14. ^{1}H NMR spectrum of **5e** (DMSO-d_6, 300.06 MHz)

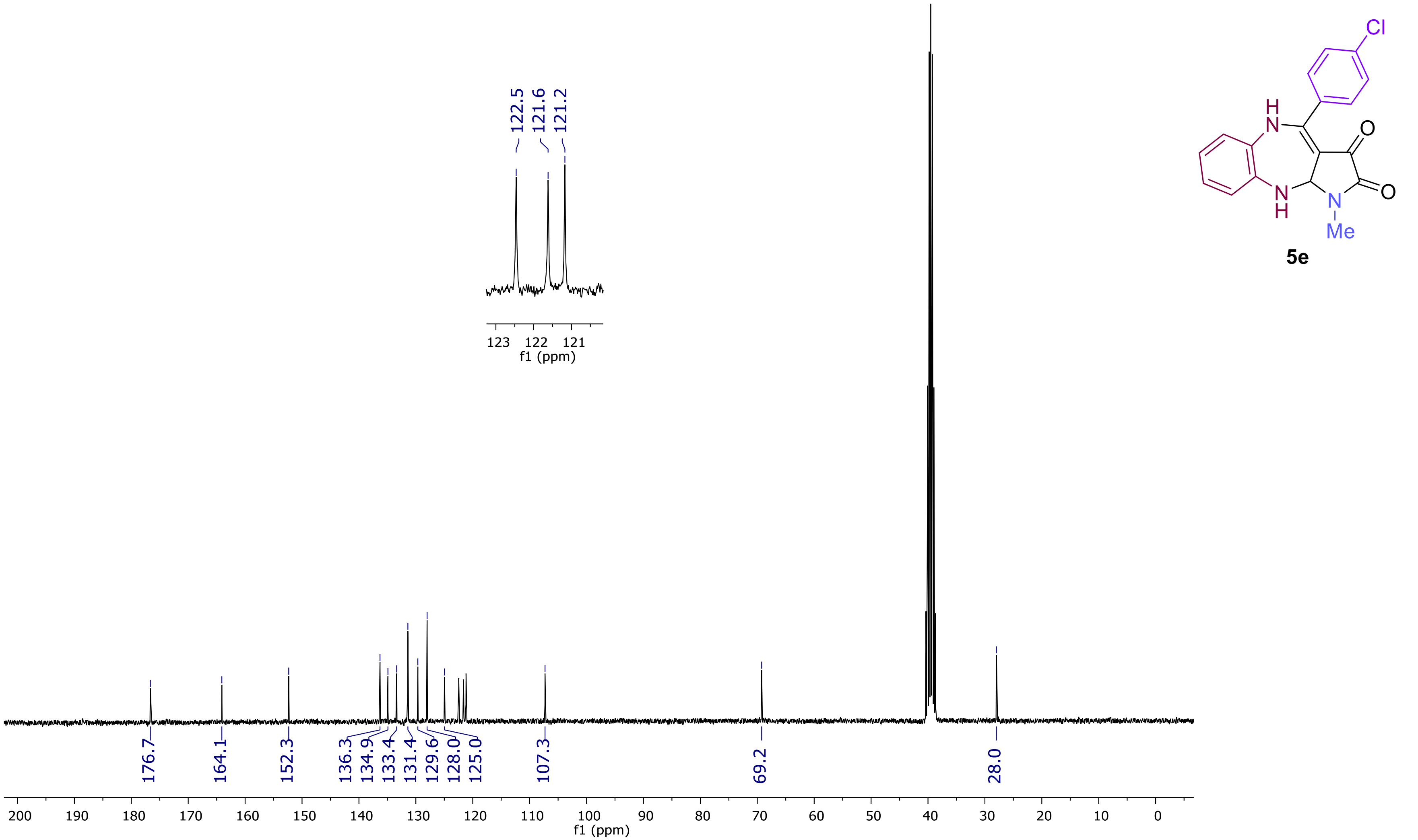

Figure S15. $^{13}C\{^{1}H\}$ NMR spectrum of **5e** (DMSO-d_6, 75.46 MHz)

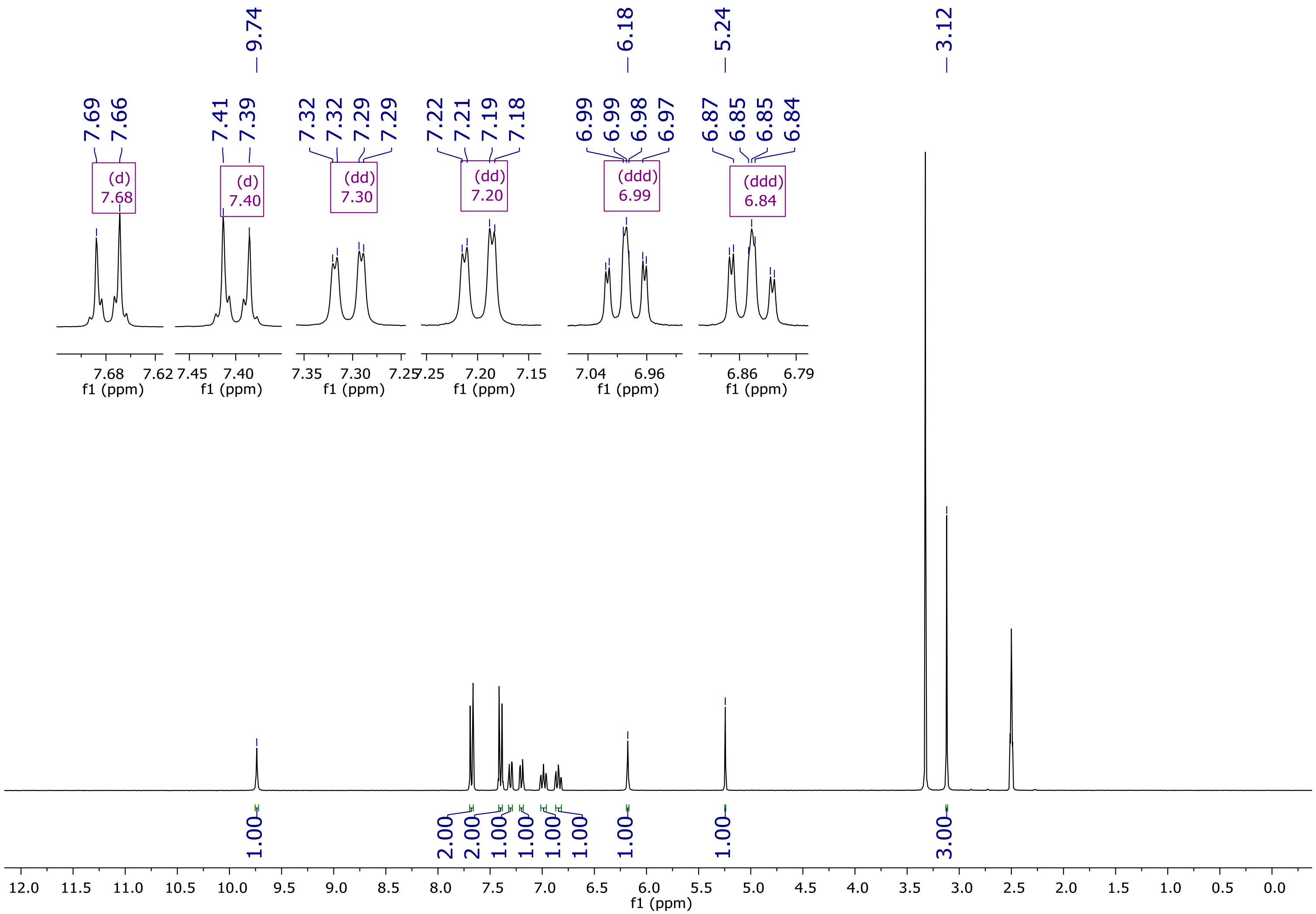

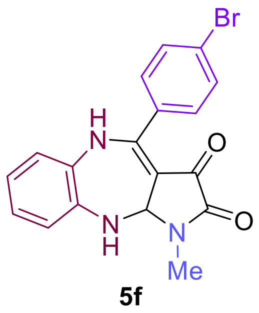

Figure S16. ^{1}H NMR spectrum of **5f** (DMSO-d_6, 300.06 MHz)

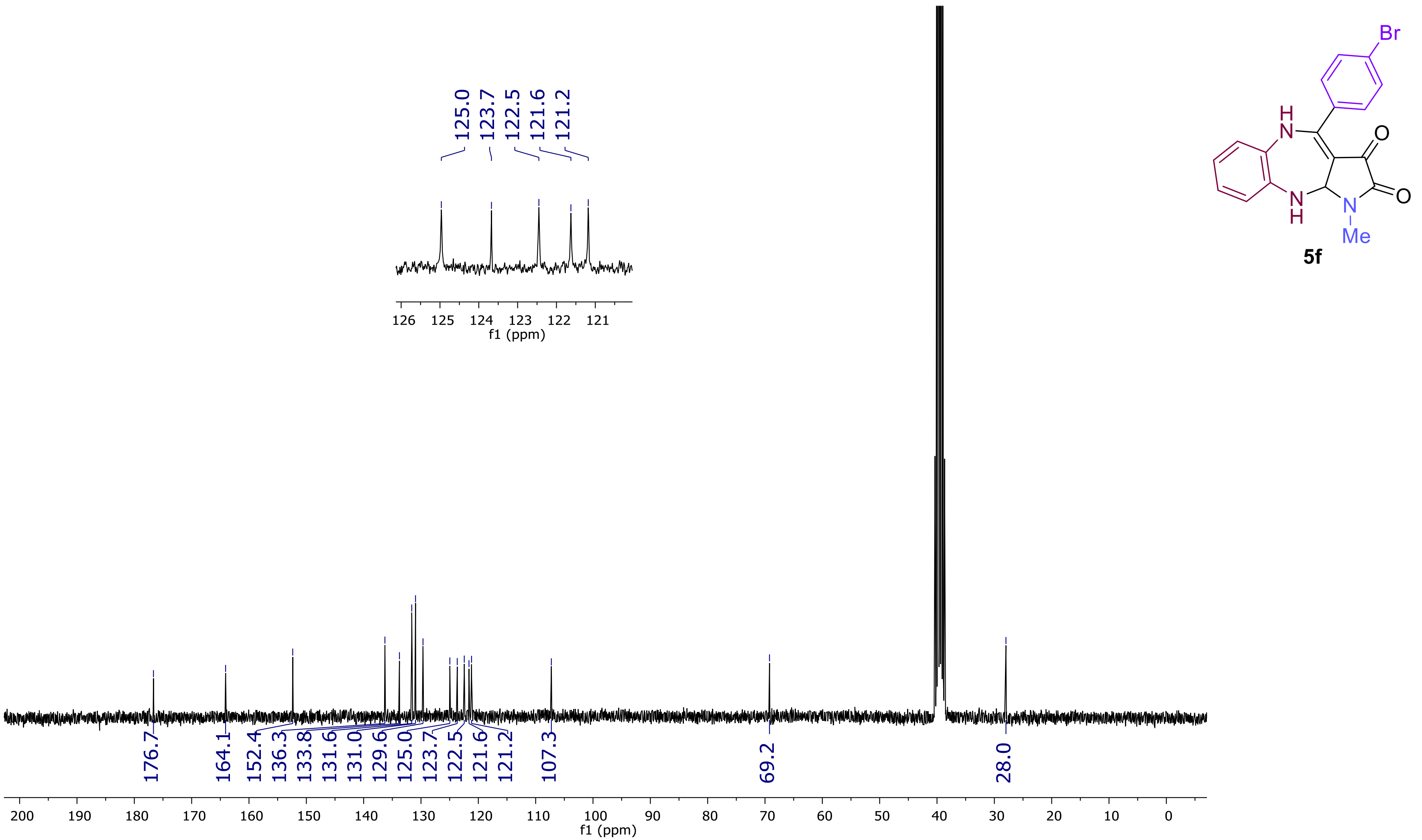

Figure S17. $^{13}C\{^{1}H\}$ NMR spectrum of **5f** (DMSO-d_6, 75.46 MHz)

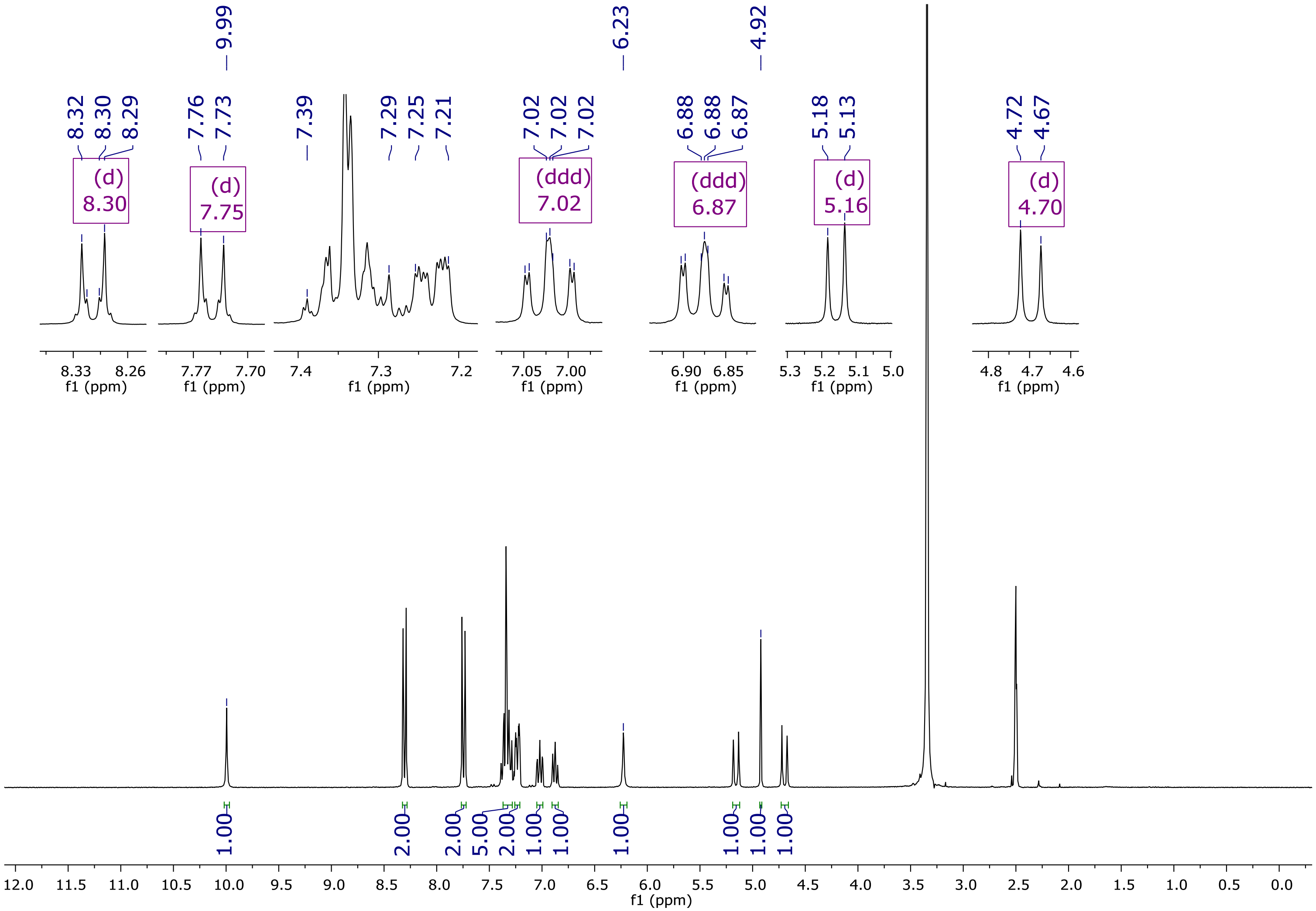

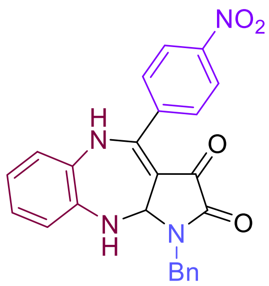

5g

Figure S18. ^{1}H NMR spectrum of **5g** (DMSO-d_6, 300.06 MHz)

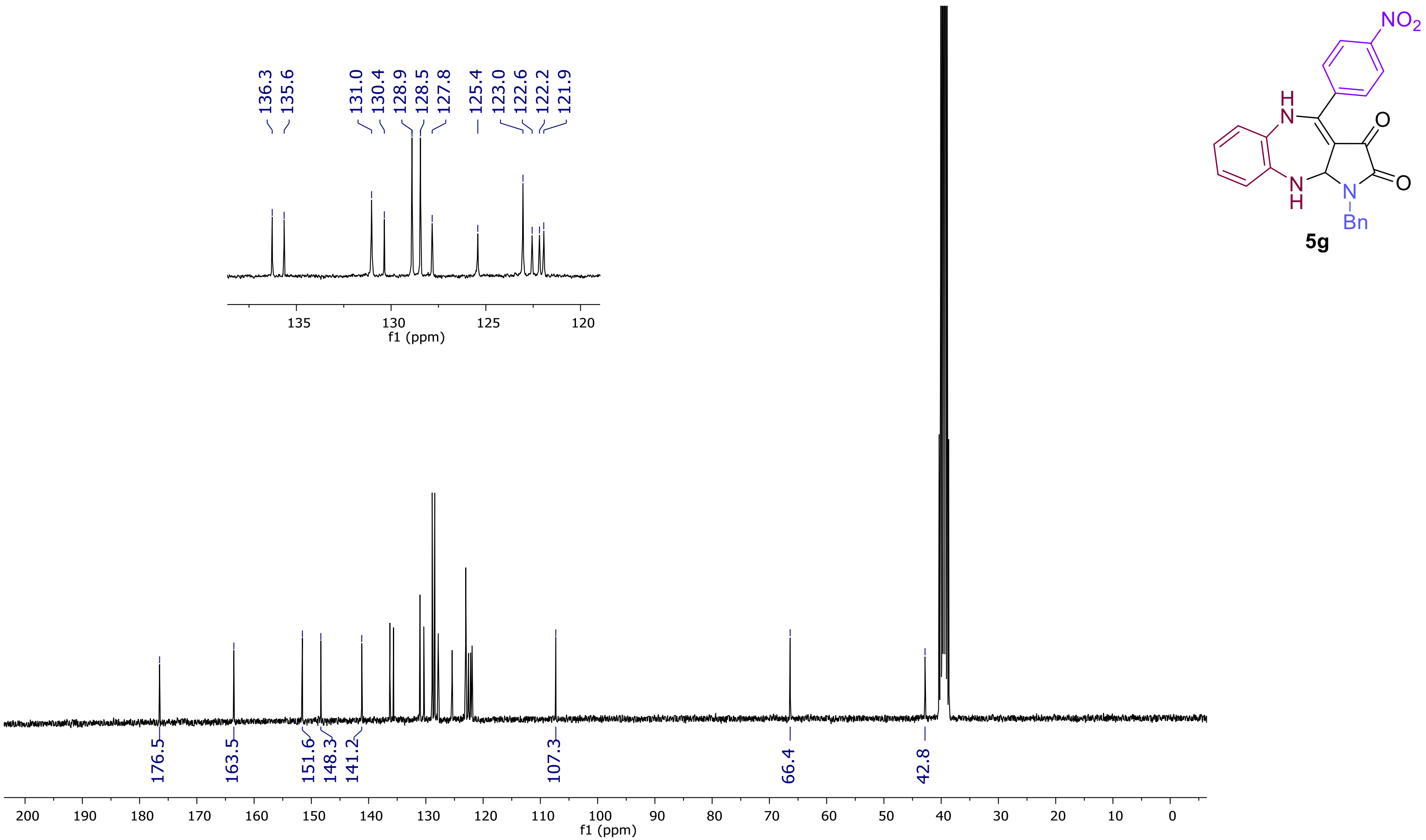

Figure S19. $^{13}C\{^{1}H\}$ NMR spectrum of **5g** (DMSO-d_6, 75.46 MHz)

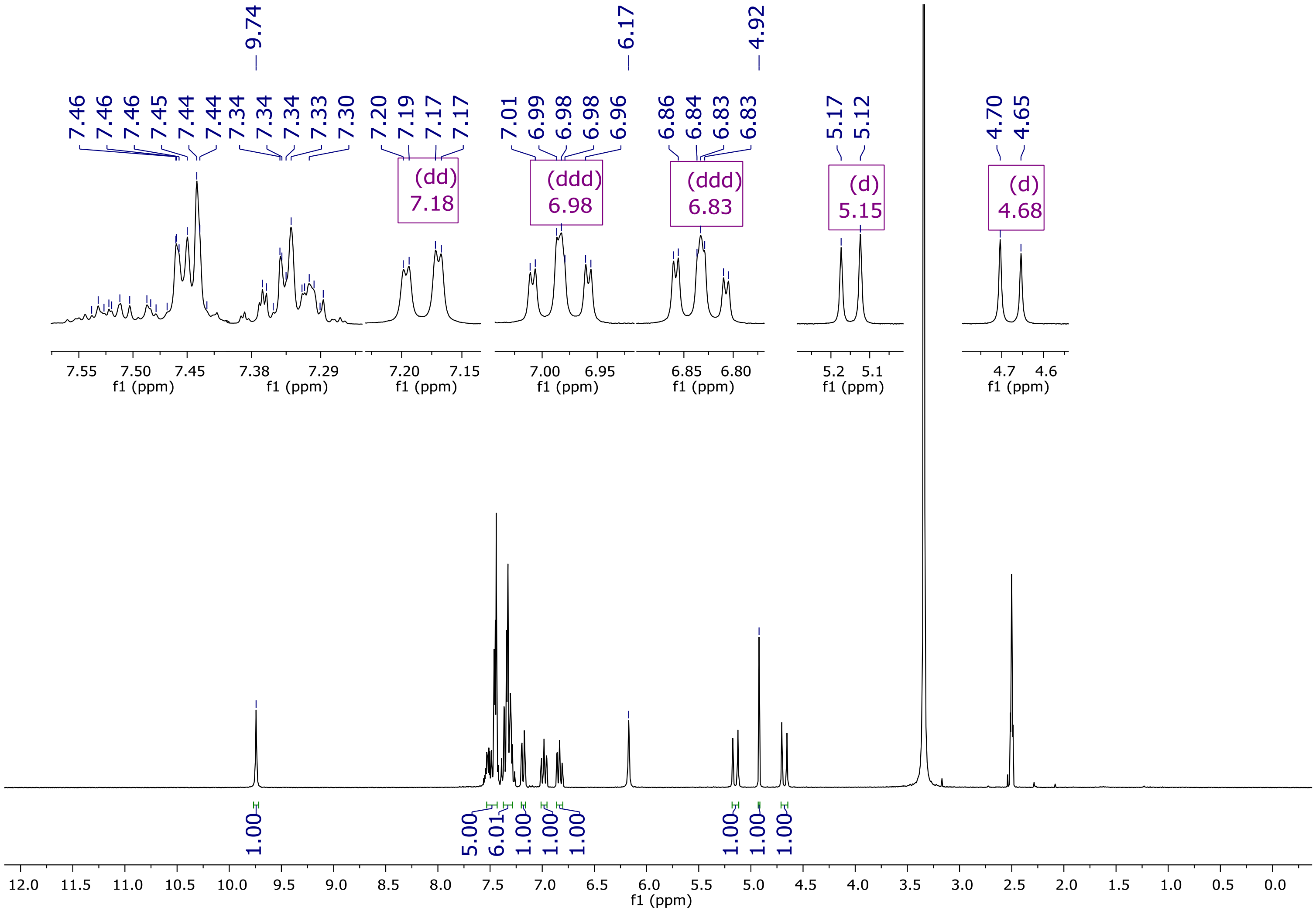

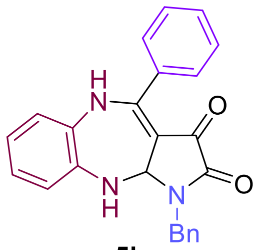

5h

Figure S20. ^{1}H NMR spectrum of **5h** (DMSO-d_6, 300.06 MHz)

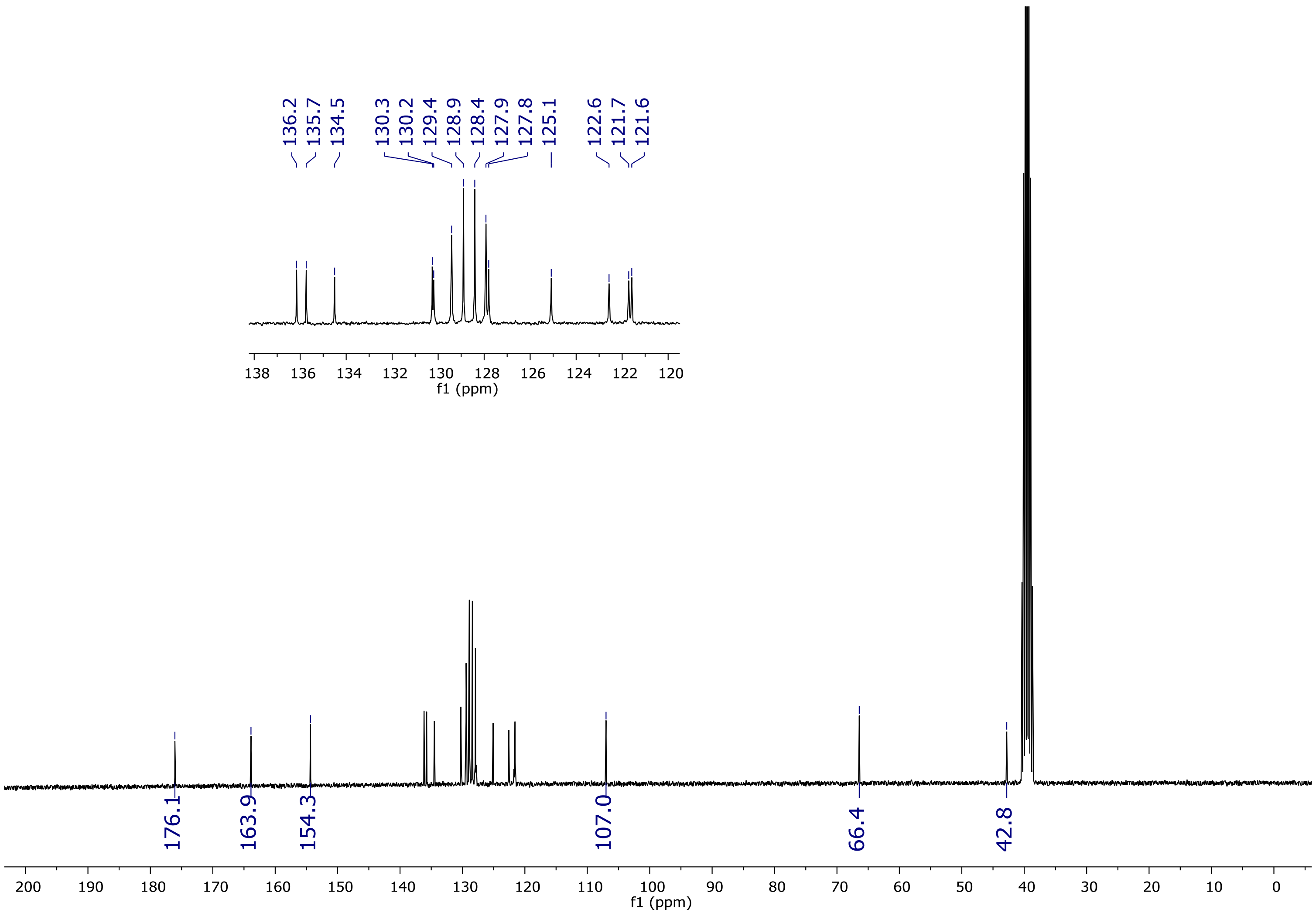

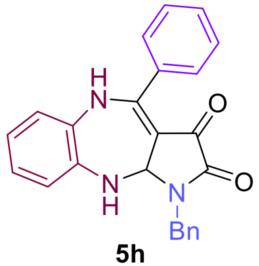

Figure S21. $^{13}C\{^{1}H\}$ NMR spectrum of **5h** (DMSO-d_6, 75.46 MHz)

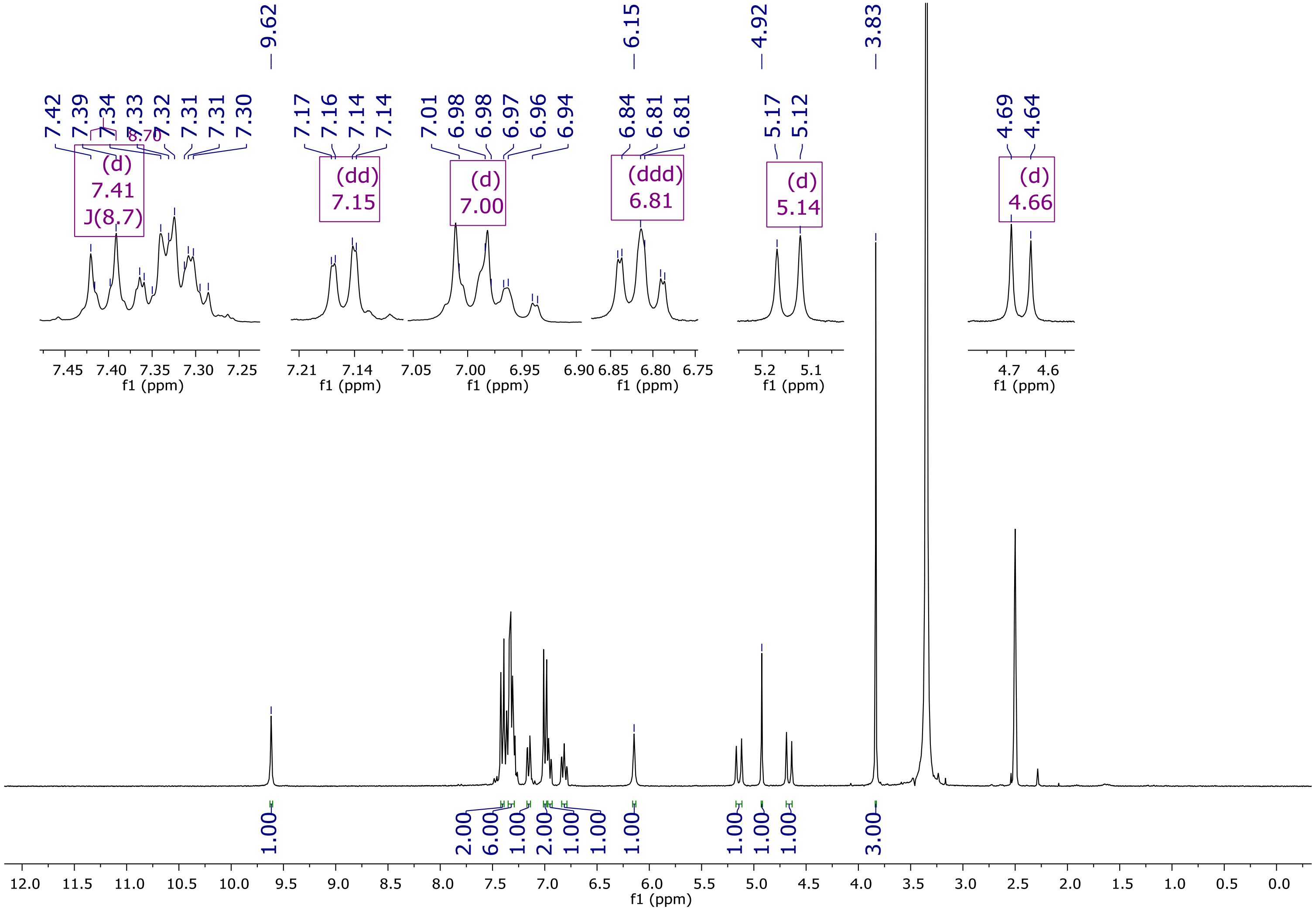

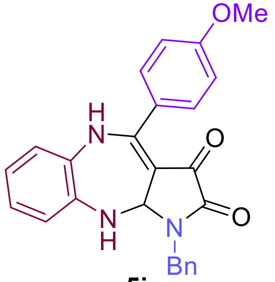

5i

Figure S22. ^{1}H NMR spectrum of **5i** (DMSO-d_6, 300.06 MHz)

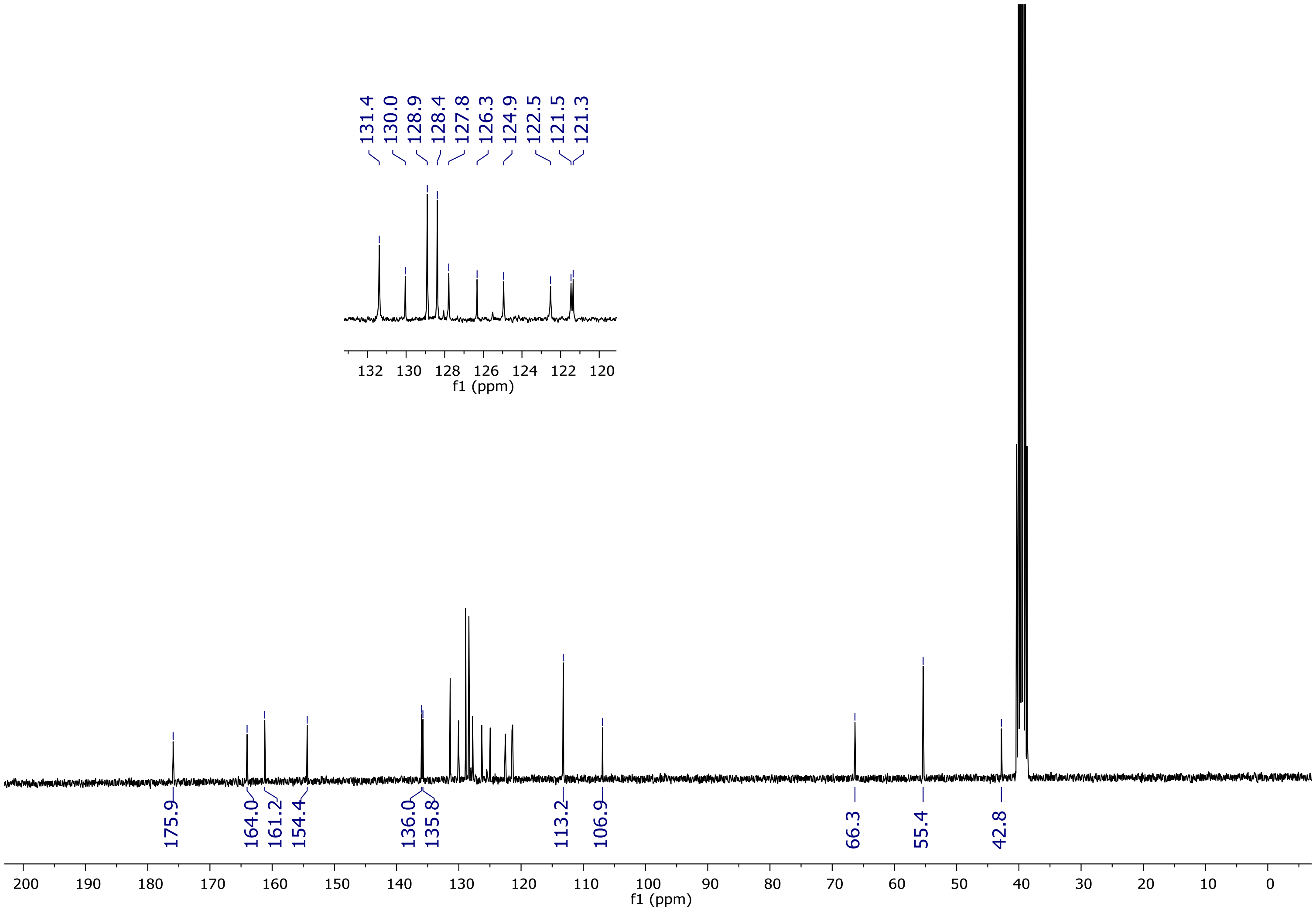

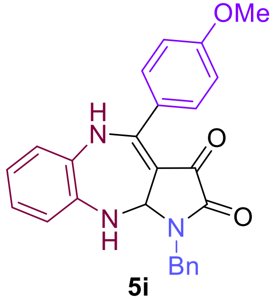

5i

Figure S23. $^{13}C\{^{1}H\}$ NMR spectrum of **5i** (DMSO-d_6, 75.46 MHz)

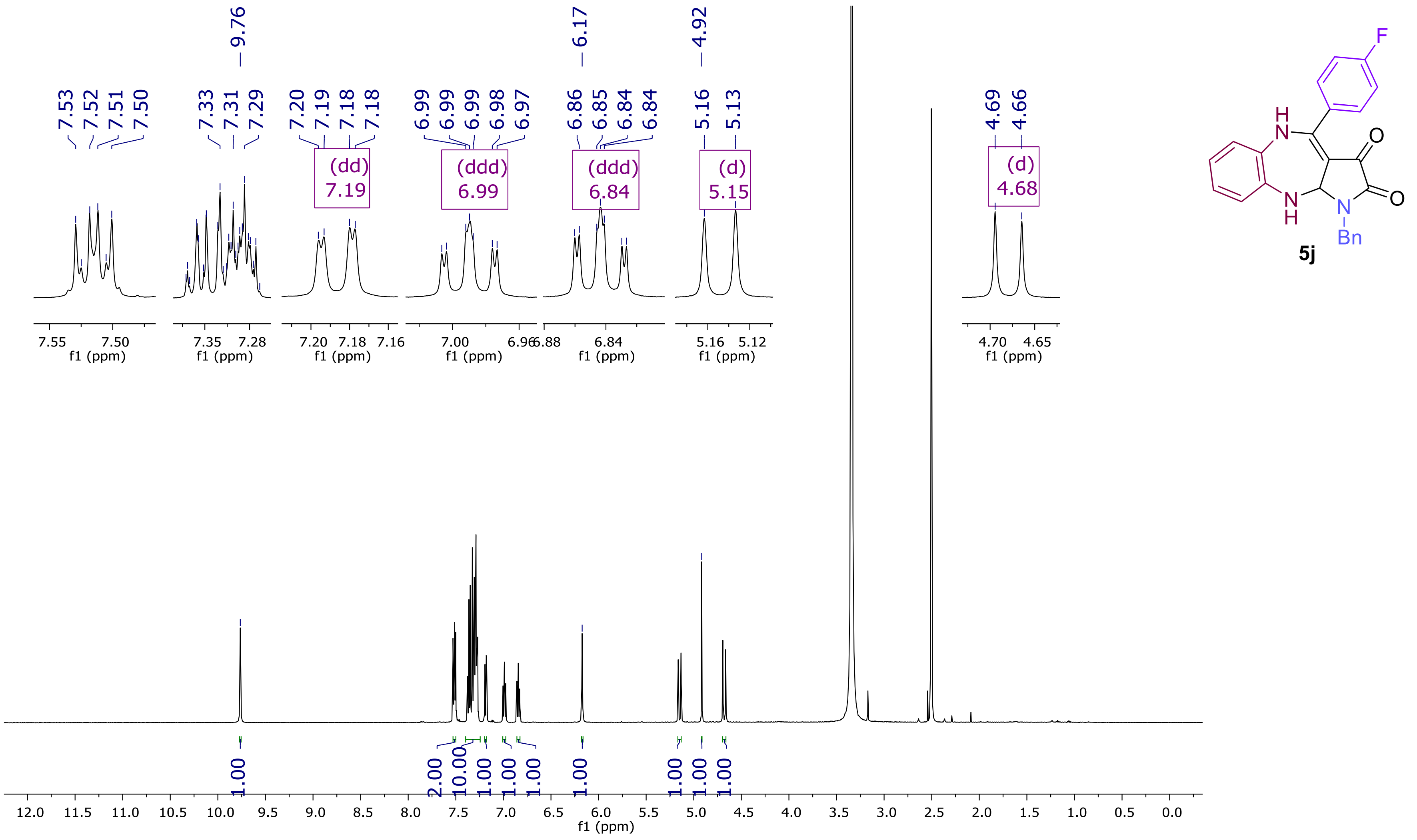

Figure S24. ^{1}H NMR spectrum of **5j** (DMSO-d_6, 500.13 MHz)

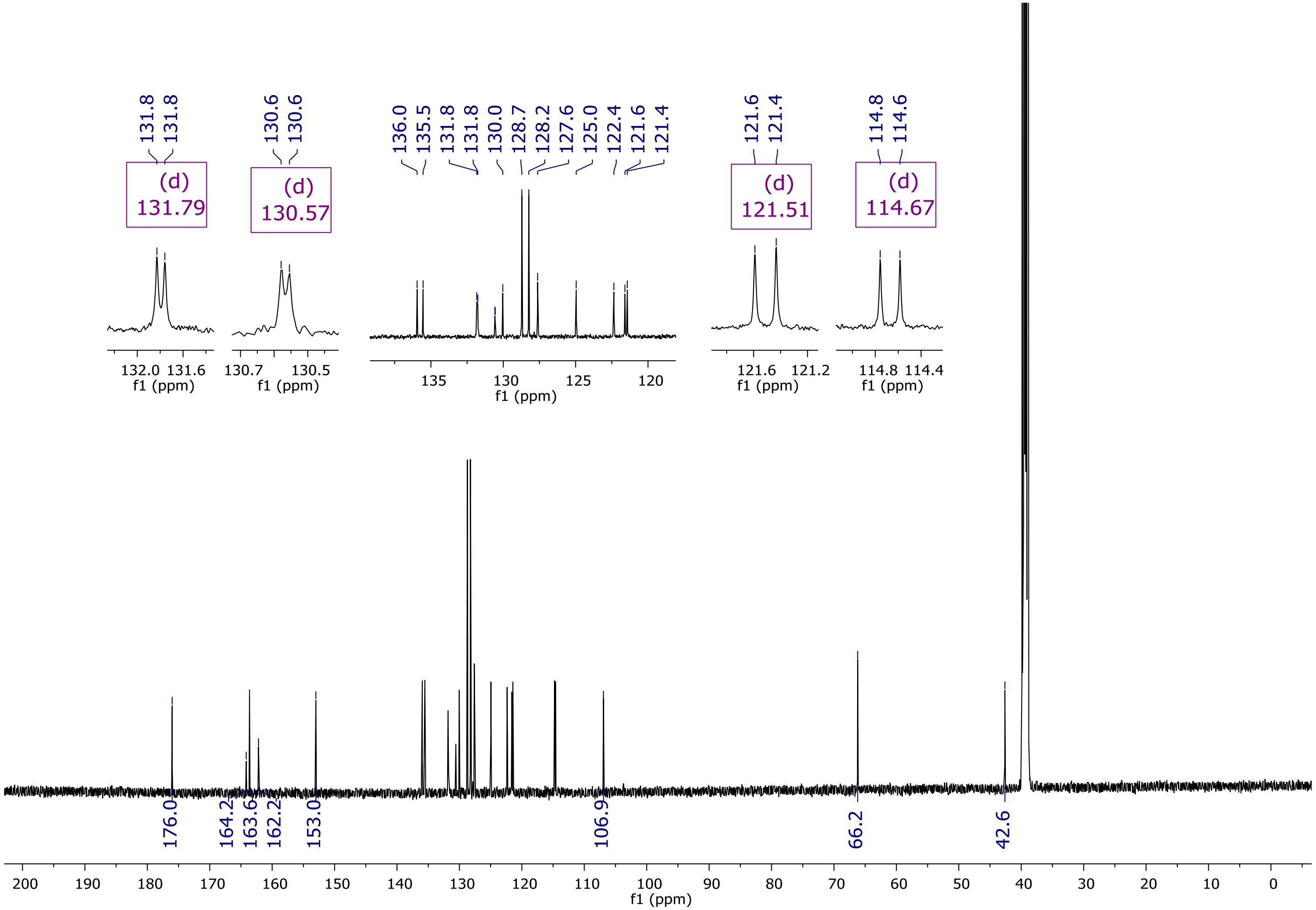

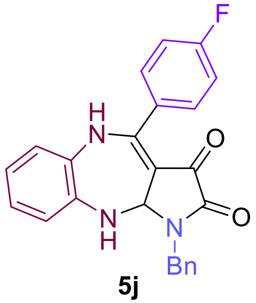

5j

Figure S25. $^{13}C\{^{1}H\}$ NMR spectrum of **5j** (DMSO-d_6, 125.77 MHz)

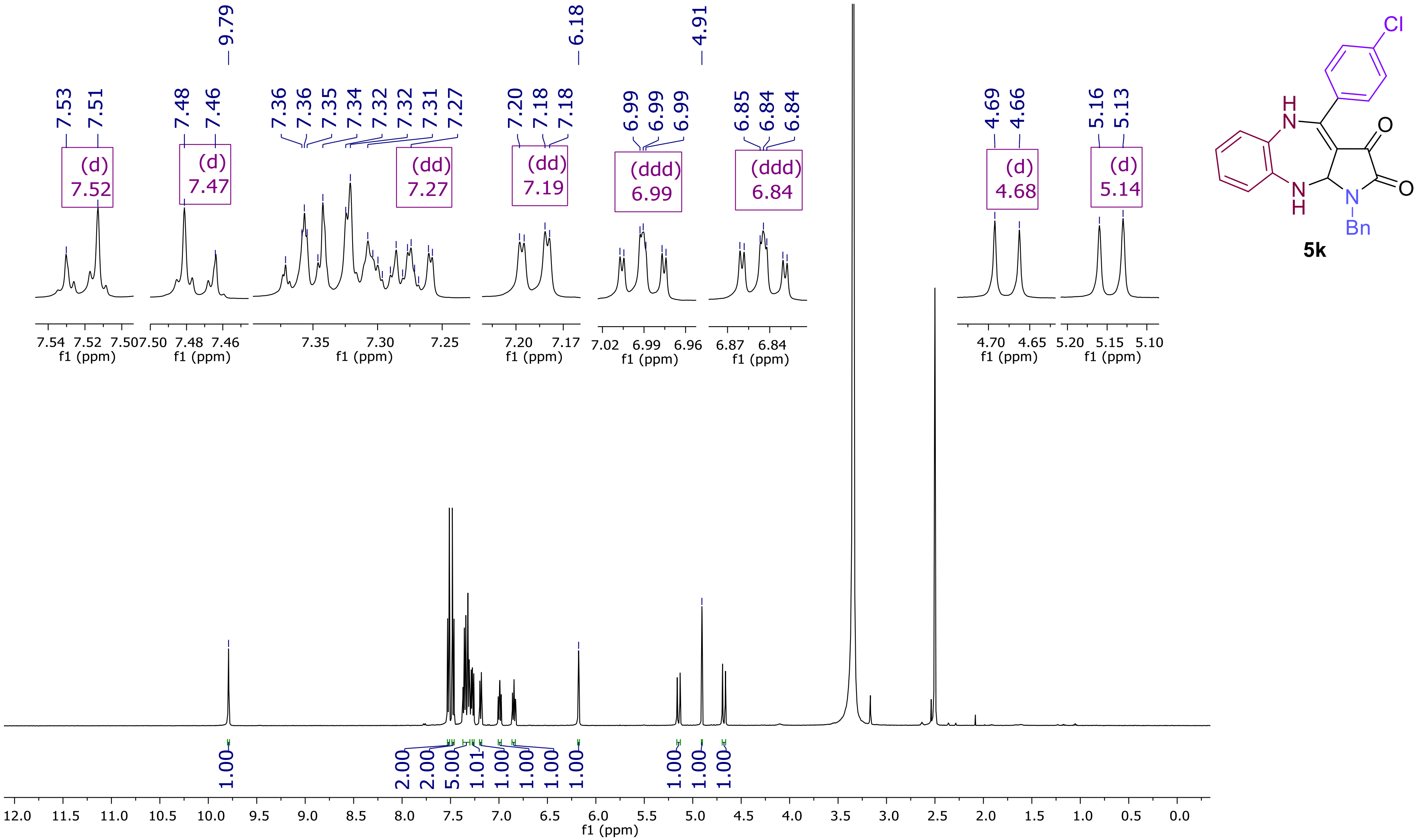

Figure S26. ^{1}H NMR spectrum of **5k** (DMSO-d_6, 500.13 MHz)

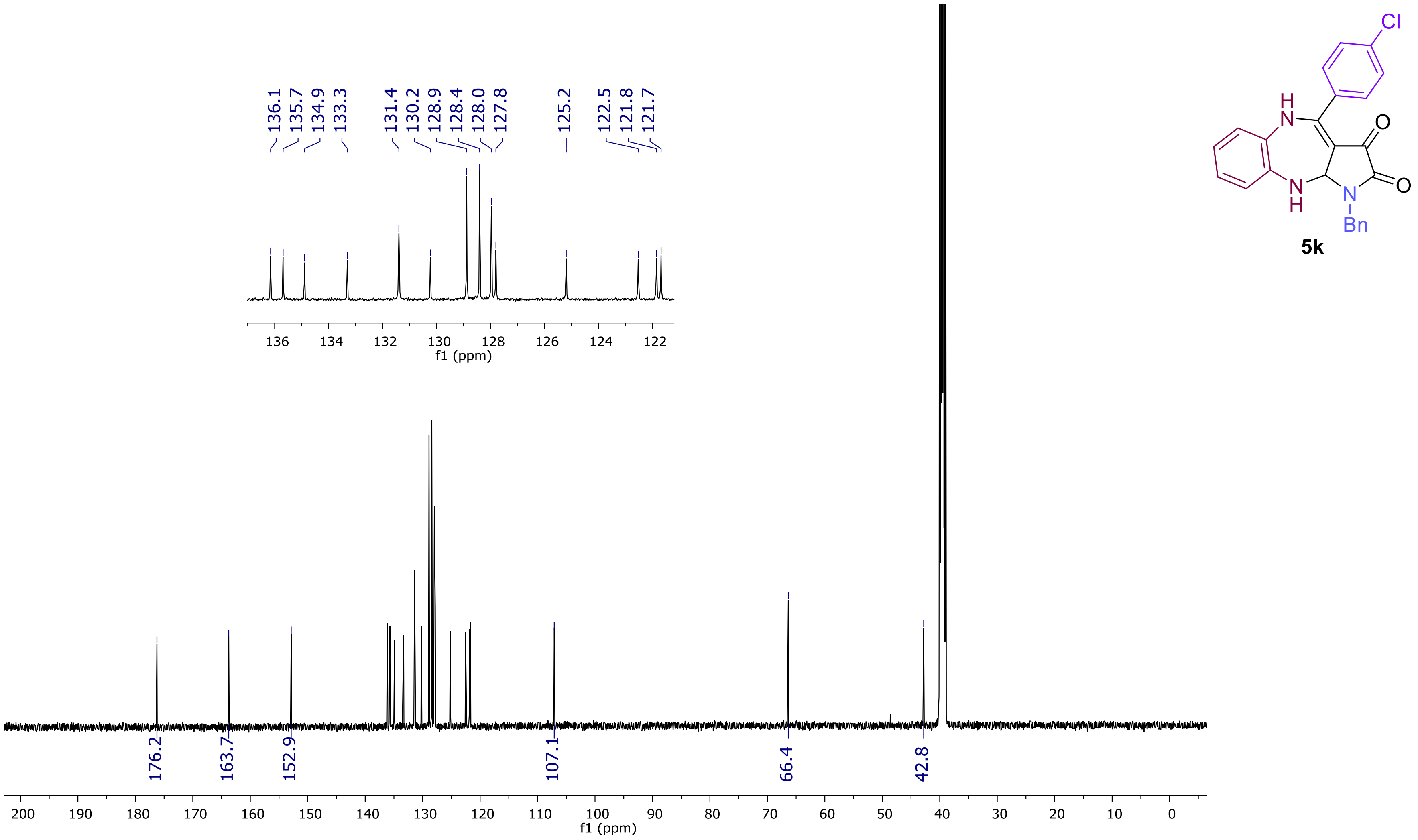

Figure S27. $^{13}C\{^{1}H\}$ NMR spectrum of **5k** (DMSO-d_6, 125.77 MHz)

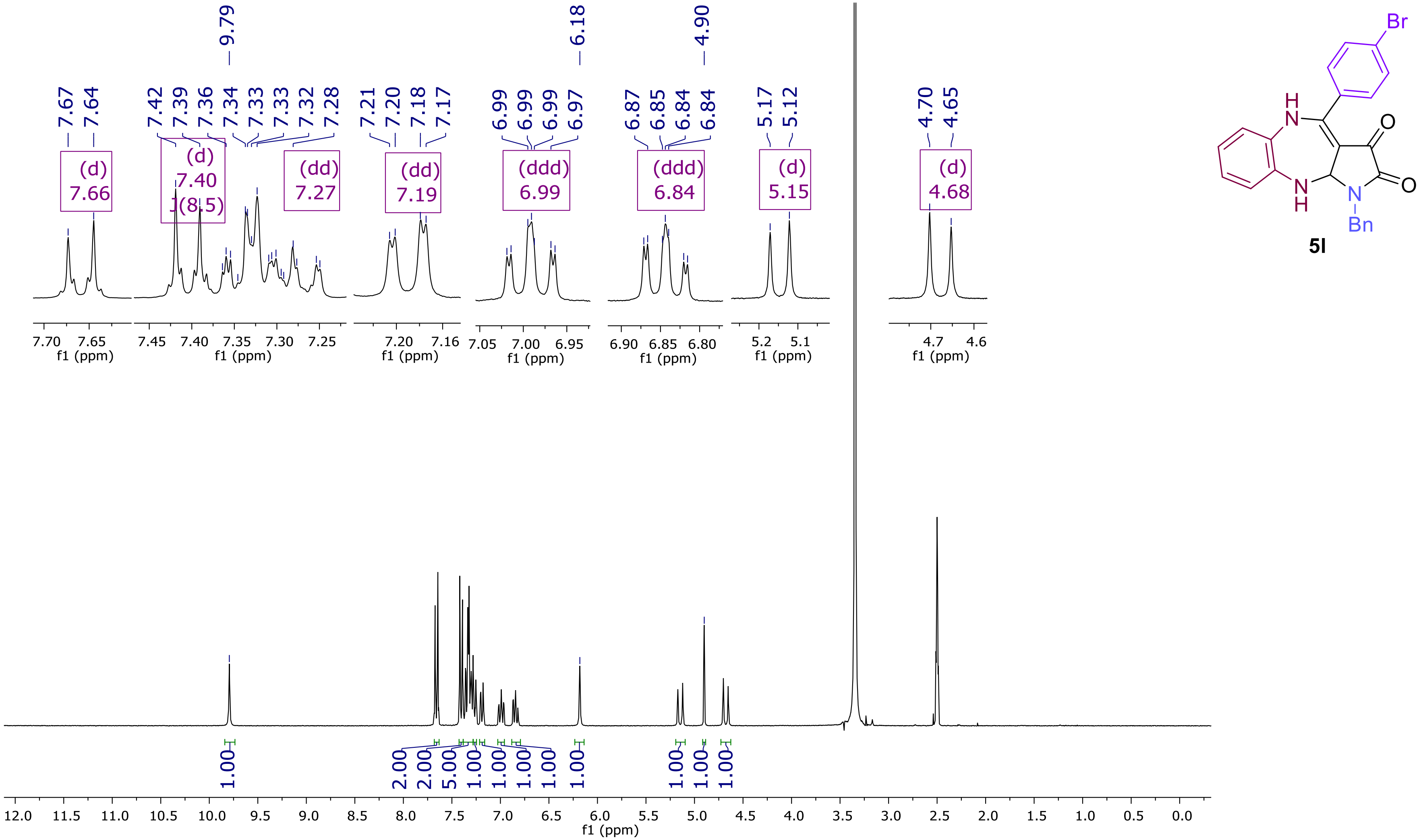

Figure S28. ^{1}H NMR spectrum of **5l** (DMSO-d_6, 300.06 MHz)

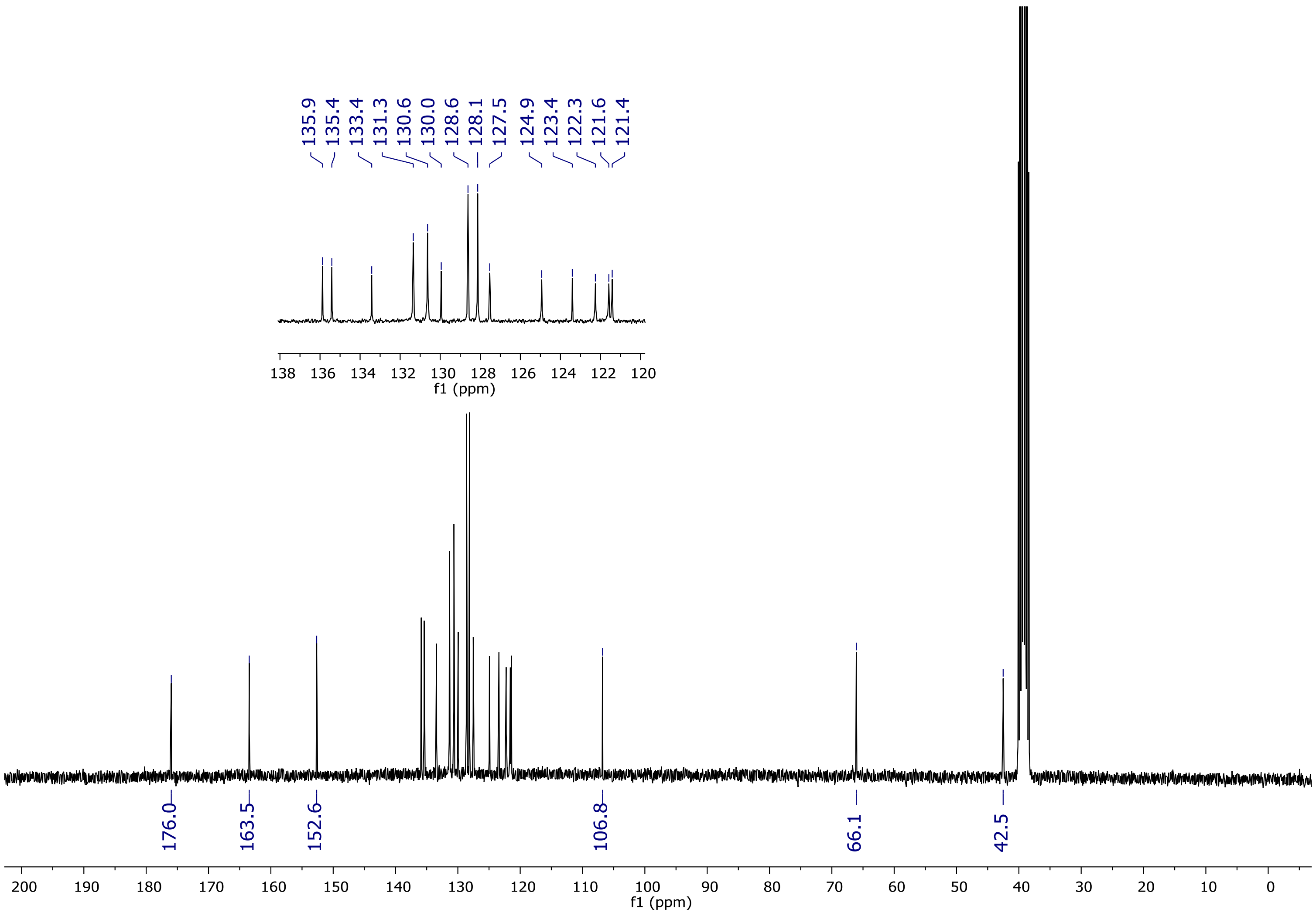

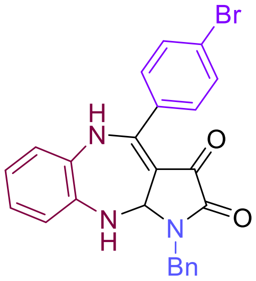

5l

Figure S29. $^{13}C\{^1H\}$ NMR spectrum of **5l** (DMSO-d_6, 75.46 MHz)

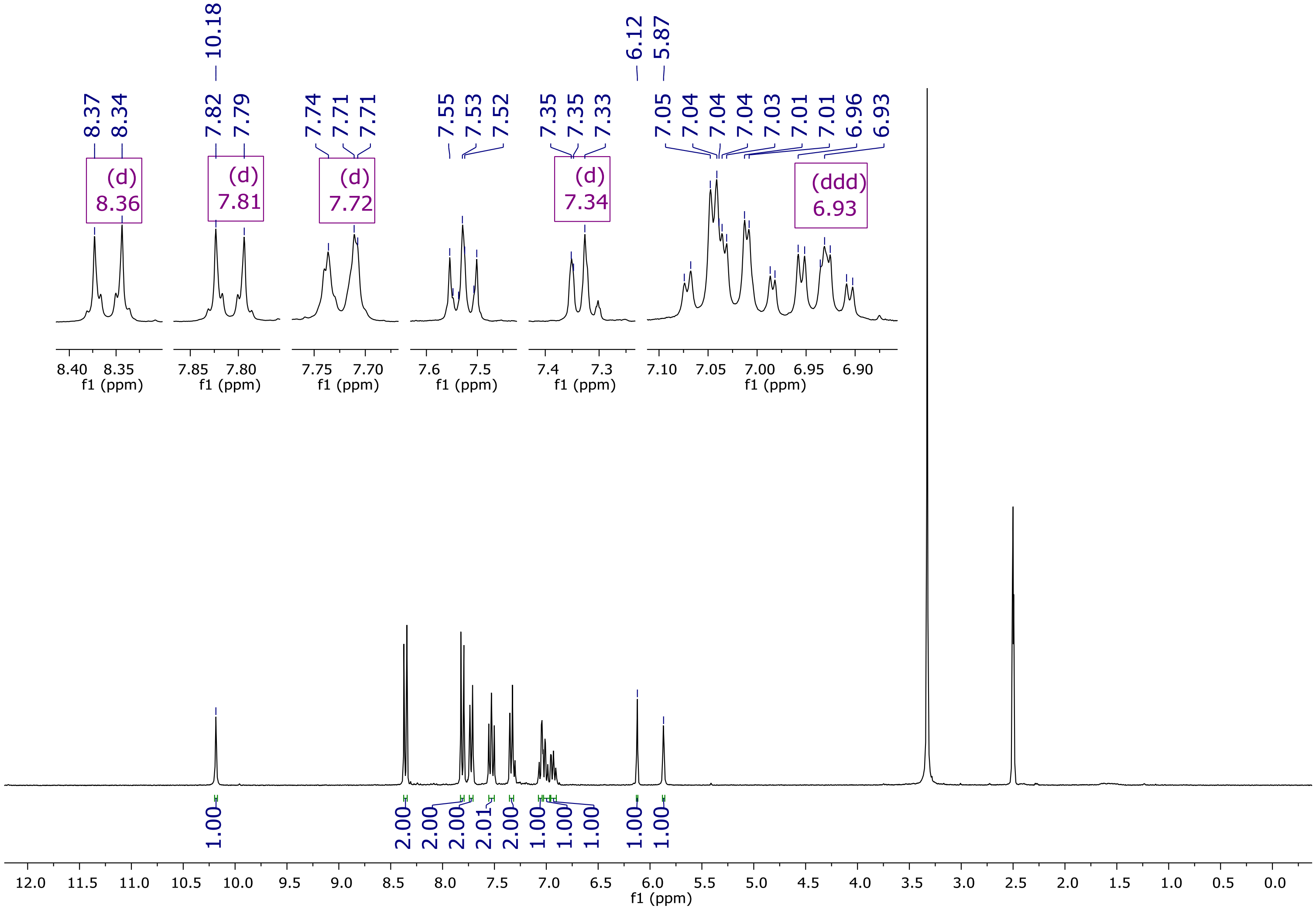

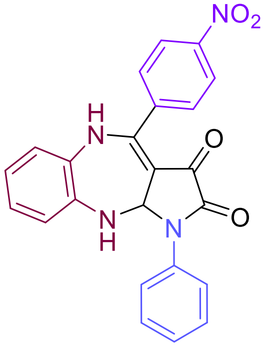

5m

Figure S30. ^{1}H NMR spectrum of **5m** (DMSO-d_6, 300.06 MHz)

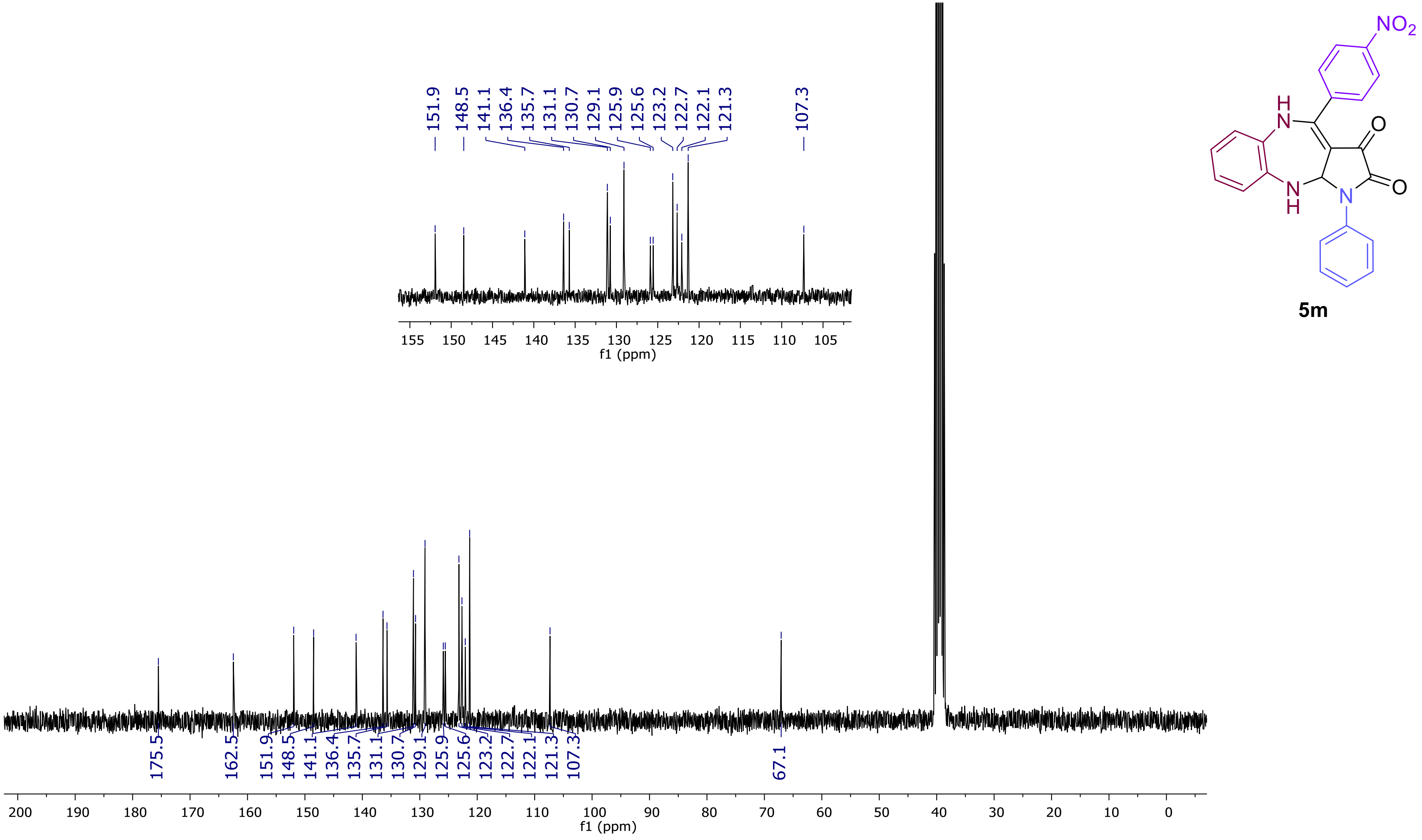

Figure S31. $^{13}C\{^{1}H\}$ NMR spectrum of **5m** (DMSO-d_6, 75.46 MHz)

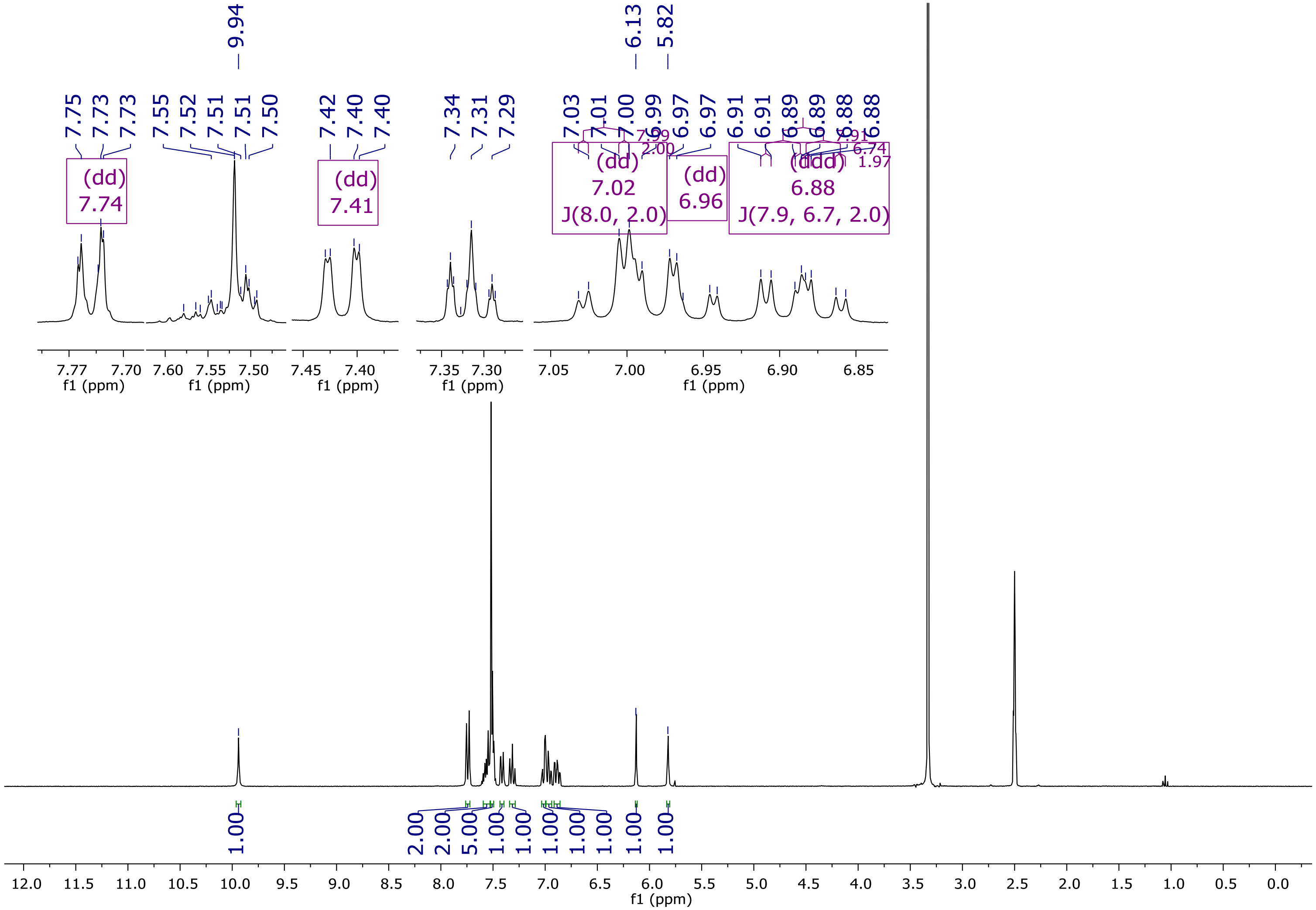

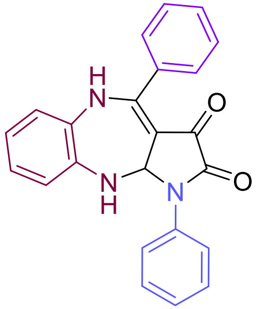

5n

Figure S32. ^{1}H NMR spectrum of **5n** (DMSO-d_6, 300.06 MHz)

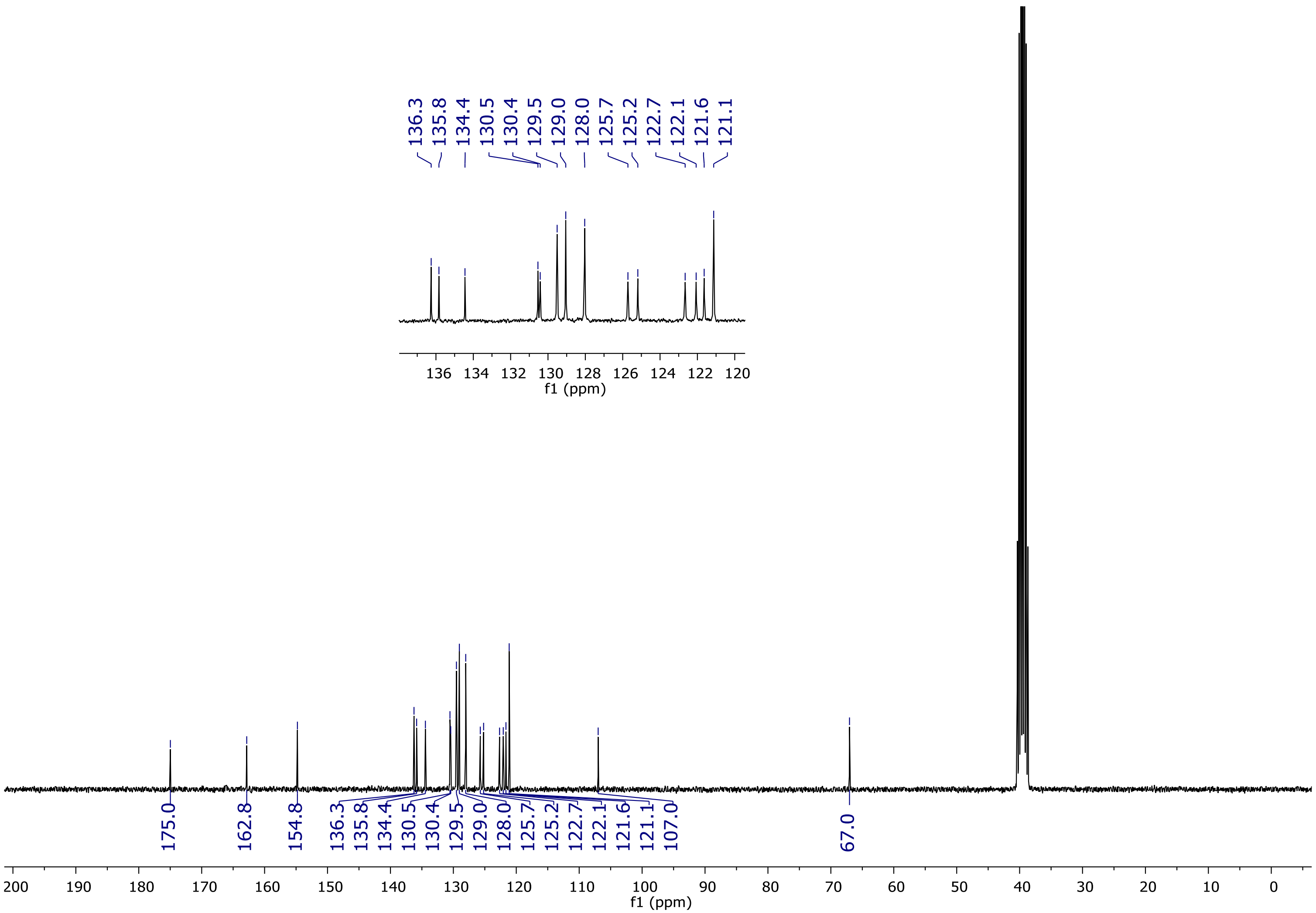

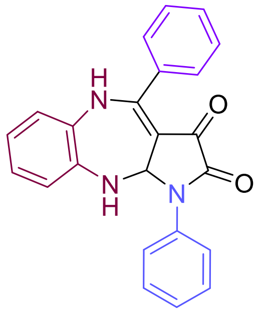

5n

Figure S33. $^{13}C\{^{1}H\}$ NMR spectrum of **5n** (DMSO-d_6, 75.46 MHz)

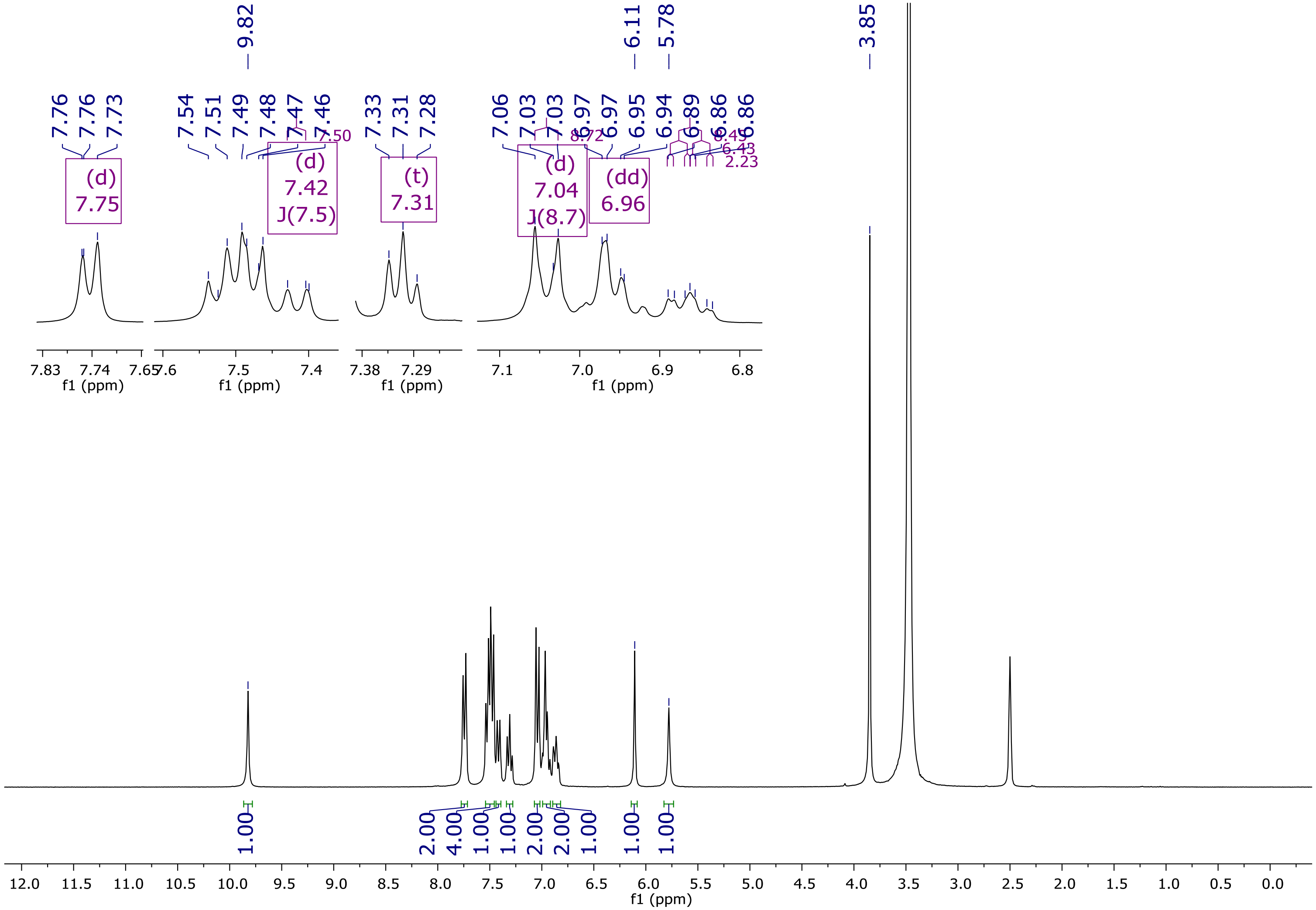

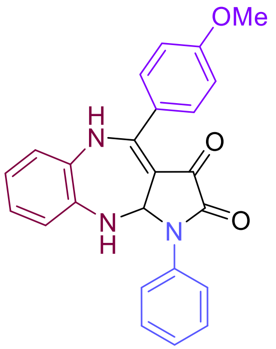

5o

Figure S34. ^{1}H NMR spectrum of **5o** (DMSO-d_6, 300.06 MHz)

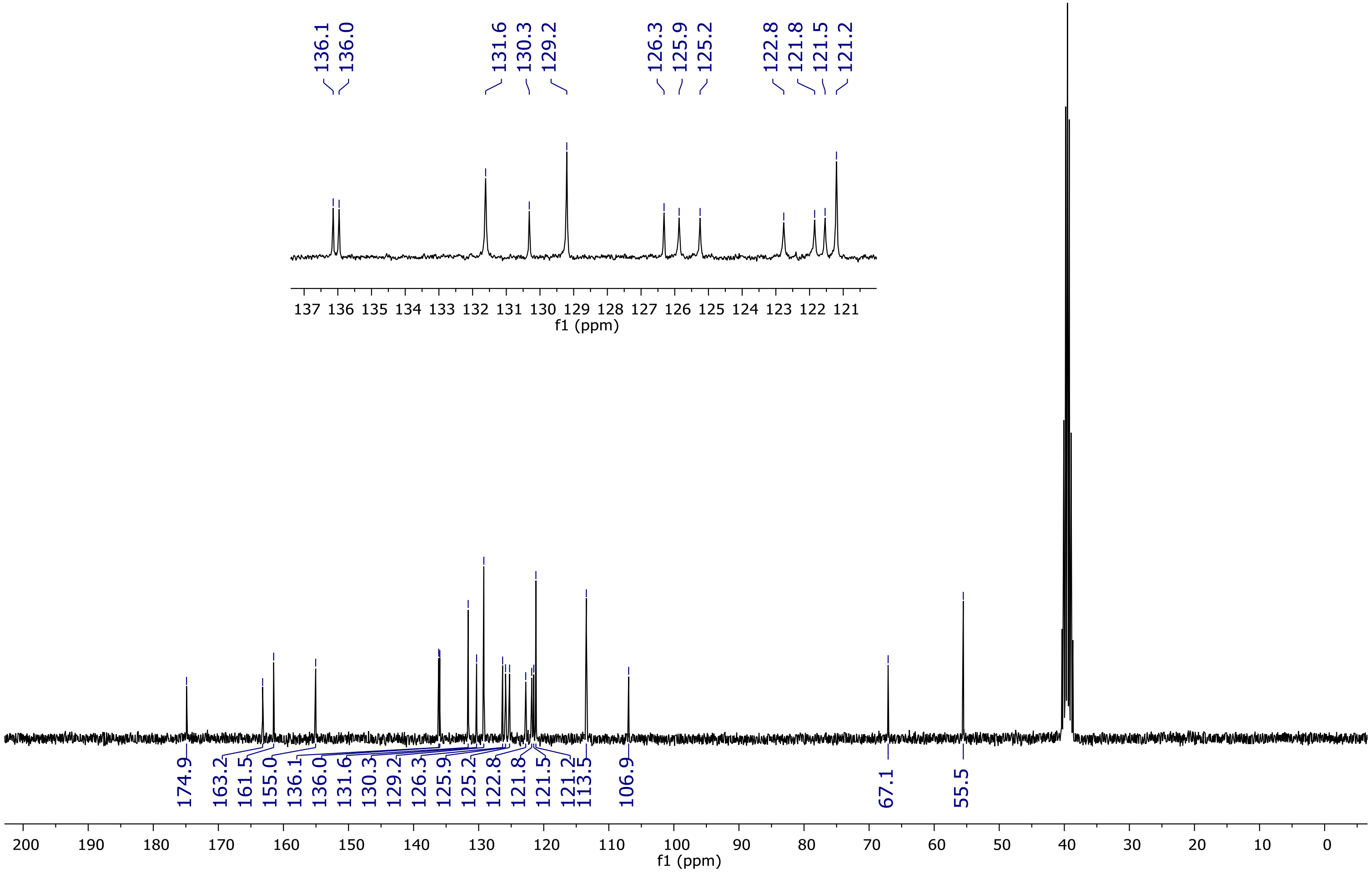

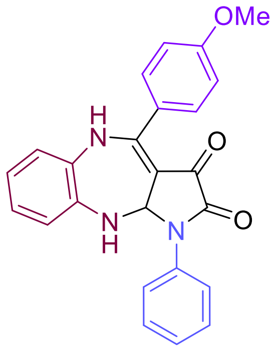

5o

Figure S35. $^{13}C\{^1H\}$ NMR spectrum of **5o** (DMSO-d_6, 75.46 MHz)

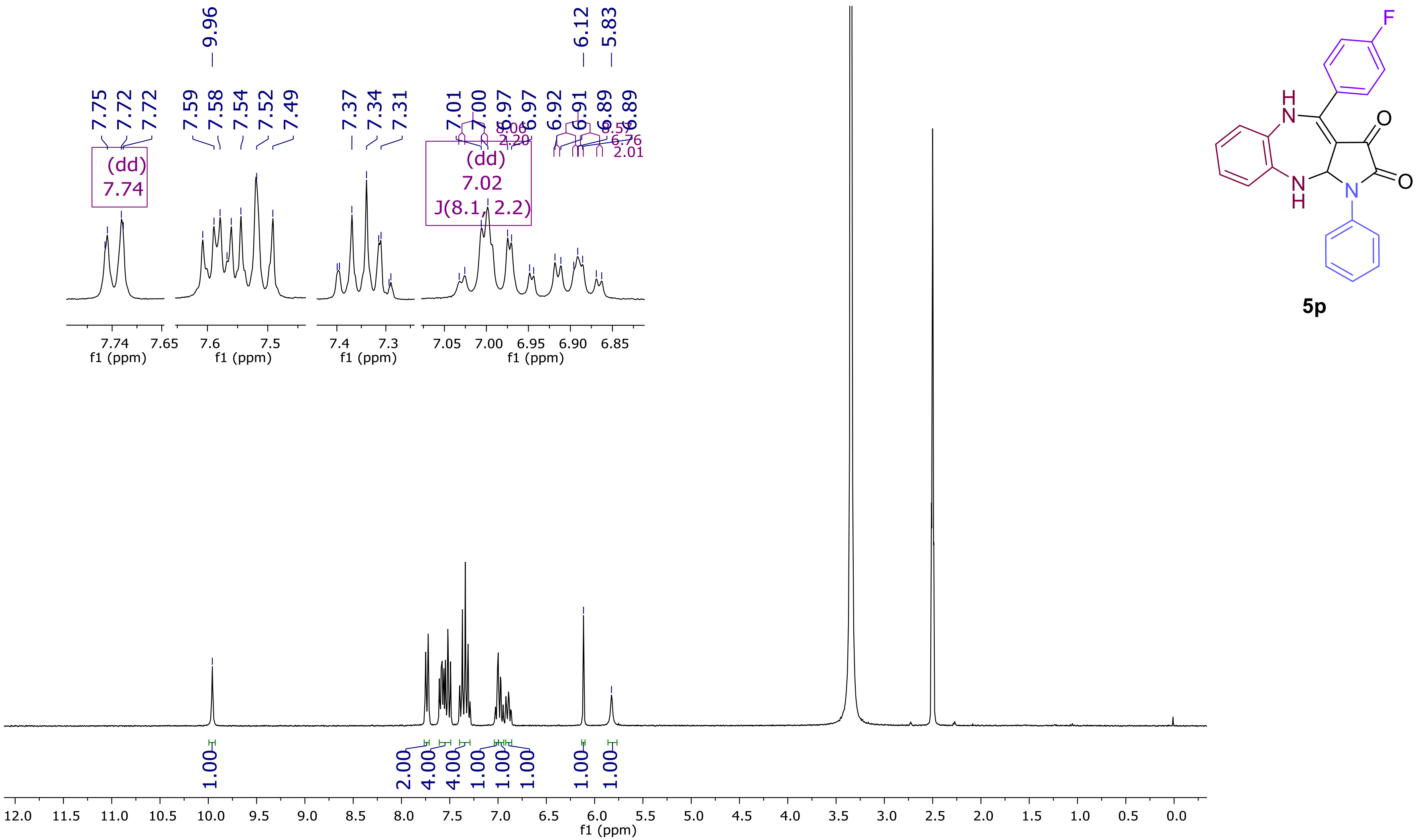

5p

Figure S36. ^{1}H NMR spectrum of **5p** (DMSO-d_6, 300.06 MHz)

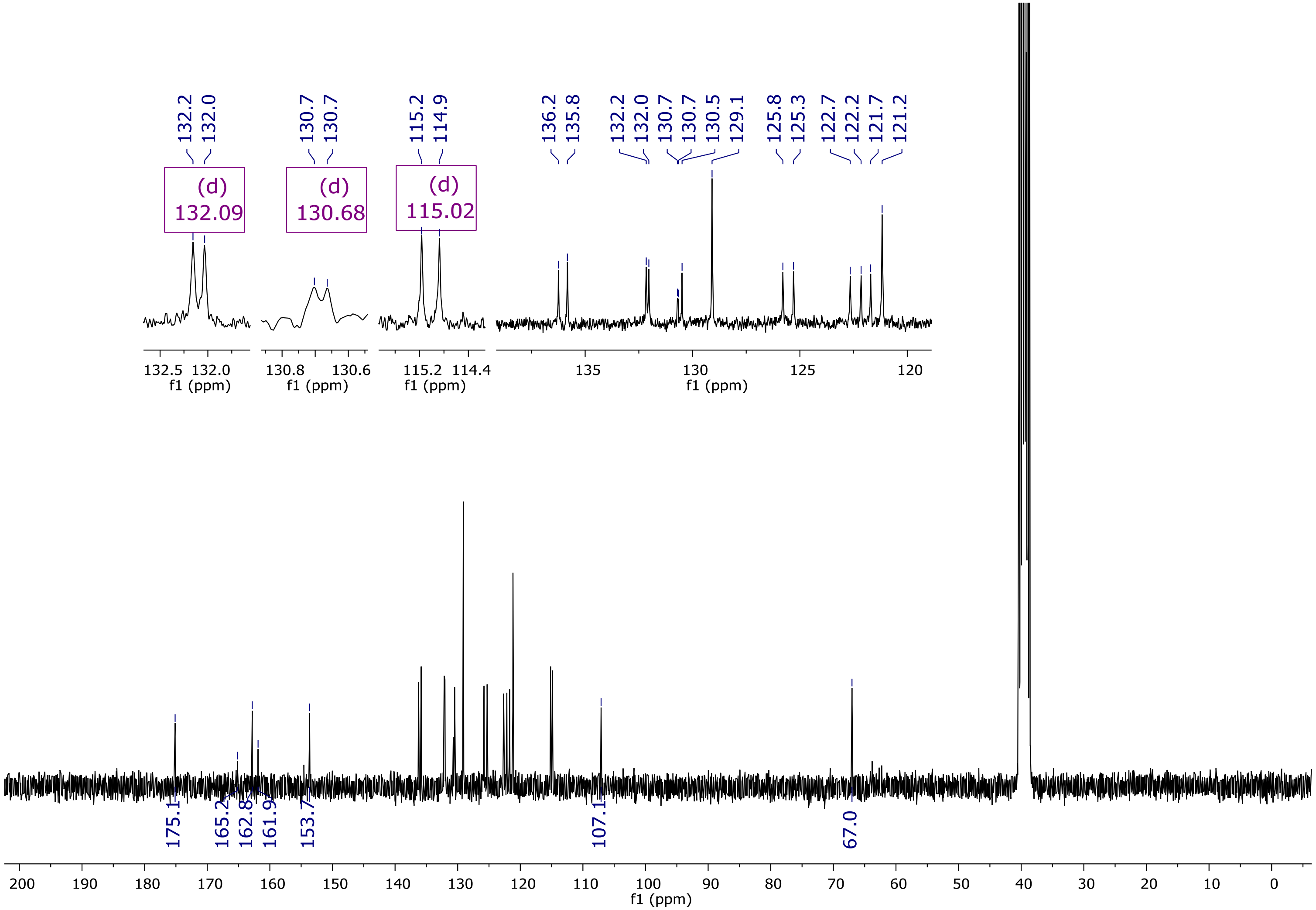

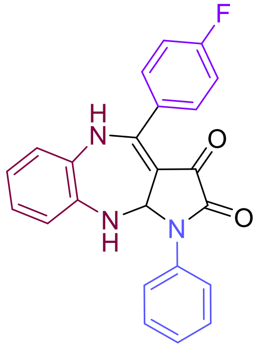

5p

Figure S37. $^{13}C\{^{1}H\}$ NMR spectrum of **5p** (DMSO-d_6, 75.46 MHz)

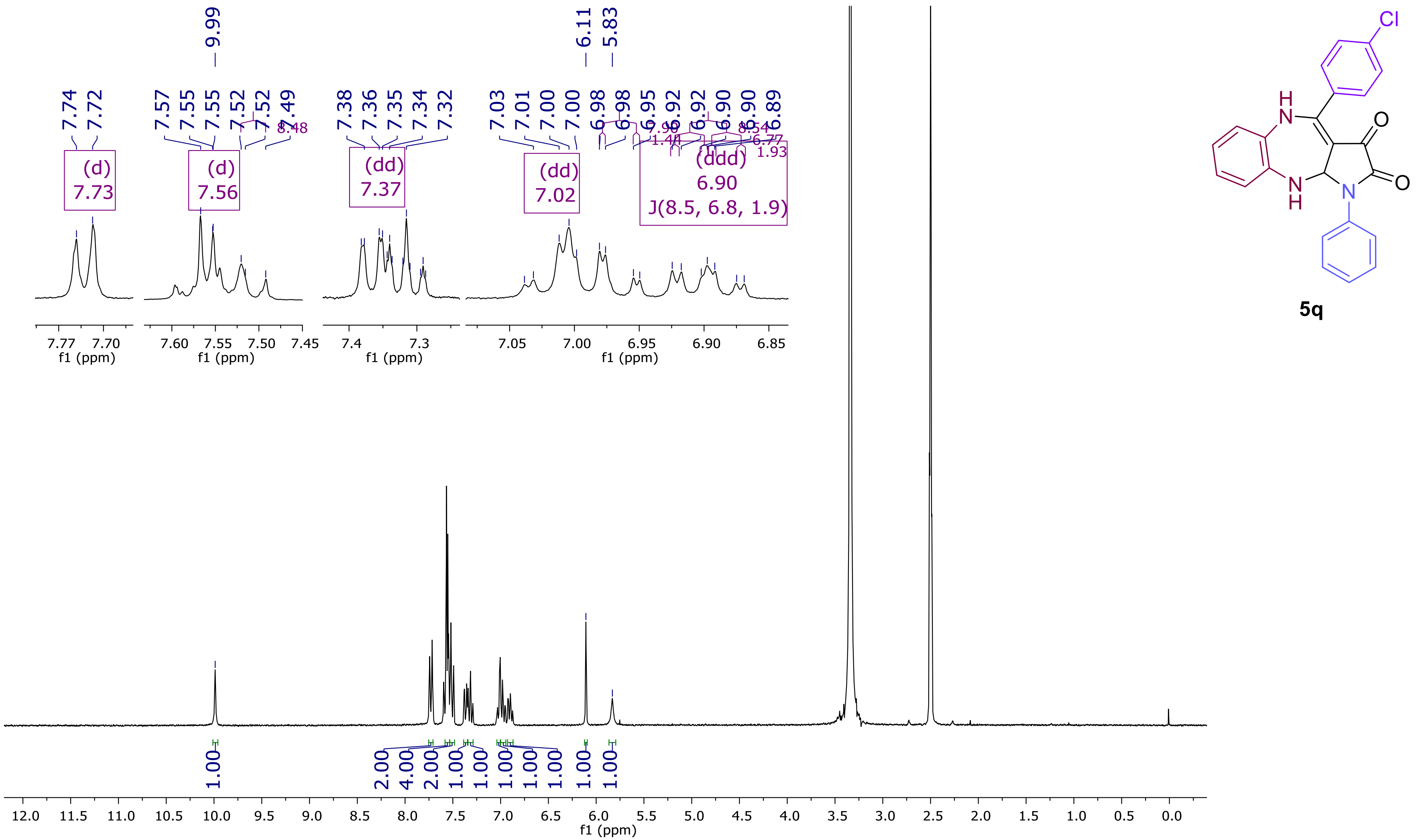

Figure S38. ^{1}H NMR spectrum of **5q** (DMSO-d_6, 300.06 MHz)

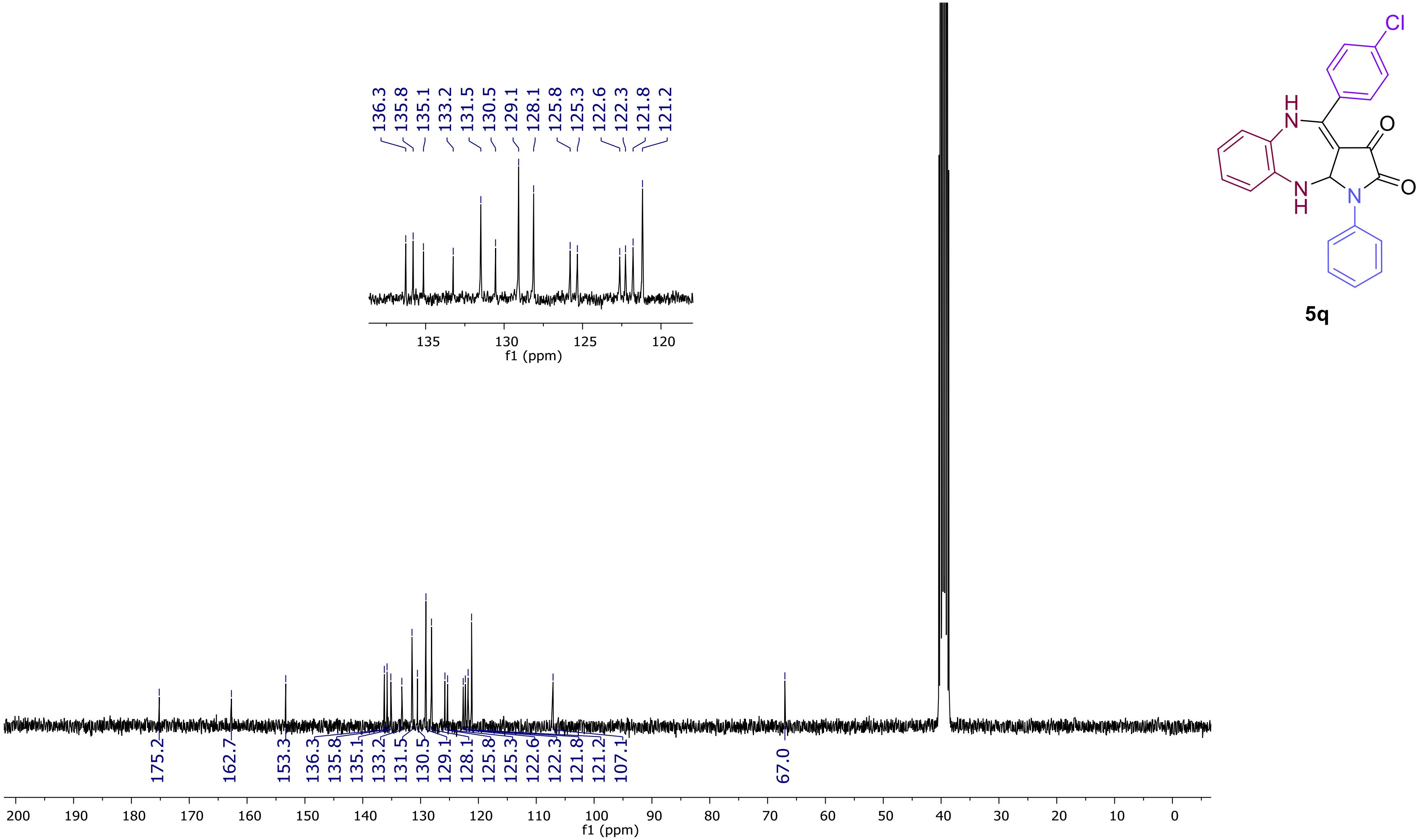

Figure S39. $^{13}C\{^{1}H\}$ NMR spectrum of **5q** (DMSO-d_6, 75.46 MHz)

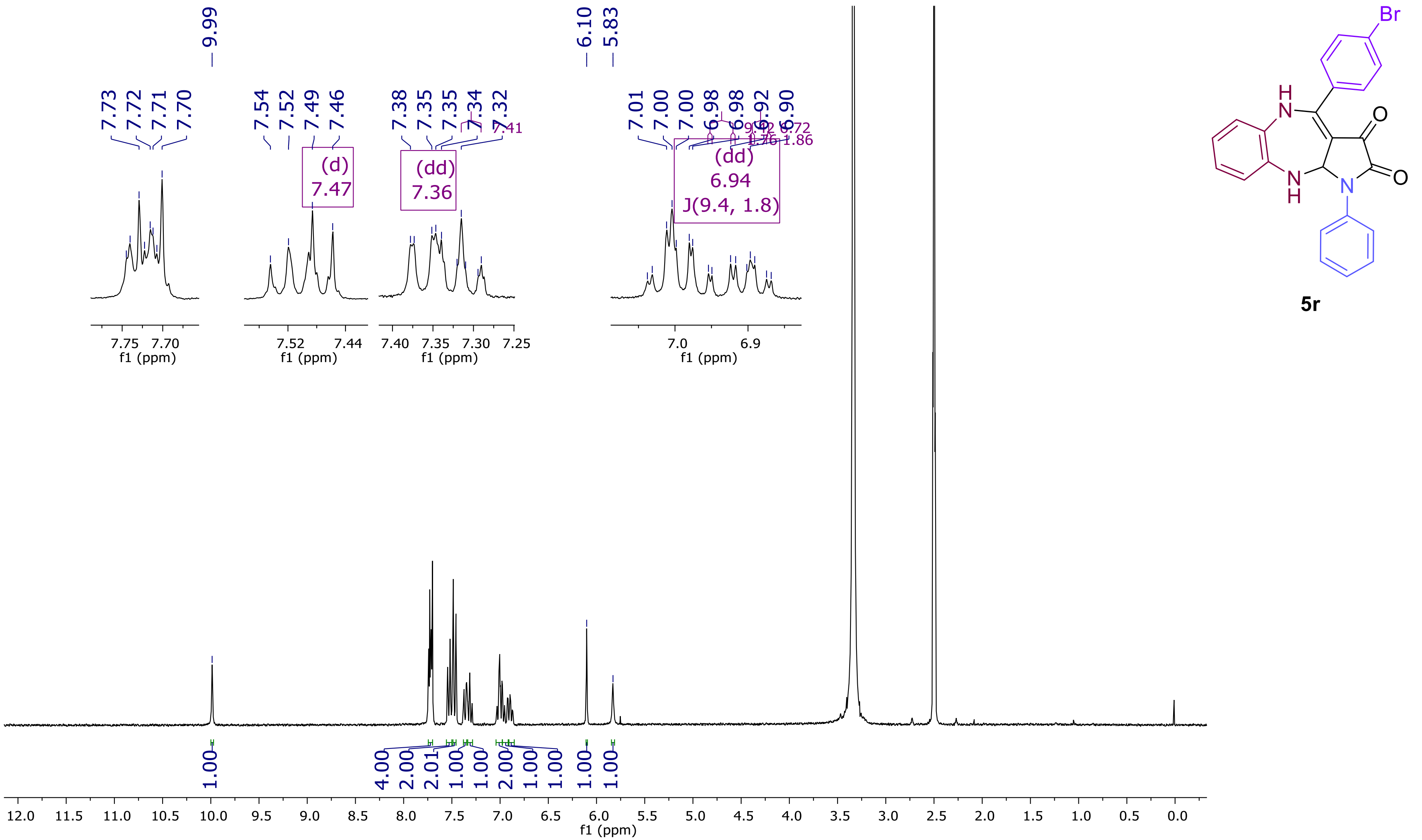

Figure S40. ^{1}H NMR spectrum of **5r** (DMSO-d_6, 300.06 MHz)

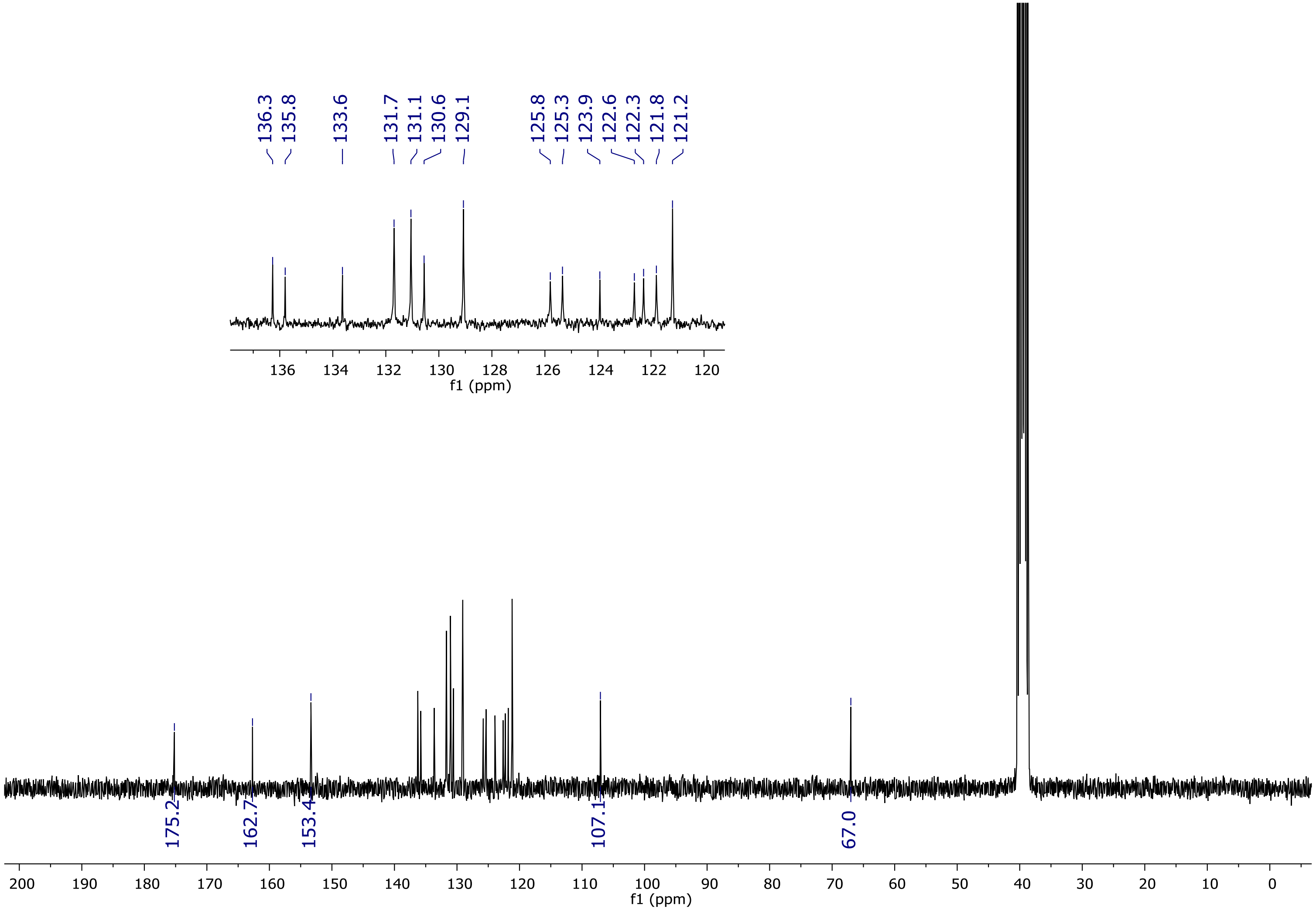

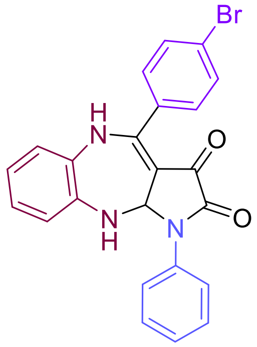

5r

Figure S41. $^{13}C\{^1H\}$ NMR spectrum of **5r** (DMSO-d_6, 75.46 MHz)

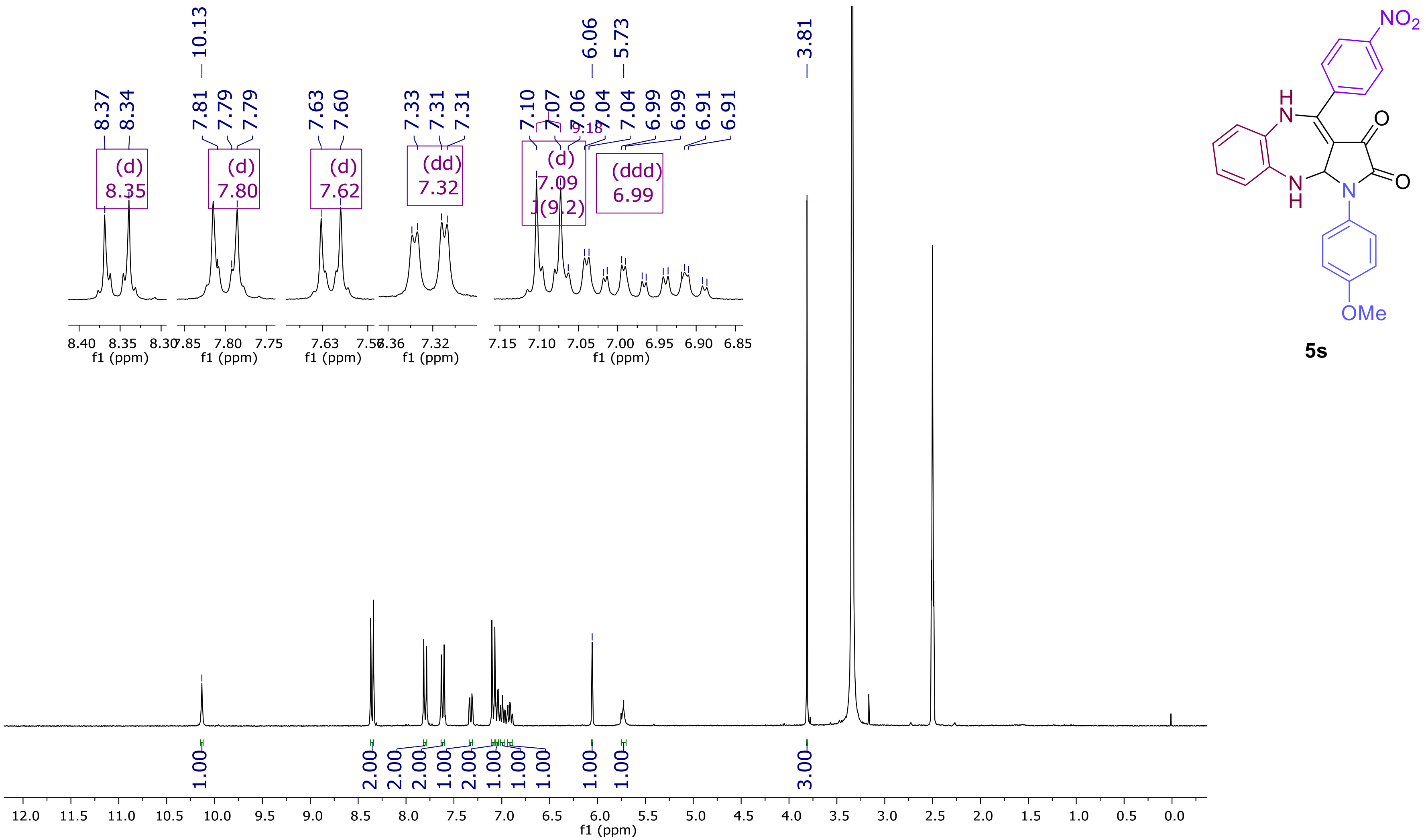

Figure S42. ^{1}H NMR spectrum of **5s** (DMSO-d_6, 300.06 MHz)

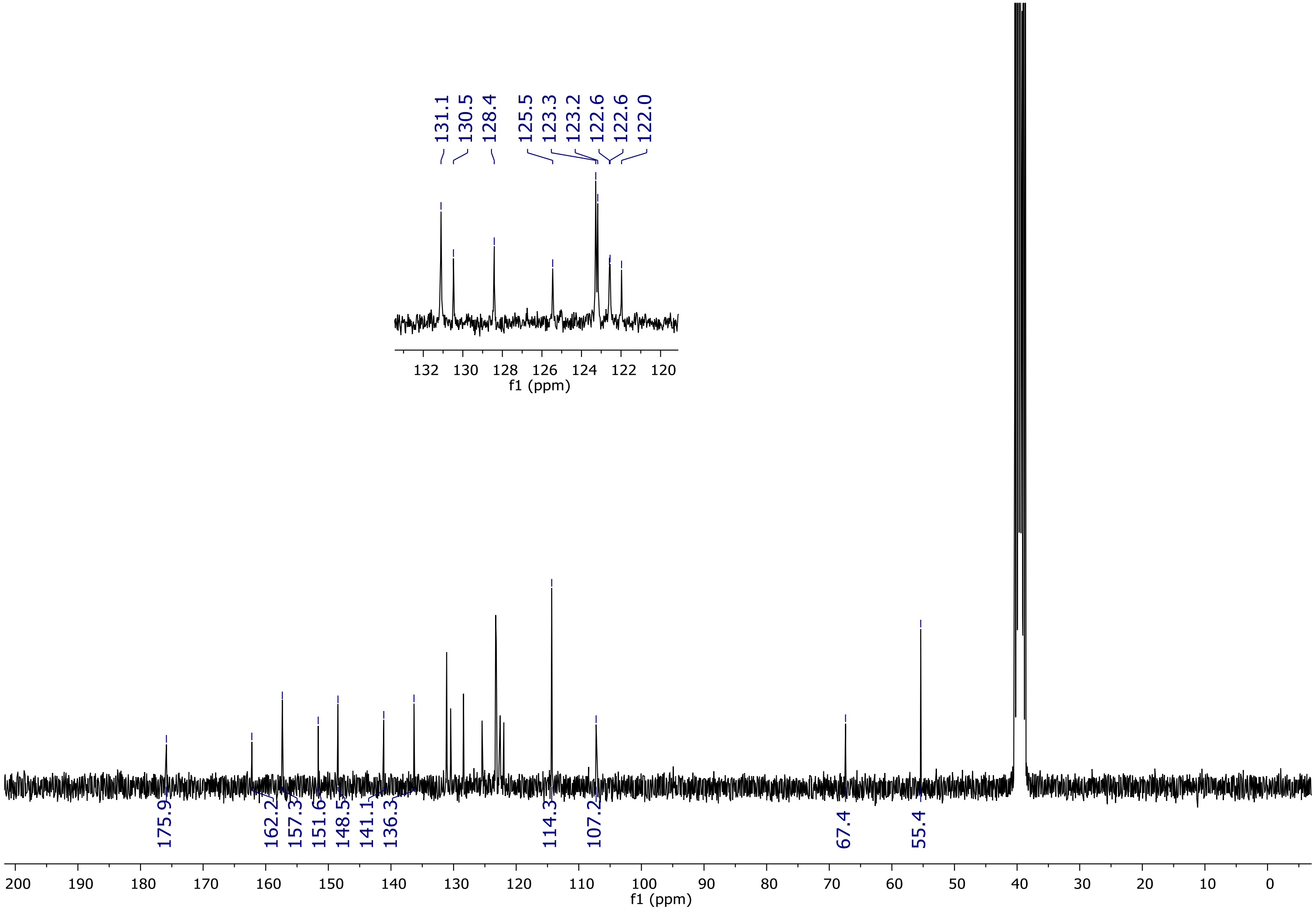

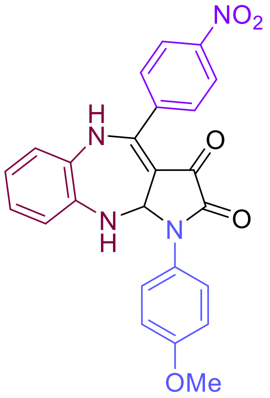

5s

Figure S43. $^{13}C\{^{1}H\}$ NMR spectrum of **5s** (DMSO-d_6, 75.46 MHz)

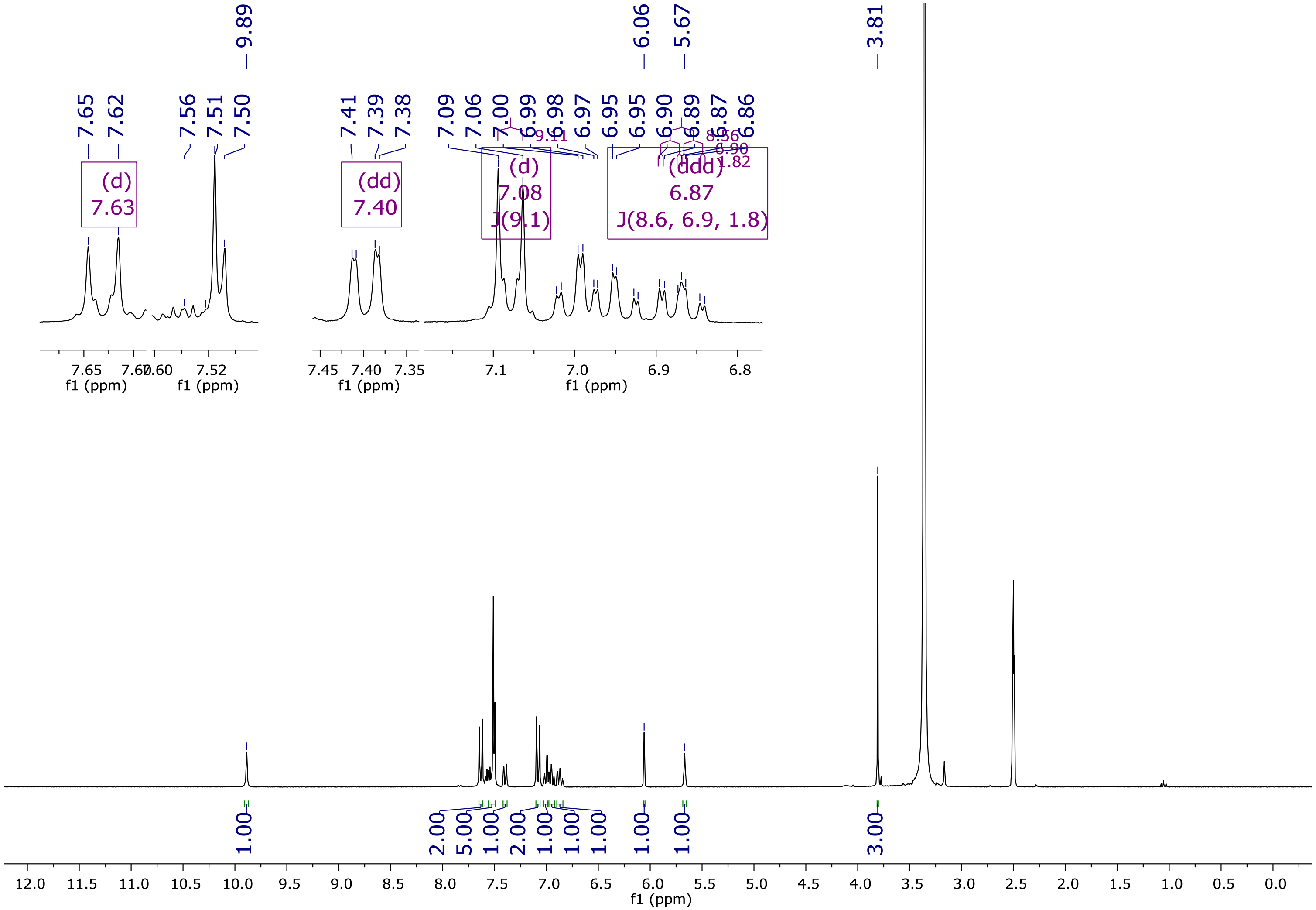

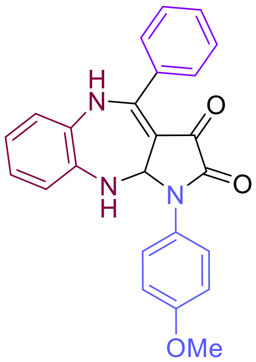

5t

Figure S44. ^{1}H NMR spectrum of **5t** (DMSO-d_6, 300.06 MHz)

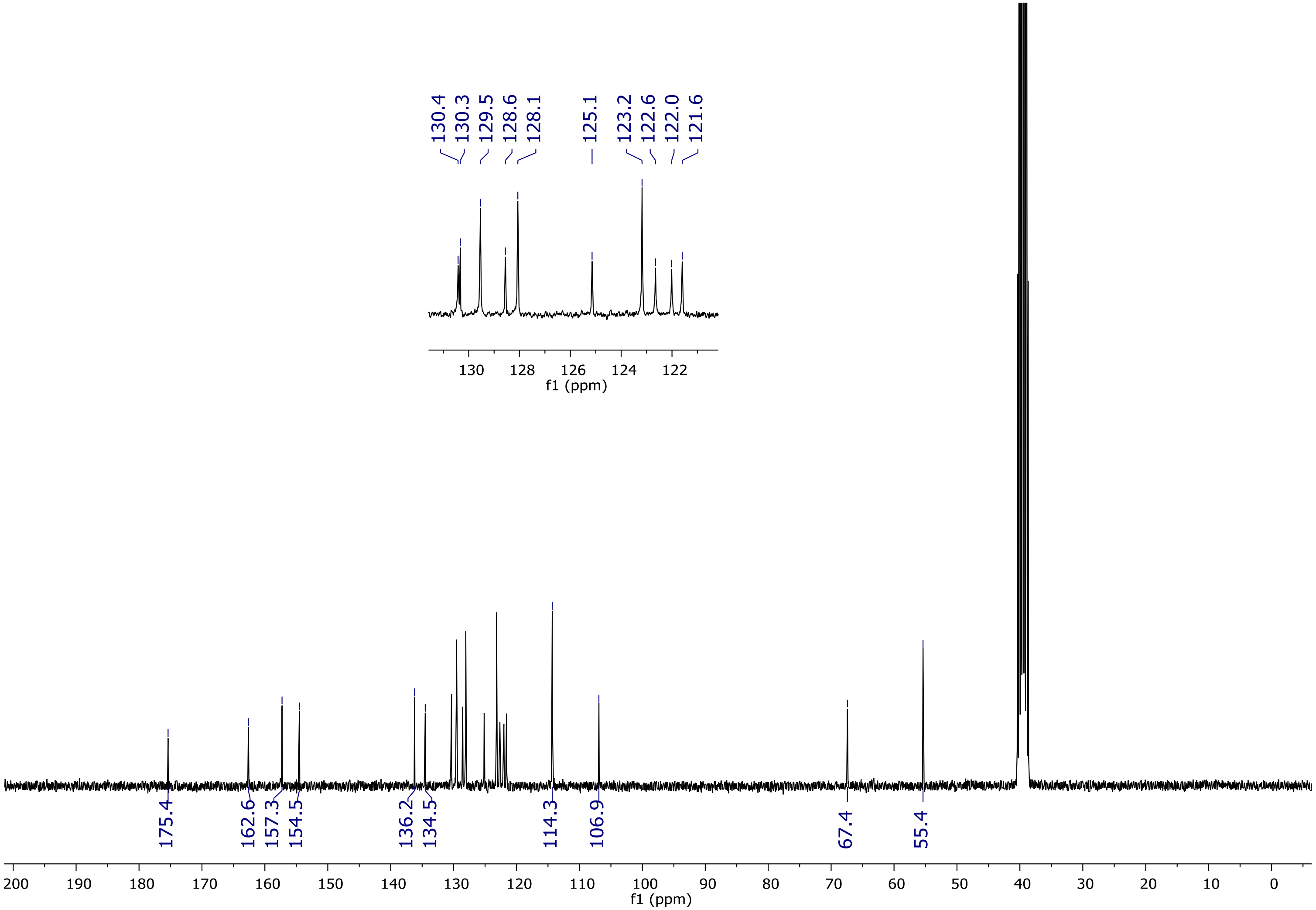

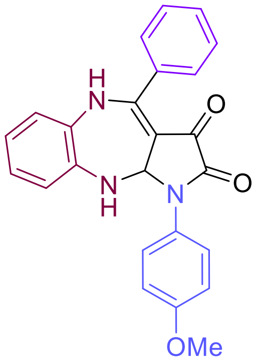

5t

Figure S45. $^{13}C\{^{1}H\}$ NMR spectrum of **5t** (DMSO-d_6, 75.46 MHz)

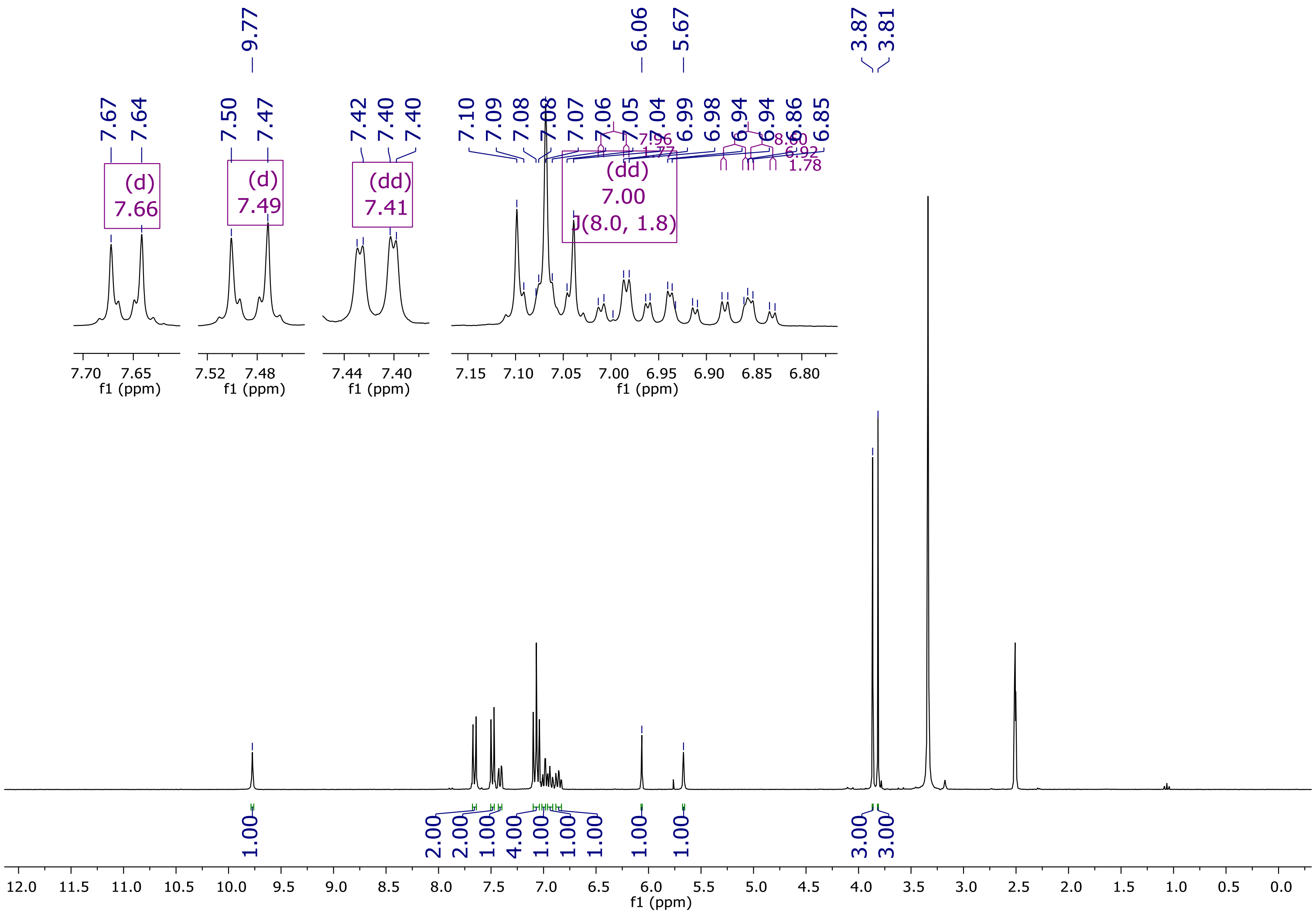

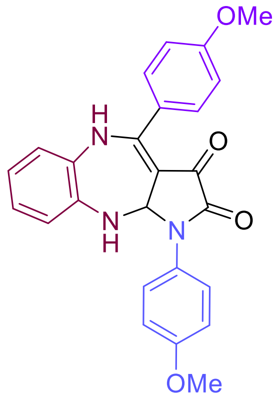

5u

Figure S46. ^{1}H NMR spectrum of **5u** (DMSO-d_6, 300.06 MHz)

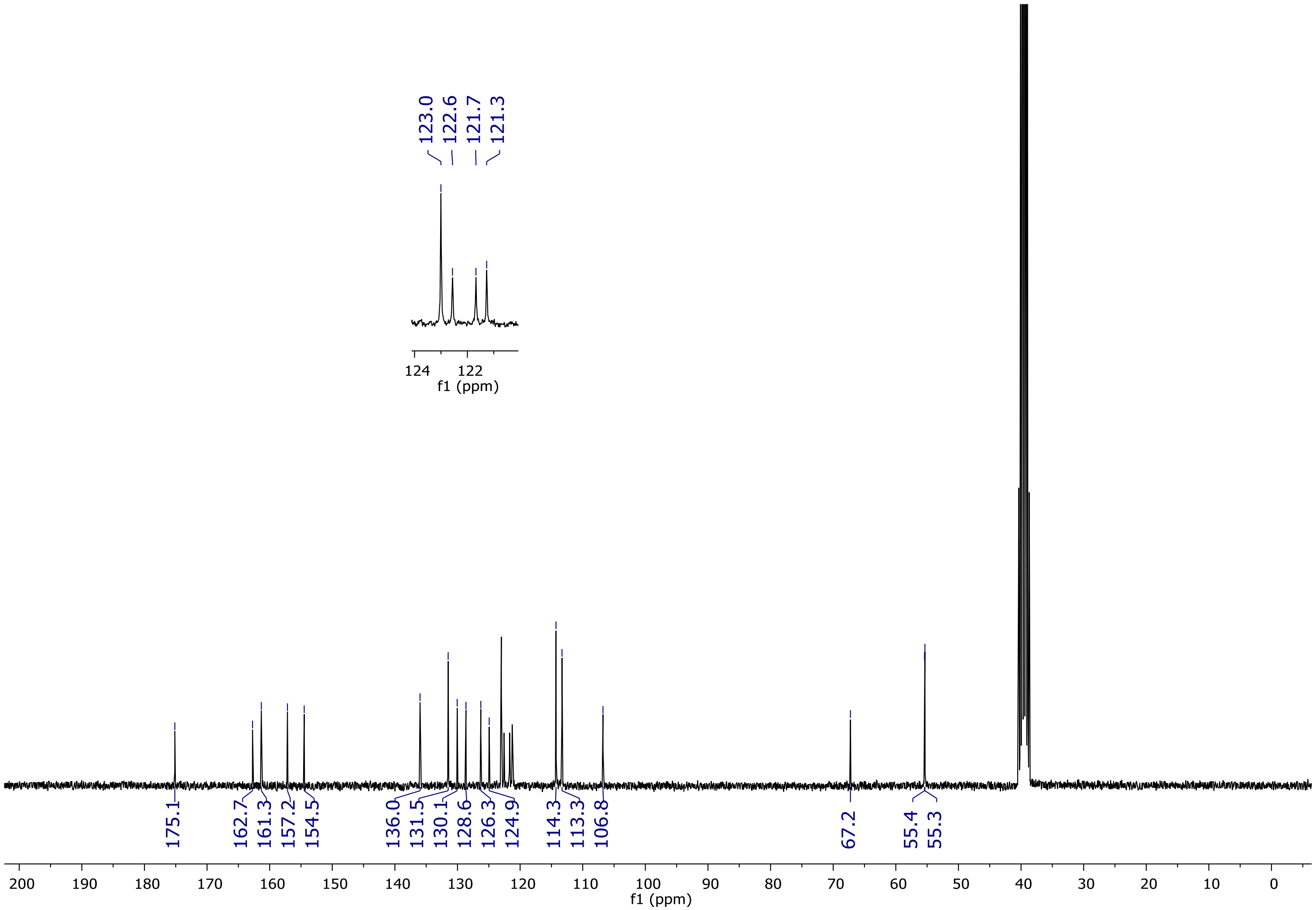

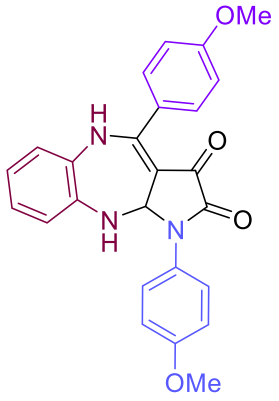

5u

Figure S47. $^{13}C\{^1H\}$ NMR spectrum of **5u** (DMSO-d_6, 75.46 MHz)

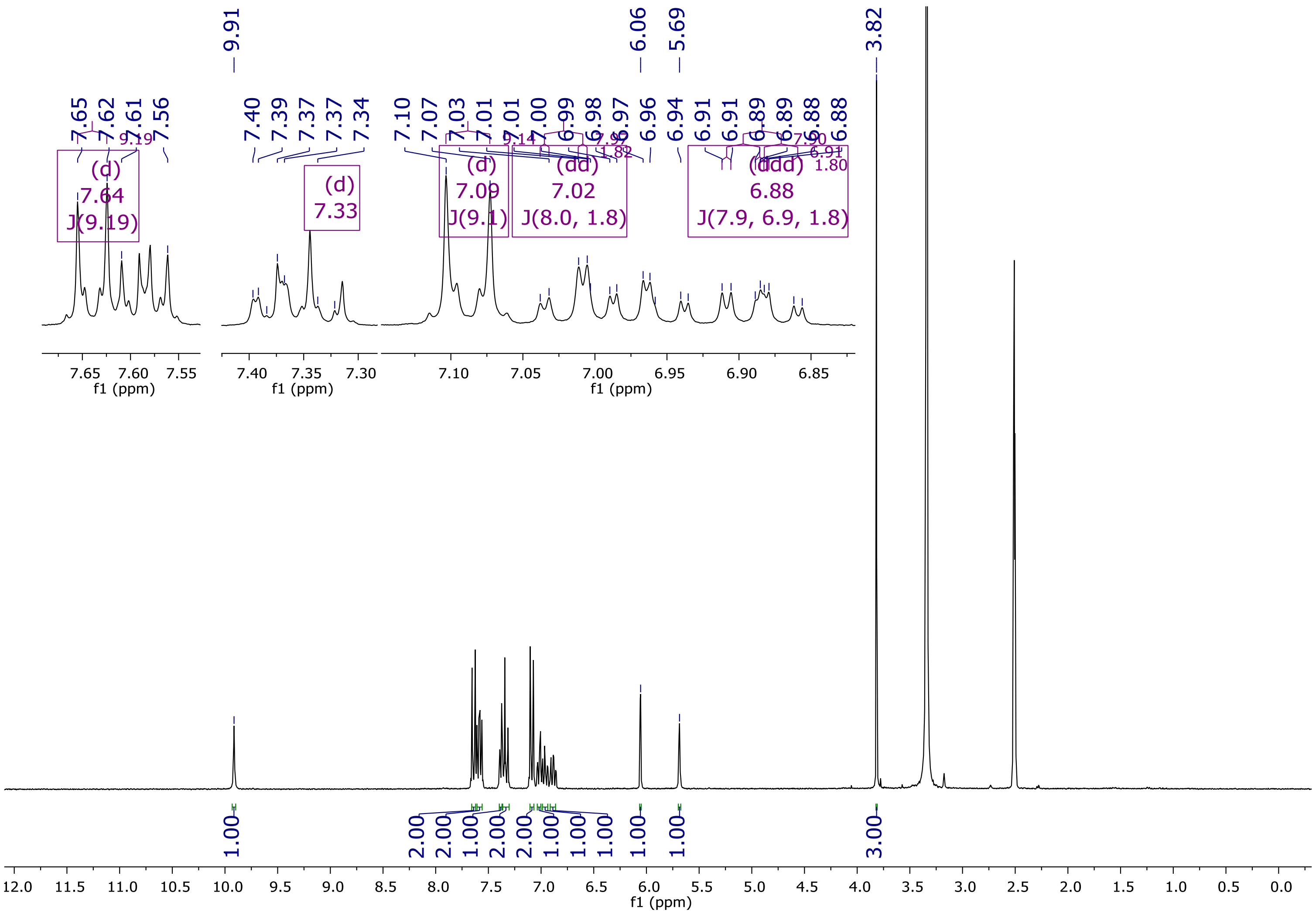

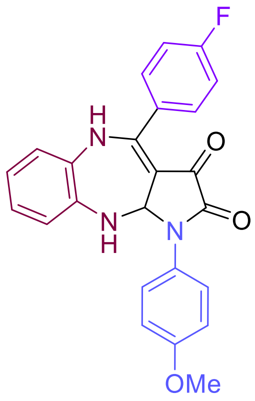

5v

Figure S48. ^{1}H NMR spectrum of **5v** (DMSO-d_6, 300.06 MHz)

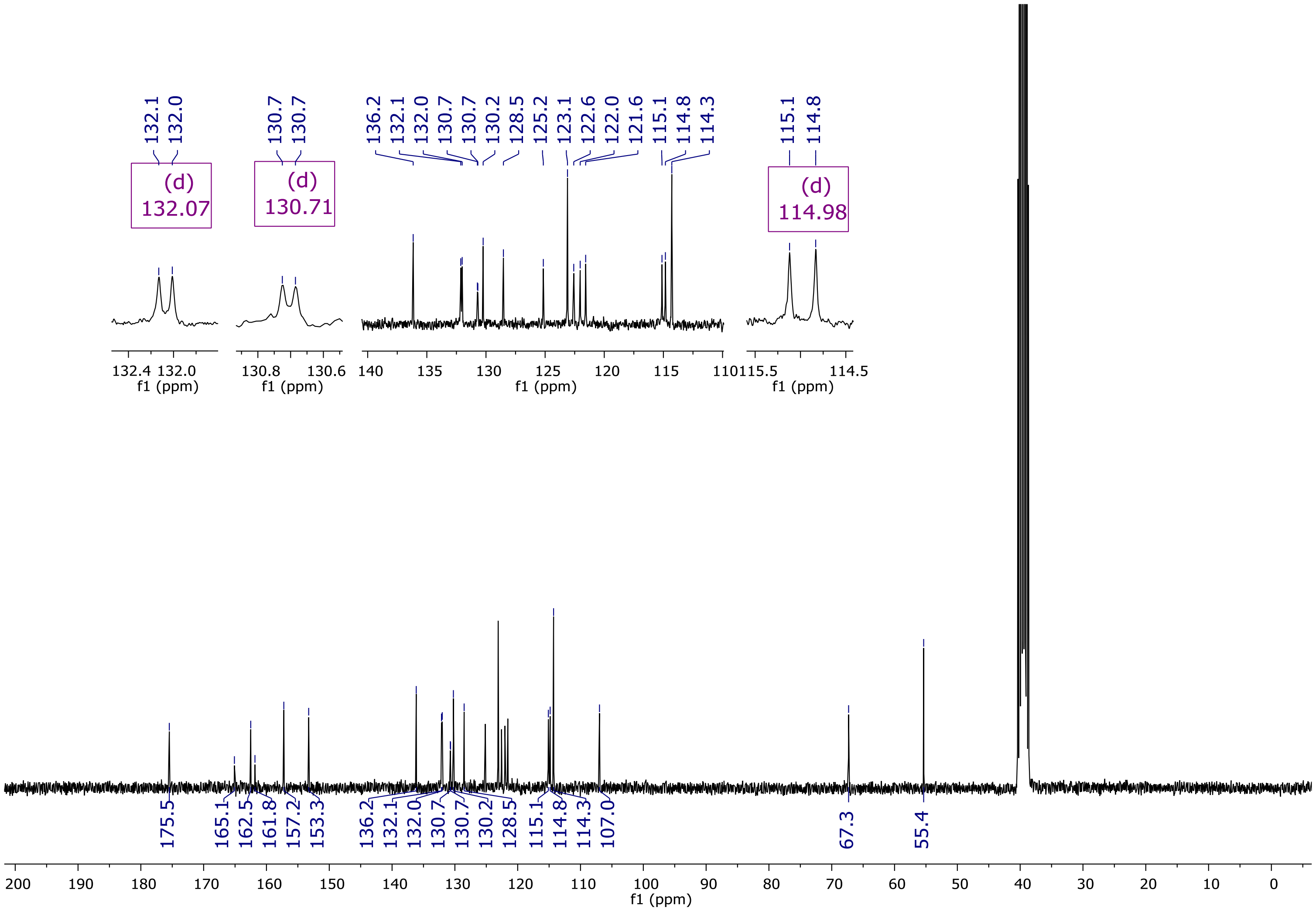

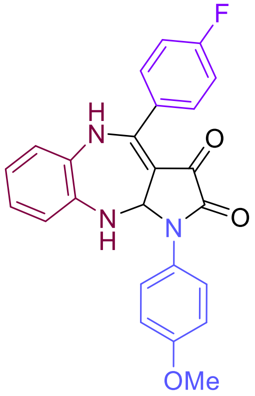

5v

Figure S49. $^{13}C\{^{1}H\}$ NMR spectrum of **5v** (DMSO-d_6, 75.46 MHz)

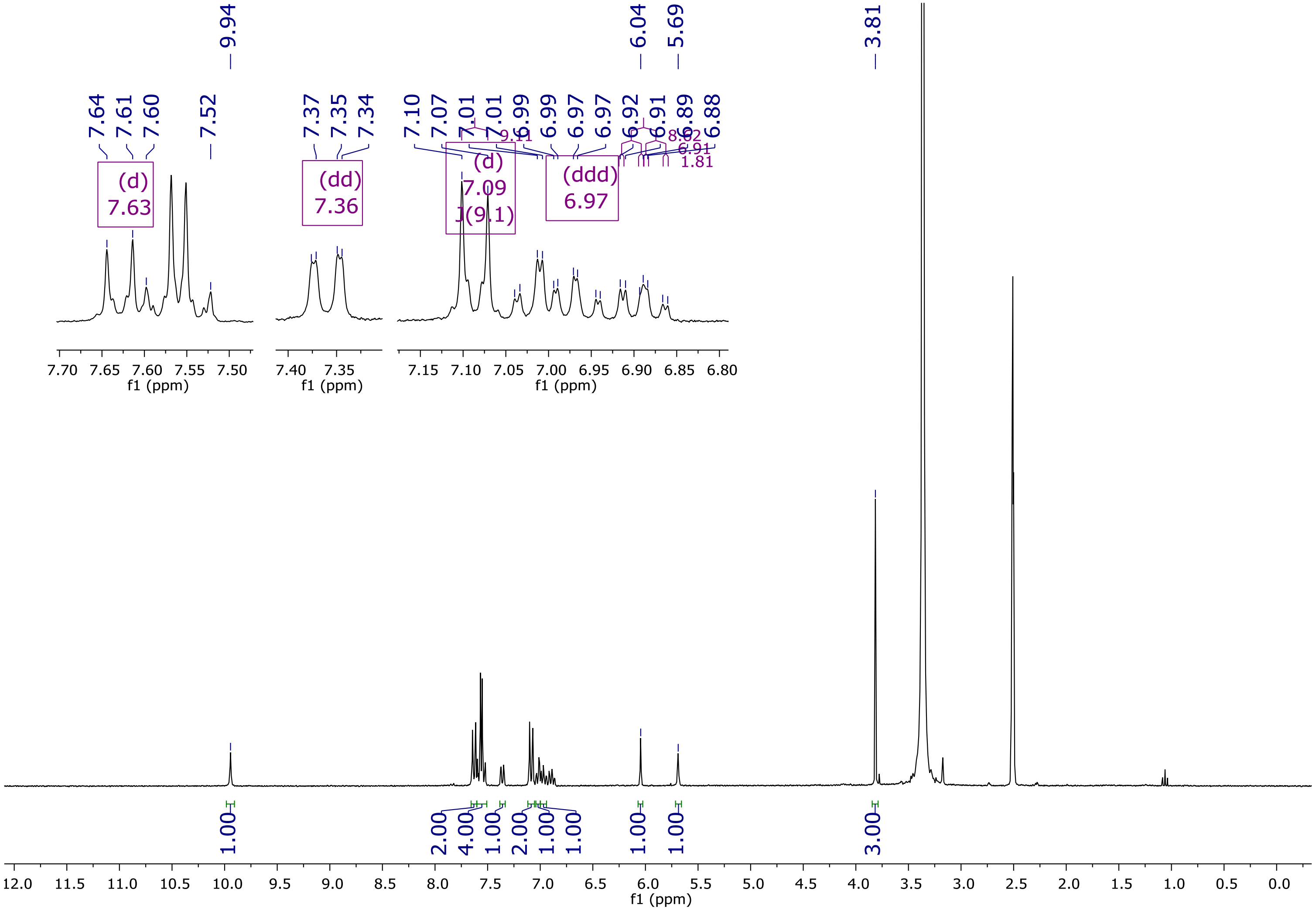

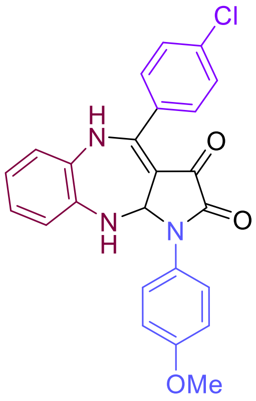

5w

Figure S50. ^{1}H NMR spectrum of **5w** (DMSO-d_6, 300.06 MHz)

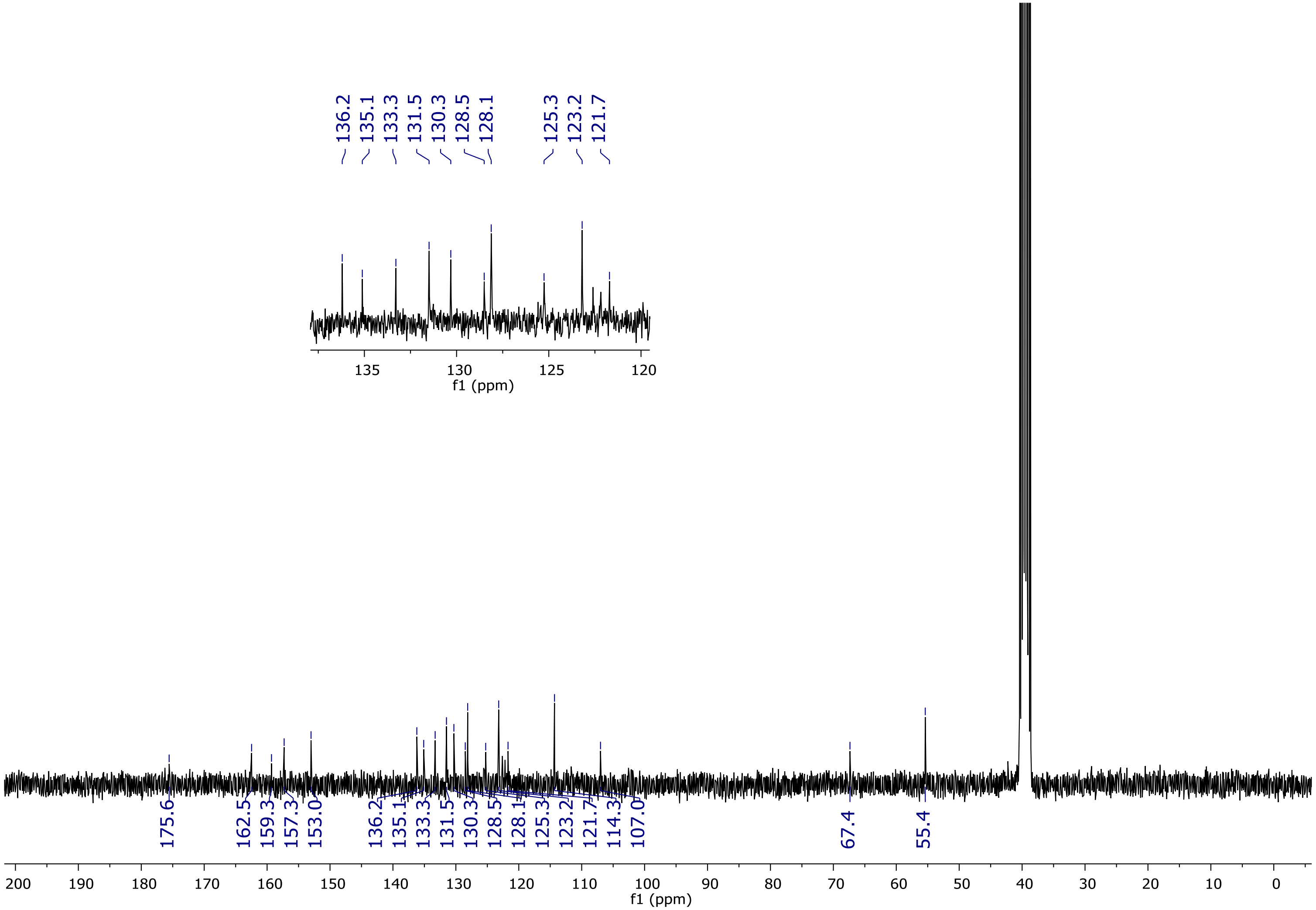

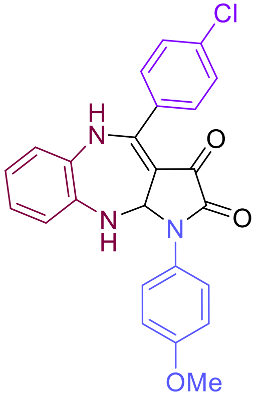

5w

Figure S51. $^{13}C\{^{1}H\}$ NMR spectrum of **5w** (DMSO-d_6, 75.46 MHz)

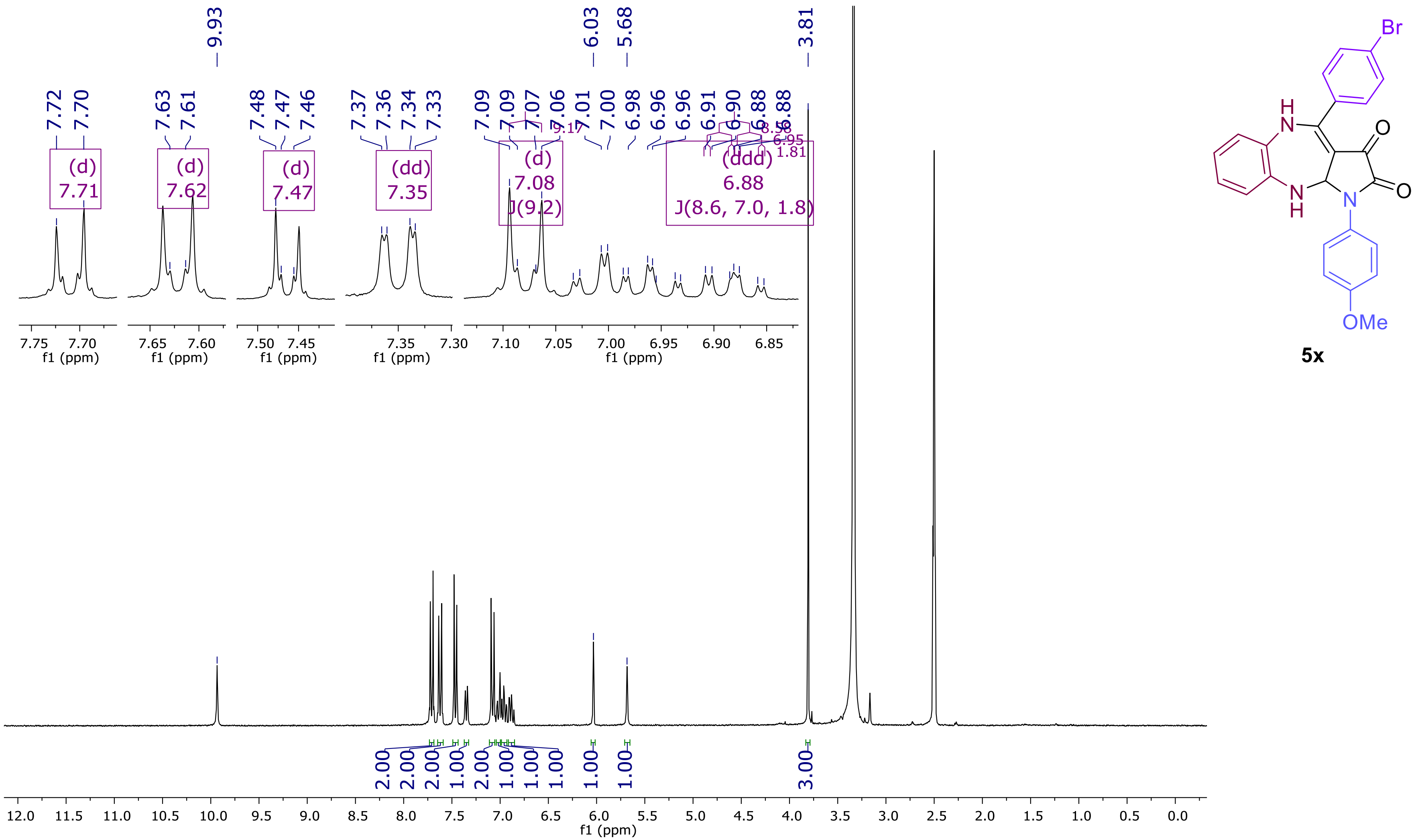

5x

Figure S52. ^{1}H NMR spectrum of **5x** (DMSO-d_6, 300.06 MHz)

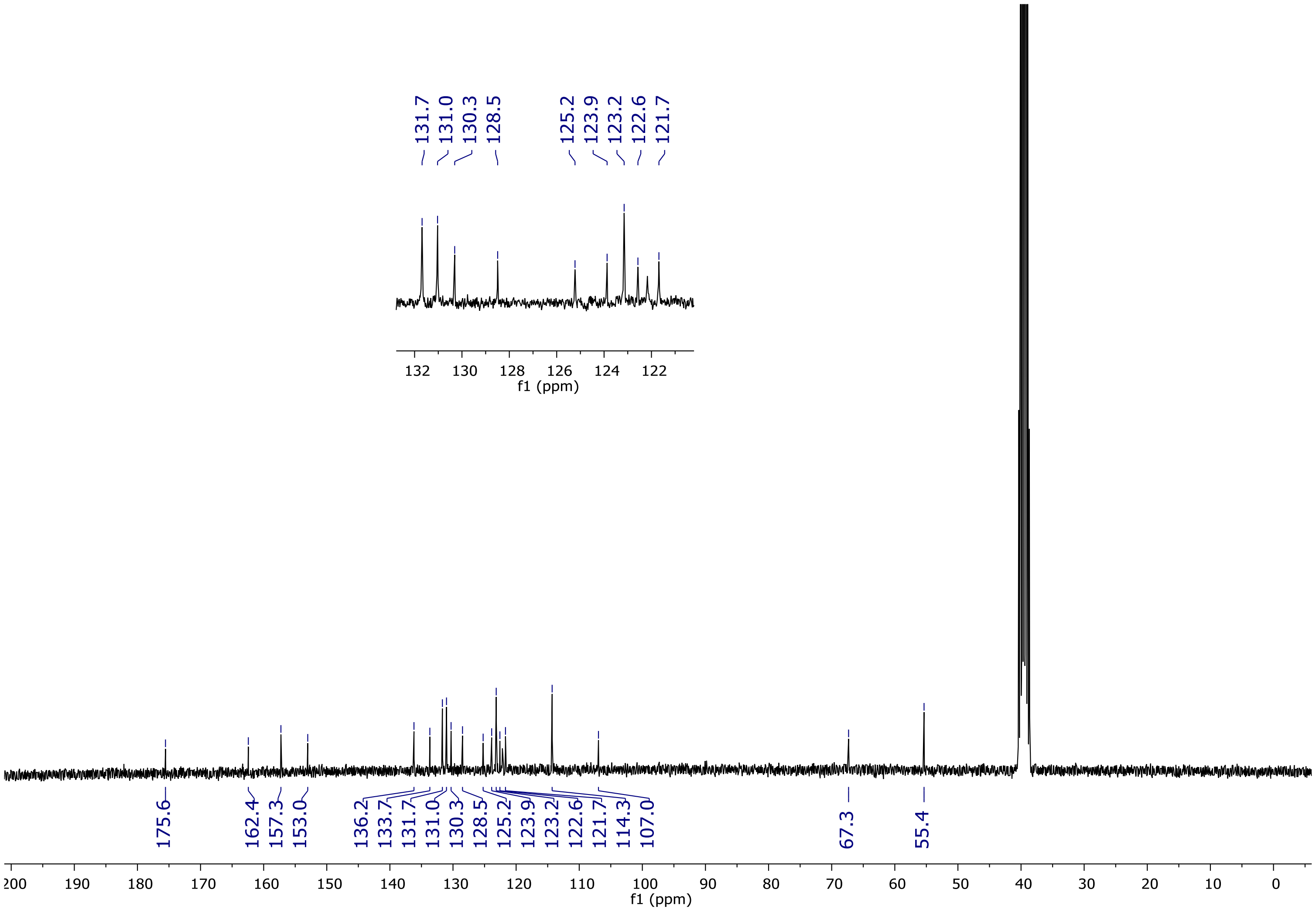

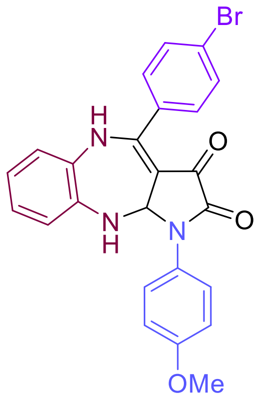

5x

Figure S53. $^{13}C\{^1H\}$ NMR spectrum of **5x** (DMSO-d_6, 75.46 MHz)

5. References

(1) Perrin, D. D.; Armarego, L. F. In Purification of Laboratory Chemicals, Pergamon Press, New York, 3rd ed, 1996.

(2) Rosa, F. A.; Machado, P.; Rossatto, M.; Vargas, P. S.; Bonacorso, H. G.; Zanatta, N.; Martins, M. A. P. *Synlett* **2007**, *20*, 3165-3171.

(3) Korin, E.; Cohen, B.; Bai, Y-X.; Zeng, C-C.; Becker, J. Y. *Tetrahedron*, **2012**, *68*, 7450-7455.

(4) da Silva, M. J. V.; Poletto, J.; Jacomini, A. P.; Pianoski, K. E.; Gonçalves, D. S.; Ribeiro, G. M.; Melo, S. M. S.; Back, D. F.; Moura, S.; Rosa, F. A. *J. Org. Chem*. **2017**, *82*, 12590-12602.

(5) Poletto, J.; Ribeiro, G. M.; da Silva, M. J. V.; Jacomini, A. P.; Basso, E. A.; Back, D. F.; Moura, S.; Rosa, F. A. *Org. Lett*. **2019**, *21*, 6325-6328.

(6) Poletto, J.; da Silva, M. J. V.; Pianoski, K. E.; Willig, J. C. M.; Rosa, F. A. *J. Org. Chem*. **2022**, *87*, 8544-8550.
